# Use of serotonin reuptake inhibitor antidepressants and the risk of bleeding complications in patients on anticoagulant or antiplatelet agents: a systematic review and meta-analysis

**DOI:** 10.1080/07853890.2021.2017474

**Published:** 2021-12-27

**Authors:** Surapon Nochaiwong, Chidchanok Ruengorn, Ratanaporn Awiphan, Chatree Chai-Adisaksopha, Apichat Tantraworasin, Chabaphai Phosuya, Penkarn Kanjanarat, Wilaiwan Chongruksut, Manish M. Sood, Kednapa Thavorn

**Affiliations:** a Department of Pharmaceutical Care, Faculty of Pharmacy, Chiang Mai University, Chiang Mai, Thailand; b Pharmacoepidemiology and Statistics Research Center (PESRC), Faculty of Pharmacy, Chiang Mai University, Chiang Mai, Thailand; c Division of Hematology, Department of Internal Medicine, Faculty of Medicine, Chiang Mai University, Chiang Mai, Thailand; d Department of Surgery, Faculty of Medicine, Chiang Mai University, Chiang Mai, Thailand; e Ottawa Hospital Research Institute, Ottawa Hospital, Ottawa, Canada; f Division of Nephrology, Department of Medicine, University of Ottawa, Ottawa, Canada; g School of Epidemiology and Public Health, Faculty of Medicine, University of Ottawa, Ottawa, Canada

**Keywords:** Anticoagulation, antidepressant, antiplatelet, bleeding complications, meta-analysis, serotonin-reuptake inhibitors

## Abstract

**Background:**

Serotonin reuptake inhibitor (SRI) antidepressants are implicated in increasing the risk of bleeding among users; however, the comparative increase in bleeding risk with concurrent antithrombotic therapy (anticoagulant or antiplatelet) remains unclear. As such, we performed a systematic review and meta-analysis of all available evidence to evaluate the effects of SRI and the risk of bleeding complications among patients receiving antithrombotic therapy.

**Methods:**

We searched Medline, Embase, PubMed, PsycINFO, Cochrane Library, Web of Science, Scopus, CINAHL, and grey literature (Google Scholar and preprint reports) up to 26 November, 2020, with no language restrictions (updated on 31 July 2021). The primary outcome of interest was major bleeding. Secondary outcomes included intracranial haemorrhage, gastrointestinal bleeding, and any bleeding events. We used a random-effects model meta-analysis to estimate the odds ratios (ORs) and 95% confidence intervals (CIs).

**Results:**

We did not identify any randomised studies but found 32 non-randomized studies (cohort or case–control) with 1,848,285 patients that fulfilled the study selection criteria and were included in the meta-analysis. Among individuals receiving anticoagulants (13 studies), SRI users experienced a statistically higher risk of major bleeding compared to non-SRI users: pooled OR was 1.39 (95% CI, 1.23–1.58; *p* < .001; moderate heterogeneity). Among individuals receiving antiplatelet therapy (2 studies), SRI users were associated with an increased risk of major bleeding: pooled OR was 1.45 (95% CI, 1.17–1.80; *p* = .001; low heterogeneity). For secondary outcomes, the use of SRI among individuals treated with antithrombotic therapy revealed a higher risk of gastrointestinal bleeding or any bleeding events, whereas only anticoagulant use was illustrated an increased risk of intracranial haemorrhage.

**Conclusions:**

The use of SRI antidepressants among patients treated with antithrombotic therapy (either anticoagulant or antiplatelet) is associated with a higher risk of bleeding complications, suggesting that caution is warranted in co-prescription.

**PROSPERO Registration:**

CRD42018083917

**KEY MESSAGES**
- In this meta-analysis of 32 non-randomized studies, SRI users were associated with the risk of bleeding complications compared to non-SRI users, with concurrent antithrombotic use (either anticoagulant or antiplatelet).
- The risk was consistently elevated across types of bleeding events (major bleeding, gastrointestinal bleeding, or any bleeding events), whereas only anticoagulant use was associated with intracranial haemorrhage.
- To promote the rational use of medicines, our findings suggest that the risk-benefit ratio must account for the clear efficacy of SRI against safety concerns in terms of bleeding risks.

## Introduction

Serotonin reuptake inhibitors (SRIs), including selective serotonin reuptake inhibitors (SSRI) and serotonin-norepinephrine reuptake inhibitors (SNRI) are the most widely prescribed antidepressants that are used in various psychiatric settings including cardiac patients [1]. With respect to the favourable safety profiles compared to older generations of antidepressants, SRI antidepressants and antithrombotic agents (anticoagulants and antiplatelet) are often prescribed together as depression and anxiety often coexist with cardiovascular/cerebrovascular diseases, atrial fibrillation, myocardial infarction, and other thromboembolic disorders [2,3]. Besides the risk of bleeding complications among antithrombotic therapy, recent accumulating evidence suggests that SRI use may be associated with an increased risk of bleeding, intracranial haemorrhage, and in particular, gastrointestinal tract bleeding [4–7]. In addition, concurrent use of SRI may potentiate this risk of bleeding complications further *via* pharmacokinetics or pharmacodynamics drug interactions. Specifically, concurrent use of SRI and antithrombotic appear to have the potential to inhibit cytochrome P450 (CYP) isoforms metabolism and impair serotonin platelet function [8].

Although several existing epidemiological studies have recognized the increased risk of bleeding complications among patients who received SRI, the safety of their use concomitant with antithrombotic therapy has not been fully elucidated. Moreover, previous systematic reviews have focussed on the use of SRI concomitant with non-steroidal anti-inflammatory drugs (NSAIDs), with the majority of those studies investigating gastrointestinal tract bleeding risk [6,7,9]. To the best of our knowledge, no comprehensive systematic review and meta-analysis has yet been conducted to quantify the effects of SRI use concomitantly with antithrombotic therapy and the risk of bleeding complications. To address this knowledge gap, we aimed to systematically review and summarize all available evidence to evaluate the effects of SRI use and the risk of bleeding complications among patients who received antithrombotic anticoagulants or antiplatelet therapy.

## Materials and methods

This systematic review and meta-analysis were performed and reported in line with the Preferred Reporting Items for Systematic Reviews and Meta-Analyses guidelines [10] and the Meta-analysis of Observational Studies in Epidemiology statement [11]. The pre-specified protocol was registered in the PROSPERO International prospective register of systematic reviews (CRD42018083917).

### Data sources and search strategy

In collaboration with an experienced medical librarian, we searched electronic databases, including Medline, Embase, PubMed, PsycINFO, Cochrane Library (CENTRAL), Web of Science, Scopus, and CINAHL from inception to 26 November 2020 with no language restrictions. Grey literature from Google Scholar and the preprint reports (medRxiv, bioRxiv, and PsyArXiv) were supplemented to the electronic database searches to identify all relevant articles. We used combinations of Medical Subject Headings and search terms including pharmacological class or individual drugs (i.e. “antithrombotic” or “anticoagulant” or “antiplatelet”, AND “serotonin uptake inhibitor” or “SSRI” or “SNRI”) and bleeding complications (i.e. “bleeding” or “haemorrhage” or “blood transfusion”). The full search strategy for each database is available in the Supplementary, eTable 1. Relevant articles were also browsed from the reference lists of the included studies, previous systematic reviews, and major international pharmacoepidemiology/cardiology/psychiatry congresses. To update the search, a targeted manual search of relevant articles was performed through to 31 July 2021.

### Study selection and outcomes

Eligible titles and abstracts of articles identified were screened independently by two reviewers (SN and CR). Then, potentially relevant full-text articles were assessed against the selection criteria for the final set of included studies. Potentially eligible articles that were not written in English were translated before the full-text appraisal. Any disagreement was resolved by a team discussion.

We included both randomized controlled trials (RCTs) and non-randomized studies (cohort or case-control) that (i) investigated the association between the use of SRI and risk of bleeding complications among adult patients (aged 18 years or more) receiving antithrombotic therapy (anticoagulant or antiplatelet agents) for any indications; (ii) consisted of two or more groups in which one group represented the use of SRI concomitant with antithrombotic therapy; (iii) consisted of SRI users including SSRI (i.e. citalopram, escitalopram, fluoxetine, fluvoxamine, paroxetine, and sertraline), SNRI (i.e. desvenlafaxine, duloxetine, milnacipran, and venlafaxine), or mixed action antidepressant agents (i.e. bupropion, mirtazapine, and trazodone); (iv) reported bleeding complications or provided sufficient data to calculate the risk estimate. We excluded studies that (i) were case series/case reports, N-of-one, cross-sectional, reviews, or studies with small sample sizes (less than 50 patients); and (ii) had no control group. Details of the selection criteria are provided in the Supplementary, eTable 2. For the companion study that included overlapping patients and study periods, the study with the most detailed and relevant information was included.

The primary outcome of interest was major bleeding, defined according to the International Society on Thrombosis and Haemostasis [12,13]. Secondary outcomes of interest included intracranial haemorrhage, gastrointestinal bleeding, and any bleeding events. Additional secondary outcomes included blood transfusion, endoscopy-refractory bleeding, rebleeding, and bleeding-related mortality. Based on clinical relevance, we defined the outcomes according to each included study. For instance, gastrointestinal bleeding events that required hospitalisation or related to mortality were considered major bleeding events.

### Data extraction and quality assessment

Two reviewers (SN and RA) independently extracted the following pre-specified data using a standardized approach to gather information on (i) the study characteristics (the first author’s name, study design [RCTs, cohort, case–control], study population, sample size, study country, study period, analysis method, and factors controlled for analysis); (ii) patient characteristics (mean or median age of study population, the proportion of females, and comorbid conditions); (iii) specific exposure and control groups (definition of SRI users and non-SRI users, SRI dosage, and concomitant medications); and (iv) predefined outcomes of interest (including assessment outcome definitions and outcome measurements). Studies with incomplete data or unclear information were clarified by the corresponding author. In cases where authors did not respond after two attempts of contact, we used information reported to calculate the required data or excluded the study in the analyses. The final set of data was cross-checked independently by one reviewer (CP and WC).

A pair reviewer (SN and CR) independently assessed and appraised the methodological quality of each included study using the Cochrane revised tool for assessing the risk of bias in randomised trials (RoB 2) [14] and the Newcastle–Ottawa Scale (NOS) for assessing the quality of included non-randomised studies [15]. The overall risk of bias of included studies was then classified into low, high, or some concerns for randomized trials (RoB 2), and the highest quality, if the summary score of the NOS was 8 or more points, for non-randomized studies. Moreover, we also used the Risk of Bias in Non-Randomized Studies of Interventions (ROBINS-I) tool to assess the risk of bias and categorized the overall judgement as low, moderate, serious, or critical risk of bias [16]. To interpret our findings, the strength of evidence for each outcome was critically appraised independently by a pair of reviewers (SN and CR) using the Grading of Recommended Assessment, Development, and Evaluation (GRADE) guidelines [17]. The strength of a body of evidence findings was then classified into very low-, low-, moderate-, or high-quality. Any discrepancies were addressed by team discussion.

### Statistical analysis

Two-tailed with a *P*-value of less than .05 was considered statistically significant. All analyses and generated forest plots of the summary pooled effects estimate were performed using Stata software version 16.0 (StataCorp, College Station, TX, USA). Inter-rater agreements were tested using kappa (κ) statistics to assess the agreement between reviewers in the study selection and risk of bias assessment processes. Based on the common risk estimates across the included studies, we used the aggregate odds ratios (ORs) with the greatest degree of adjustment for potential confounding factors as the summary effect estimates of association for each outcome of interest. As the methodological approach varied across included studies, we employed the random-effects models using the DerSimonion–Laird method for estimating the pooled ORs with corresponding 95% confidence intervals (CIs) to account for heterogeneity between studies [18].

Heterogeneity was assessed using the Cochran *Q* test, with a *P*-value of less than 0.10. The degree of inconsistency was investigated using *I*^2^ and tau-squared (*τ*^2^) statistics, [19,20] in which the heterogeneity was estimated as low (*I*^2^=25.0%, *τ*^2^=0.01), moderate (*I*^2^=50.0%, *τ*^2^=0.06), and high (*I*^2^=75.0%, *τ*^2^=0.16). We tested publication bias using Begg’s and Egger’s tests for each specific outcome of interest (*P*-value of less than .10 indicated statistical publication bias) [21,22]. The visual inspection of funnel plots was also performed where there was sufficient data to explore for asymmetry of the funnel graph. Moreover, the trim and fill method was then performed to calibrate for publication bias and account for the number of studies with null effects which were missing from the meta-analysis [23].

Pre-planned subgroup analyses were conducted based on (i) patient characteristics (i.e. age, proportion of males, history of bleeding events, comorbid conditions [atrial fibrillation, diabetes, chronic heart failure, coronary artery disease, renal failure, cancer, and *Helicobacter pylori* infection], and concomitant medications [use of NSAIDs, corticosteroids, and gastroprotective agents]); and (ii) study characteristics (sample size [less than 5000 vs. 5000 or more], study design (RCTs, cohort, or case-control), and study location (North America vs. non-North America). If data were available, individual SRI use and dosage were also assessed to establish the evidence of a dose–response and duration–response relationship.

A set of sensitivity analyses were conducted to assess the robustness of primary findings, including (i) restricting analysis to studies that adjusted for key confounding factors (age, sex, and history of bleeding); (ii) restricting the analysis to studies with high quality; (iii) limiting the analysis to studies with the directness of effect estimates; (iv) removing unpublished studies; (v) removing individual study approaches (leave one out analysis); and (vi) using the fixed-effects models if the *I*^2^ index less than 25.0%. Additionally, a random-effects univariate meta-regression was also performed according to the level of risk of bias, study characteristics, and baseline patient characteristics to explore the pre-specified effects on the meta-analytic estimates.

## Results

**Figure 1.**
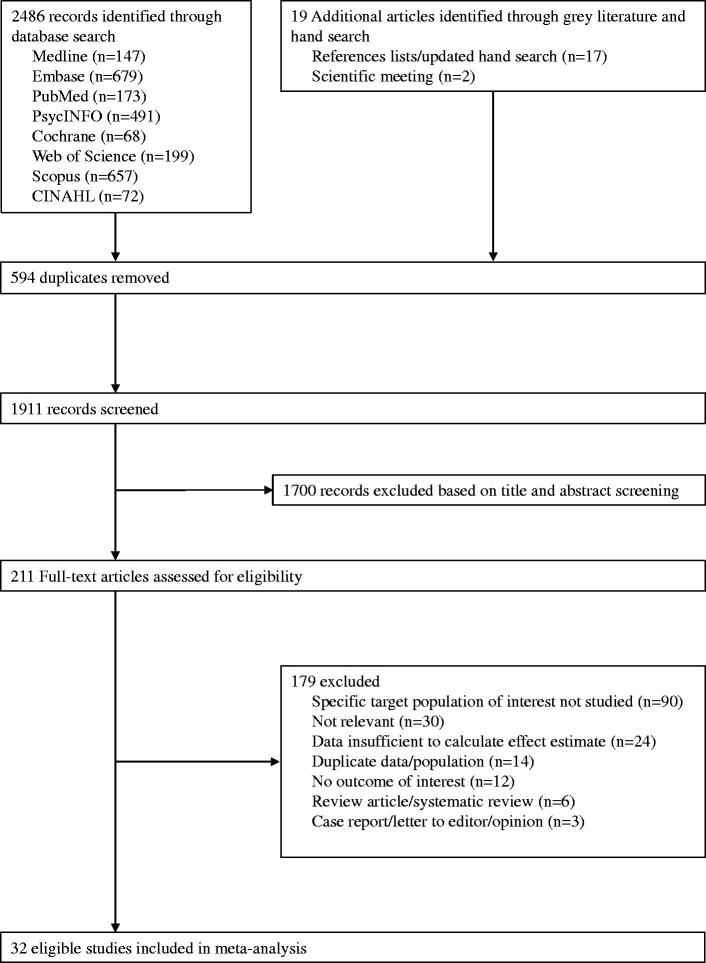
Study selection flowchart.

The search strategy retrieved 2505 records. From these, 594 duplicate records were removed, and 1911 records remained. Based on the title and abstract screening, we identified 211 articles that seemed to be relevant to the study question. Of these, 32 non-randomized studies fulfilled the study selection criteria and were included in the meta-analysis, while we did not identify any clinical randomised trials (Figure 1). The inter-rater agreement between reviewers on the study selection and data extraction was 0.87 and 0.79, respectively.

Table 1 summarizes the characteristics of all the included studies. In total, 1,848,285 patients were identified with a mean age ranging from 52.4 to 82.4 years, proportion of male sex ranging from 22.5% to 79.0%, and most of the included studies not providing a specific indication of the use of SRI and antithrombotic therapy. Detailed measurement and definition of bleeding events, methodology for the study, comorbid conditions, and concomitant medications of the included studies are described in Supplementary, eTables 3, 4, and 5, respectively. According to the risk of bias assessed in 32 non-randomised included studies (Supplementary, eTable 6), summary scores ranged from 3 to 9 points, with 19 studies (59.4%) having the highest quality (NOS of 8 or more). Based on the ROBINS-I tool, we found that most included studies had a moderate risk of bias (21 studies, 65.6%); however, no study with critical risk of bias was observed (Supplementary, eTable 6). The summary results and strength of evidence findings are provided in Table 2. Details of evidence synthesis by the GRADE system are provided in Supplementary, eTable 7.

**Table 1.** Baseline characteristics of the included studies in the meta-analysis.

Author, year	Study design	Country	Sample size	Population with antithrombotic therapy	Study period	Age in years, mean ± SD	Male sex, n (%)	Exposure: SRI antidepressants	Outcomes
Kurdyak et al. [43], 2005	Nested case-control	Canada	16734	Elderly patients (>65 years) treated with warfarin fo*r* ≥ 1 years	January 1994– December 2002	80.8 ± 6.6	NR	SSRIs: citalopram, fluoxetine, fluvoxamine, paroxetine, sertraline	Gastrointestinal bleeding (UGIB)
Kharofa et al. [39], 2007^†^	Case–control	United States	2692	Treated with aspirin 2 weeks before index date	May 1997– October 2005	Range: 50.9–70.2	NR	SSRIs: citalopram, escitalopram, fluoxetine, paroxetine, sertraline	Brain haemorrhage (ICH and SAH)
de Abajo et al. [44], 2008^†^	Nested case–control	United Kingdom	11321	Current use of antiplatelet agents (primary low-dose aspirin) or oral anticoagulants (within 0–30 days of index date)	January 2000– December 2005	40–59 (27.2%) 60–69 (21.6%) 70–79 (34.7%) 80–84 (16.5%)	6446 (56.9%)	SSRIs: citalopram, escitalopram, fluoxetine, fluvoxamine, paroxetine, sertraline SNRIs: duloxetine, venlafaxine	Gastrointestinal bleeding (UGIB)
Schalekamp et al. [24], 2008	Nested case–control	The Netherlands	7666	New users of coumarin: acenocoumarol (90.4%) and phenprocoumon (9.6%)	January 1991– December 2004	72.8 ± 9.8	4166 (54.3%)	SSRIs: citalopram, escitalopram, fluvoxamine, fluoxetine, paroxetine, sertraline	Major bleeding, Brain haemorrhage (ICH), Gastrointestinal bleeding
Dall et al. [49], 2009^†^	Case–control	Denmark	40154	Current use of aspirin (within the past 90 days)	August 1995– July 2006	72.1 ± 14.1	20541 (51.2%)	SSRIs: citalopram, escitalopram, fluoxetine, fluvoxamine, paroxetine, sertraline	Gastrointestinal bleeding (UGIB)
Wallerstedt et al. [25], 2009	Retrospective cohort	Sweden	234	Treated with warfarin due to AF	January 1999– September 2005	72.0 ± 7.0	122 (52.1%)	SSRIs: citalopram, escitalopram, fluoxetine, fluvoxamine, paroxetine, sertraline	Major bleeding
Cochran et al. [26], 2011^†^	Retrospective cohort	United States	100	Treated with warfarin in an outpatients fo*r* ≥ 6 months	January 2007– November 2009	58.5 ± 16.0	25 (25.0%)	SSRIs: citalopram, escitalopram, fluoxetine, paroxetine, sertraline	Major bleeding, any bleeding
Labos et al. [37], 2011	Retrospective cohort	Canada	27058	ACS with antiplatelet therapy: aspirin, clopidogrel, DAPT (aspirin and clopidogrel)	January 1998– March 2007	72.7 ± 10.6	19087 (70.5%)	SSRIs: citalopram, escitalopram, fluoxetine, fluvoxamine, paroxetine, sertraline	Major bleeding, Gastrointestinal bleeding
Schelleman et al. [45], 2011	Nested case–control	United States	666235	Treated with warfarin	January 1999 – December 2005	18–50 (12.9%) 51–60 (12.2%) 60–70 (18.9%) 70–80 (27.5%) ≥81 (28.5%)	242984 (36.5%)	SSRIs: citalopram, escitalopram, fluoxetine, paroxetine, sertraline SNRIs: venlafaxine	Gastrointestinal bleeding
Vitry et al. [27], 2011	Retrospective cohort	Australia	17661	Veterans who were new users of warfarin	July 2002– June 2006	81.8 ± 4.4	11277 (63.8%)	SSRIs: not specified	Major bleeding
Baillargeon et al. [28], 2012	Nested case–control	United States	3192	Treated with warfarin for at least 180 days	January 2007 – December 2008	66–70 (10.4%) 71–75 (19.3%) 76–80 (22.2%) 81–85 (22.6%) ≥86 (25.5%)	1116 (35.0%)	SSRIs: citalopram, escitalopram, fluoxetine, fluvoxamine, paroxetine, sertraline SNRIs: desvenlafaxine, duloxetine, milnacipran, venlafaxine	Major bleeding
Lin et al. [50], 2013	Retrospective cohort	Taiwan	3238	Treated with clopidogrel with an average dose o*f* > 150 DDD per one-half year	January 2001 – December 2010	68.6 ± 11.6	1899 (58.6%)	SSRIs: not specified	Gastrointestinal bleeding (UGIB, LGIB)
Mosholder et al. [29], 2013	Retrospective cohort	United States	324356	Treated with warfarin for at least 1 months	June 2006– October 2010	<65 (13.0%) 65–74 (28.2%) 75–84 (37.4%) >84 (21.4%)	213803 (65.9%)	SSRIs: not specified	Major bleeding, Brain haemorrhage (ICH), Gastrointestinal bleeding
Seitz et al. [54], 2013^‡^	Retrospective cohort	Canada	8568	Treated with antiplatelet agents or warfarin in the 120 days preceding index	April 2003 – December 2009	82.4 ± 7.0	1927 (22.5%)	Current users of high-affinity SRIs: citalopram, escitalopram, clomipramine, duloxetine, fluoxetine, fluvoxamine, paroxetine, sertraline, venlafaxine	Perioperative blood transfusion
Giang et al. [52], 2014	Retrospective cohort	United States	162	Treated with DAPT (aspirin and P2Y12 inhibitors) following coronary stent placement	October 2010 – January 2012	NR	NR	SSRIs: citalopram, fluoxetine, sertraline	Any bleeding
Nguyen et al. [48], 2014	Retrospective cohort	United States	3153	Veterans who were prescribed warfarin	October 2009 – September 2011	NR	NR	SSRIs: not specified	Any bleeding
Quinn et al. [30], 2014^†^	Retrospective cohort	United States	9186	Treated AF with warfarin among the ATRIA study	Diagnosed AF from Jul 1996 – Dec 1997, and followed up to 6 years	≥75 (53.3%)	5337 (58.1%)	SSRIs: citalopram, escitalopram, fluoxetine, fluvoxamine, paroxetine, sertraline SNRIs: venlafaxine	Major bleeding, Brain haemorrhage
Rashid et al. [38], 2016	Retrospective cohort	Australia	839	ACS underwent angioplasty and received DAPT	January 2014 – December 2015	61.8 ± 12.5	663 (79.0%)	SSRIs: not specified	Major bleeding, any bleeding
Lai et al. [46], 2017	Retrospective cohort	United States	21503	Treated with DOACs: apixaban (25.3%), dabigatran (25.9%), rivaroxaban (48.5%)	November 2010 – December 2015	18–64 (22.5%) 65–74 (30.7%) ≥75 (46.8%)	11597 (53.9%)	SSRIs: not specified	Gastrointestinal bleeding
Laursen et al. [55], 2017^†^	Prospective registry	Denmark	14343	Treated with low-dose aspirin (≤150 mg/d)	August 2006 – August 2014	75.0 ± 27.6	7727 (53.9%)	SSRIs: not specified	Endoscopy-refractory bleeding, re-bleeding in peptic ulcer bleeding
Renoux et al. [40], 2017^†^	Nested case–control	United Kingdom	92738	Current use of antiplatelet agents or oral anticoagulants ( within 1 month before index date)	January 1995– June 2014	66.6 ± 16.6	36305 (39.1%)	SSRIs: citalopram, escitalopram, fluoxetine, fluvoxamine, paroxetine, sertraline	Brain haemorrhage (ICH)
Samuel et al. [31], 2017	Retrospective cohort	United States	575	Primary or secondary diagnosis of an VTE and treated with full dose enoxaparin	October 2009 – October 2014	59.0 ± 38.3	310 (53.9%)	SSRIs: citalopram, escitalopram, fluoxetine, fluvoxamine, paroxetine, sertraline	Major bleeding
Scheitz et al. [41], 2017	Prospective registries	Finland, France, Germany, Netherlands, Switzerland	6242	Preadmission with anticoagulants among acute ischaemic stroke patients treated by thrombolysis	June 1998–August 2016	70.1 ± 14.0	3501 (56.1%)	SSRIs: citalopram, escitalopram, fluoxetine, fluvoxamine, paroxetine, sertraline	Brain haemorrhage (post-thrombolysis symptomatic ICH)
Quinn et al. [32], 2018^§^	Prospective cohort: using data from the ROCKET AF trial	International collaboration	1474	AF patients treated with rivaroxaban or warfarin for the prevention of stroke/systematic embolism	December 2006– June 2009	73.8 ± 8.5	703 (47.7%)	SSRIs: citalopram, escitalopram, fluoxetine, fluvoxamine, paroxetine, sertraline SNRIs: desvenlafaxine, duloxetine, venlafaxine	Major bleeding, any bleeding
Iasella et al. [53], 2019	Retrospective cohort	United States	6819	Treated with DAPT (clopidogrel-based) after PCI	July 2010– December 2014	66.8 ± NR	4516 (66.2%)	SSRIs: citalopram, escitalopram, fluoxetine, fluvoxamine, paroxetine, sertraline	Any bleeding
Luo et al. [51], 2019	Retrospective cohort	Taiwan	11105	Treated with aspirin with an average dose o*f* > 14 DDD per month	January 2001 – December 2010	64.0 ± 12.7	5864 (52.8%)	SSRIs: not specified	Gastrointestinal bleeding (UGIB)
Gaist et al. [42], 2020^†^	Case–control	Denmark	446264	Current use (supply with grace period extended [60 days] up to cover index date) of antiplatelet agents (low-dose aspirin, clopidogrel) or oral anticoagulants (phenprocoumon, warfarin, apixaban, dabigatran, rivaroxaban)	January 2000– December 2016	71.3 ± 14.8	290280 (65.0%)	SSRIs: citalopram, escitalopram, fluoxetine, fluvoxamine, paroxetine, sertraline	Brain haemorrhage (SDH)
Komen et al. [33], 2020	Retrospective cohort	Sweden	30595	AF patients with a new prescription of warfarin or DOACs	July 2011– December 2017	74.4 ± 11.0	17139 (56.0%)	Antidepressant: SSRIs (61.0%), TCA (11.3%), other (27.7%)	Major bleeding, Gastrointestinal bleeding, brain haemorrhage
Lee et al. [34], 2020	Nested case–control	Korea	25893	AF patients with a new prescription of DOACs (apixaban, rivaroxaban, edoxaban, dabigatran)	January 2013 – December 2017	76.3 ± 9.1	11949 (46.1%)	SSRIs: escitalopram, fluoxetine, fluvoxamine, paroxetine, sertraline	Major bleeding, Gastrointestinal bleeding (UGIB, LGIB), brain haemorrhage (ICH)
Marchena et al. [35], 2020	Prospective registry	Spain	47050	Adult patients receiving anticoagulant therapy for VTE (VKAs, LMWH, DOACs)	February 2009– September 2019	66.5 ± 17.8	23999 (51.0%)	SSRIs: citalopram, escitalopram, paroxetine, sertraline, SNRIs: duloxetine, venlafaxine Mixed: mirtazapine, trazodone	Major bleeding, brain haemorrhage (ICH)
Mawardi et al. [47], 2020	Retrospective cohort	United States	248	LVAD patients treated with warfarin and aspirin (81 mg or 325 mg)	January 2009 – January 2016	52.4 ± 8.4	142 (57.2%)	SSRIs: citalopram, escitalopram, fluoxetine, paroxetine, sertraline SNRIs: duloxetine, venlafaxine Mixed: bupropion, mirtazapine, trazodone	Gastrointestinal bleeding
Zhang et al. [36], 2020	Nested case–control	United Kingdom	1887	Adult patients with new users of DOACs (dabigatran, apixaban, rivaroxaban)	January 2008– December 2015	78.7 ± 10.2	1175 (62.3%)	SSRIs: citalopram, escitalopram, fluoxetine, nefazodone, paroxetine, sertraline, SNRIs: venlafaxine, duloxetin	Major bleeding, Gastrointestinal bleeding

^†^On the basis of the entire study population.

^‡^On the basis of the current and former serotonergic users.

^§^On the basis of the propensity-score matching.

ACS: acute coronary syndrome; AF: atrial fibrillation; ATRIA: AnTicoagulation and Risk factors In Atrial fibrillation; DAPT: dual antiplatelet therapy; DDD: defined daily dose; DOACs: direct oral anticoagulants; ICH: intracerebral haemorrhage; LMWH: low-molecular weight heparin); LVAD: left ventricular assist device; NR: not reported; PCI: percutaneous coronary intervention; ROCKET AF: Rivaroxaban once daily Oral direct factor xa inhibition Compared with vitamin K antagonism for prevention of Embolism and stroke Trial in Atrial Fibrillation; SAH: subarachnoid haemorrhage; SD: standard deviation; SDH: subdural haematoma; SRIs: serotonin reuptake inhibitors; LGIB: lower gastrointestinal tract bleeding; UGIB: upper gastrointestinal tract bleeding; VKAs: vitamin K antagonists; VTE: venous thromboembolism.

**Table 2.** Summary of findings and strength of evidence.

Bleeding complication	No. of included studies (Ref.)	No. of patients	Odds ratio (95% CI)	*p*-Value	Heterogeneity				Strength of evidence
					*Q* statistic	*p*-Value	*I^2^* index (95% CI)	*τ^2^*	
**Major bleeding**									
Anticoagulant therapy	13 (24–36)	469869	1.39 (1.23–1.58)	<0.001	31.27	0.005	55.2% (4.7–73.6)	0.026	Low (harm: increased risk)
Antiplatelet Therapy	2 (37, 38)	27897	1.45 (1.17–1.80)	0.001	1.08	0.782	0.0% (0.0–67.9)	<0.001	Very low (harm: increased risk)
**Intracranial haemorrhage**									
Anticoagulant therapy	10 (24, 29, 30, 33–35, 39–42)	443904	1.31 (1.02–1.68)	0.031	28.48	0.003	61.4% (11.8–77.9)	0.091	Very low (harm: increased risk)
Antiplatelet Therapy	3 (39, 40, 42)	81173	1.08 (0.93–1.26)	0.325	8.98	0.030	66.6% (0.0–86.4)	0.014	Very low (no harm)
**Gastrointestinal bleeding**									
Anticoagulant therapy	10 (24, 29, 33, 34, 36, 43–47)	1085014	1.34 (1.19–1.50)	<0.001	15.57	0.113	35.8% (0.0–67.2)	0.010	Low (harm: increased risk)
Antiplatelet Therapy	5 (37, 44, 49–51)	52571	1.30 (1.04–1.63)	0.021	5.93	0.204	32.6% (0.0–74.6)	0.021	Very low (harm: increased risk)
**Any bleeding events**									
Anticoagulant therapy	23 (24–36, 39–48)	1209421	1.39 (1.24–1.55)	<0.001	79.03	<0.001	68.4% (49.8–78.0)	0.040	Low (harm: increased risk)
Antiplatelet therapy	11 (37–40, 42, 44, 49–53)	153790	1.15 (1.06–1.25)	0.001	20.08	0.093	35.3% (0.0–64.6)	0.007	Low (harm: increased risk)

CI: confidence interval; NA: not applicable.

### Primary outcome: major bleeding

**Figure 2.**
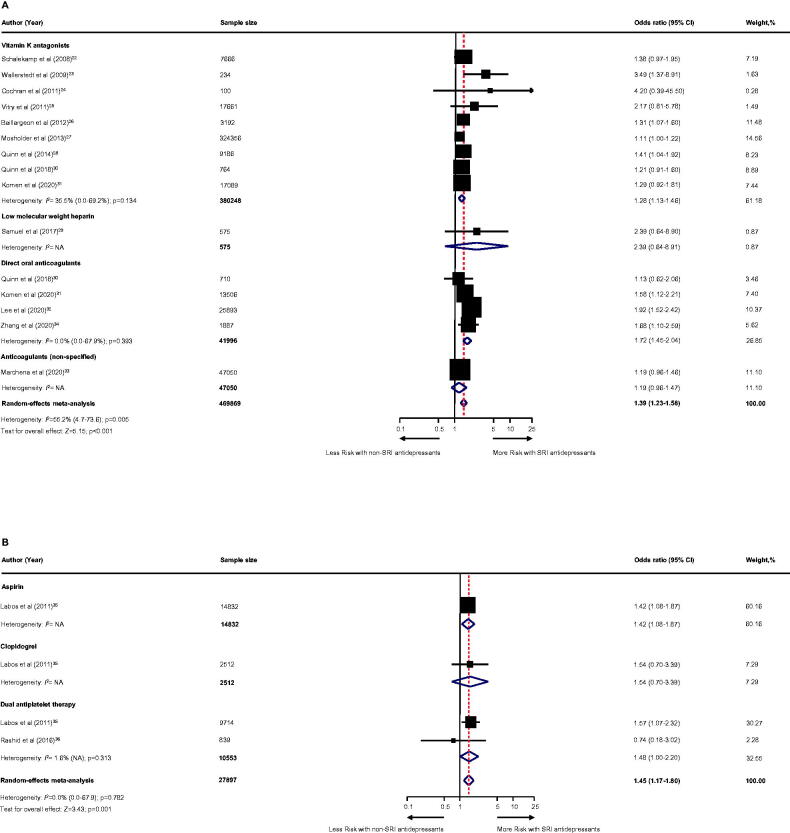
Effect of the use of SRI concomitant with antithrombotic therapy and the risk of major bleeding. Individuals treated with (A) anticoagulant therapy or (B) antiplatelet therapy. CI: confidence interval; NA: not applicable; SRI: serotonin reuptake inhibitor.

Among individuals receiving anticoagulant therapy (13 studies [24–36]; *n* = 469869; Figure 2(A)), SRI users experienced a statistically higher risk of major bleeding compared to non-SRI users with a moderate degree of heterogeneity: pooled OR was 1.39 (95% CI, 1.23–1.58; *p* < .001). With regard to anticoagulants (Figure 2(A)), the pooled OR was 1.28 (95% CI, 1.13–1.46; *p* < .001) for vitamin K antagonists (9 studies [24–30,32,33], *n* = 380,248); 2.39 (95% CI, 0.64–8.91; *p* = .194) for low-molecular-weight heparin (one study [31], *n* = 575); 1.72 (95% CI, 1.45–2.04; *p* < .001) for direct oral anticoagulants (4 studies [32–34,36], *n* = 41,996); and 1.19 (95% CI, 0.96–1.47; *p* = .104) for non-specified anticoagulants (one study [35]; *n* = 47,050).

Among individuals receiving antiplatelet therapy (two studies [37,38], *n* = 27,897; Figure 2(B)), SRI users were associated with an increased risk of major bleeding with a low degree of heterogeneity: pooled OR was 1.45 (95% CI, 1.17–1.80; *p* = .001). With regard to the use of antiplatelet (Figure 2(B)), the pooled OR was 1.41 (95% CI, 1.08–1.87; *p* = .012) for aspirin (one study [37], *n* = 14,832); 1.54 (95% CI, 0.70–3.39; *p* = .285) for clopidogrel (one study [37], *n* = 2512); and 1.48 (95% CI, 1.00–2.20; *p* = .050) for dual antiplatelet therapy (two studies [37,38], *n* = 10,553).

#### Secondary outcomes and additional secondary outcomes

**Figure 3.**
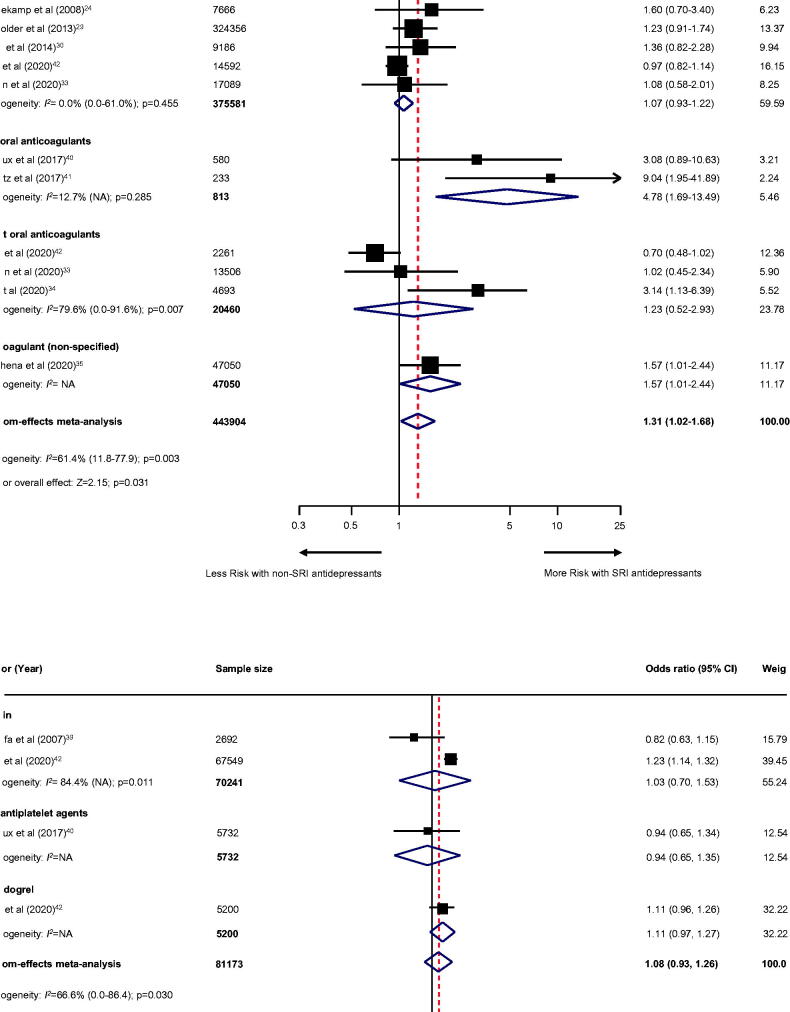
Effect of the use of SRI concomitant with antithrombotic therapy and the risk of intracranial haemorrhage. Individuals treated with (A) anticoagulant therapy or (B) antiplatelet therapy. CI: confidence interval; NA: not applicable; SRI: serotonin reuptake inhibitor.

**Figure 4.**
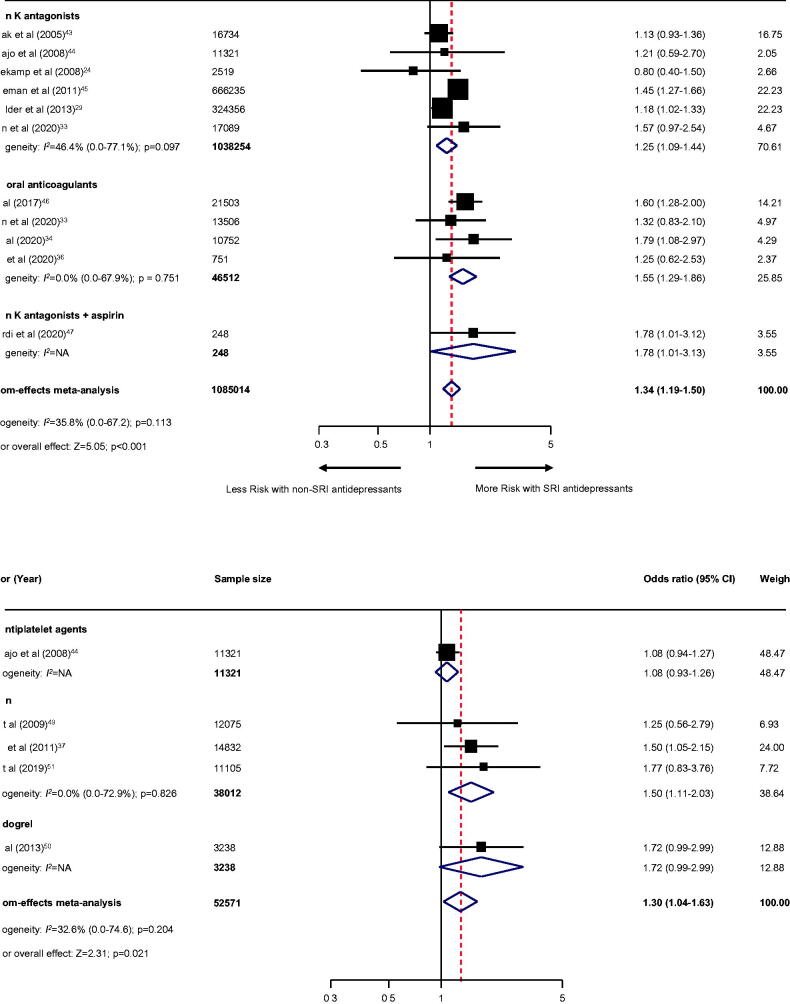
Effect of the use of SRI concomitant with antithrombotic therapy and the risk of gastrointestinal bleeding. Individuals treated with (A) anticoagulant therapy or (B) antiplatelet therapy. CI: confidence interval; NA: not applicable; SRI: serotonin reuptake inhibitor.

**Figure 5.**
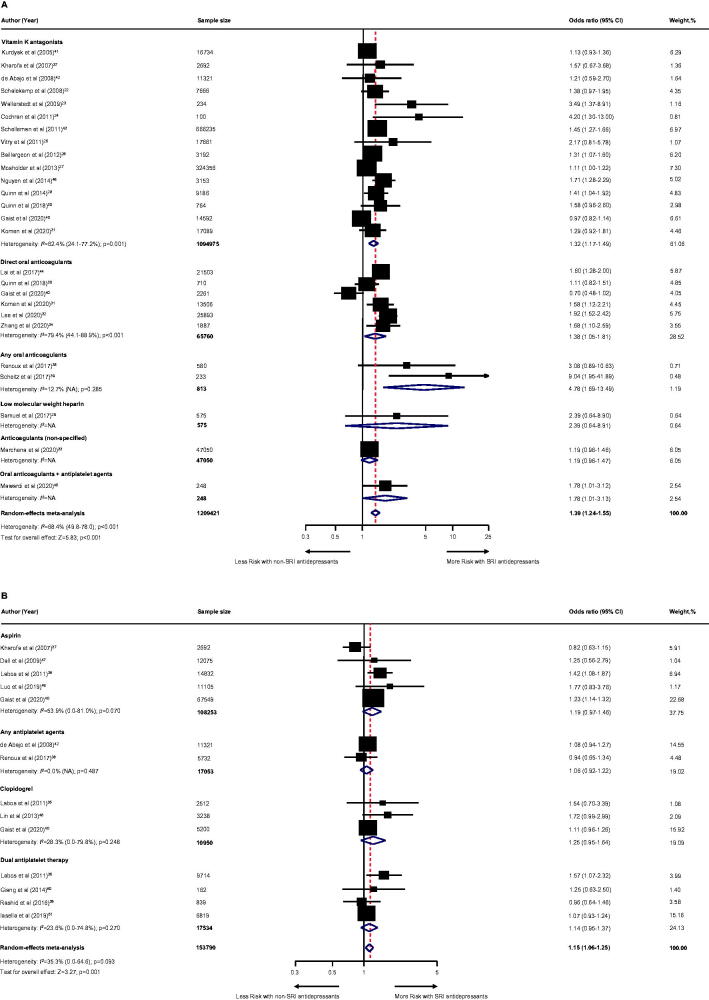
Effect of the use of SRI concomitant with antithrombotic therapy and the risk of any bleeding events. Individuals treated with (A) anticoagulant therapy or (B) antiplatelet therapy. CI: confidence interval; NA: not applicable; SRI: serotonin reuptake inhibitor.

For secondary outcomes, the use of SRI among individuals treated with anticoagulant therapy revealed a higher risk of intracranial haemorrhage (10 studies [24,29,30,33–35,39–42]; *n* = 443,904; OR, 1.31; 95% CI, 1.02–1.68; *p* = .031; Figure 3(A)), gastrointestinal bleeding (10 studies [24,29,33,34,36,43–47]; *n* = 1085014; OR, 1.34; 95% CI, 1.19–1.50; *p* < .001; Figure 4(A)), and any bleeding events (23 studies [24–36,39–48]; *n* = 1,209,421; OR, 1.39; 95% CI, 1.24–1.55; *p* < .001; Figure 5(A)). Likewise, use of SRI among individuals treated with antiplatelet agents also illustrated an increased risk of gastrointestinal bleeding (five studies [37,44,49–51]; *n* = 52571; OR, 1.30; 95% CI, 1.04–1.63; *p* = .021; Figure 4(B)), any bleeding events (11 studies [37–40,42,44,49–53]; *n* = 153,790; OR, 1.15 (95% CI, 1.06–1.25; *p* = .001; Figure 5(B)), except for intracranial haemorrhage (three studies [39,40,42]; *n* = 81173; OR, 1.08; 95% CI, 0.93–1.26; *p* = .325; Figure 3(B)).

Evidence for blood transfusion (one study [54]), endoscopy-refractory bleeding (one study [55]), and rebleeding (one study [55]) are inconclusive owing to the limited data available (Supplementary, eTable 8). However, no study has reported bleeding complications in terms of bleeding related to mortality. Moreover, risk estimates according to individual SRI use, as well as a dose- and duration-relationship cannot be established due to lack of information.

### Subgroup analyses

Several pre-planned subgroup analyses according to baseline patient characteristics and secondary outcomes, could not be performed due to the small sample size and limited information on outcomes across the included studies. Based on study characteristics, among individuals receiving anticoagulant therapy, the effect estimates between SRI use and the risk of bleeding complications was no longer statistically significant when the sample size was less than 5000 (for gastrointestinal bleeding), while the risk of intracranial haemorrhage was sensitive to sample size, study design, and study location (Supplementary, eTable 9). With a small number of included studies for individuals receiving antiplatelet therapy, no further association was observed, particularly among studies with a sample size less than 5000 or case–control study design (Supplementary, eTable 9).

### Sensitivity and meta-regression analyses

The set of sensitivity analyses was robust and did not change substantially from the main findings (Supplementary, eTables 10–15). However, there was no association after restricting the analysis to studies with high quality (NOS of 8 or more) for the risk of intracranial haemorrhage among individuals who received anticoagulation therapy (OR, 1.17; 95% CI, 0.91–1.51, Supplementary, eTable 11). According to the leave-one-out analysis (Supplementary, eTable 14), after the removal of individual studies by Renoux et al., 2017 [40], Scheitz et al. [41], Lee et al. [34], and Marchena et al. [35], there was no association between SRI use and the risk of intracranial haemorrhage among individuals receiving anticoagulant therapy. Meanwhile, the association between SRI users and intracranial haemorrhage among individuals receiving antiplatelet therapy became statistically significant after a study by Kharofa et al., 2007 [39] was omitted (OR, 1.16; 95% CI, 1.04–1.29). Moreover, removing the study by Labos et al., 2011 [37] and Lin et al., 2013 [50], resulted in no further association between the use of SRI and gastrointestinal bleeding risk among individuals who received antiplatelet therapy.

A univariate meta-regression was suitable for the primary outcomes (Supplementary, eTable 16). For individuals receiving anticoagulant therapy, the increased risk of bleeding complications was associated with the baseline proportion of NSAIDs use and the proportion of male sex for major bleeding and intracranial haemorrhage, respectively. Nonetheless, the heterogeneity of the included studies was not explained by any of the study characteristics, baseline patient characteristics, and the risk of bias among individuals receiving antiplatelet therapy.

### Publication bias

For individuals receiving anticoagulant therapy, evidence of publication bias related to the sample size was observed in the results of major bleeding, intracranial haemorrhage, and any bleeding, with the *P*-values tested for asymmetry less than .10. Asymmetry tests were observed in the results of intracranial haemorrhage and gastrointestinal bleeding among those who received antiplatelet therapy (Supplementary, eTable 17). The visually inspected funnel plots for each outcome are provided in the Supplementary, eFigure 1. However, after calibration for publication bias using the trim and fill method, the main findings were not substantially different. Notably, there was no longer an association between SRI use and the risk of intracranial haemorrhage and gastrointestinal bleeding among those who received anticoagulant and antiplatelet therapy, respectively, after the analysis was calibrated for publication bias (Supplementary, eTable 17).

## Discussion

This systematic review and meta-analysis of 32 included non-randomized studies showed low certainty evidence that SRI users experienced a statistically higher risk of bleeding complications compared to non-SRI users, particularly among patients treated with anticoagulant therapy. We found very low certainty of evidence on the association between SRI use and the risk of intracranial haemorrhage and gastrointestinal bleeding among patients who received anticoagulant and antiplatelet therapy, respectively.

Several mechanisms have been proposed to explain the association between SRI use and the risk of bleeding complications. Theoretically, it has been demonstrated that serotonergic antidepressants increase bleeding complications *via* inhibiting platelet aggregation [8]. Another possible explanation for SRI in relation to bleeding risk is increased gastric acid secretion directly by increasing the vagal tone, subsequently leading to potential ulcerogenic effects and gastrointestinal bleeding [8,56,57]. As expected, the use of SRI concomitant with antithrombotic therapy either anticoagulants or antiplatelet agents can aggravate the risk of bleeding *via* both pharmacokinetics or pharmacodynamics interactions [8]. Among individuals receiving SRI and warfarin, for instance, proposed drug–drug interactions that increase bleeding risk may include impairing platelet aggregation and CYP 450 inhibition of warfarin metabolism; the potency of CYP inhibition varied among SRI [8]. Furthermore, SRI may further decrease platelet or endothelial activation and reduce the efficiency of haemostasis beyond that associated with concomitant antiplatelet agents such as aspirin or clopidogrel [56].

Our findings expanded previous meta-analyses by providing insight into the impact of SRI use concomitant with antithrombotic therapy (both anticoagulants and antiplatelet agents) and the bleeding risk, which has not been fully addressed previously. With regard to the credibility of the evidence, a previous umbrella review by Dragioti et al. (2019 [58]) was based on highly suggestive evidence, which indicates an increased risk of bleeding complications among individual use of SSRI or SNRI users. The summary ORs of severe bleeding at any site and upper gastrointestinal bleeding were 1.41 (95% CI, 1.27–1.57) and 1.55 (95% CI, 1.35–1.78), respectively [58]. These findings are also supported by our results that the use of SRI among individuals who received antithrombotic therapy (particularly anticoagulation), included the risk of major bleeding, gastrointestinal bleeding, and any bleeding events. However, it is unclear whether the use of SRI among individuals receiving antithrombotic therapy may have additional intracranial haemorrhage. Several existing meta-analyses with substantial heterogeneity have illustrated an increased risk of intracranial haemorrhage among individuals receiving SRI that did not particularly focus on patients treated with antithrombotic therapy [59–61]. On the other hand, other studies have not supported this finding when restricting analyses to high-quality data [58,62]. Furthermore, our findings did not confirm this association when sensitivity analysis and publication bias were considered. Given the statistical power and the imprecision of our findings, evidence for the risk of intracranial haemorrhage among individuals’ use of SRI antidepressant concomitant antithrombotic therapy remains inconclusive.

### Strengths and limitations

The strengths of this study include a large sample size. We expanded and addressed the further risk of bleeding complications associated with the use of SRI among patients who received antithrombotic therapy, which had not been investigated by previous meta-analyses. From a methodological viewpoint, we used a rigorous and comprehensive systematic review approach, as well as extensive searching without language restriction. Furthermore, according to the set of sensitivity analyses, these results were consistent with the main analysis in most cases, suggesting the robustness of our findings.

This systematic review and meta-analysis have several limitations. First, despite conducting a comprehensive search strategy, data from RCT were not identified. Our findings relied on non-randomized observational studies, confounding by indication/contraindication, and unmeasured confounders must be noted. As a result, the causality of the use of SRI among patients who received antithrombotic therapy and the subsequent risk of bleeding complications cannot be established. Most of the studies included in this review were based on routinely collected administrative data and electronic health records, which could be prone to information bias. Second, on the basis of the NOS summary score, the quality of the included studies was varied; most cases (18 studies [56.2%]) had high quality (NOS more than 8 points) and 14 studies had low quality (NOS ranged from 3 to 7). Of these, four studies [38,46,48,52] (12.5%) were reported as abstracts, which could lead to incomplete information. However, the results after restricting the analysis to studies with high-quality or removing unpublished studies, according to the mentioned sensitivity analysis methods, yielded main findings that were not substantially different. Therefore, we advocate that future studies with high methodological quality are required. Third, disparities of individual SRI exposure and bleeding outcome definitions were observed across the included studies, which could have contributed to the moderate heterogeneity of our findings. Although the degree of inconsistency improved in most cases when subgroup analysis was performed, several pre-planned subgroup analyses could not be conducted due to the small number of included studies. Fourth, data on the individual use of SRI, key patient characteristics, and several confounding factors related to bleeding complications, such as renal function, history of bleeding events, or use of NSAIDs were not gartered across all included studies. As a result, a dose- and duration-relationship and risk effects estimate, based on the different subpopulations, cannot be established due to lack of information. Fifth, information on both SRI and antithrombotic therapy in terms of treatment medication and adherence over time were also lacking; thus, misclassification bias should be stated. Moreover, the data on pre-specified additional secondary outcomes, including, blood transfusion, endoscopy-refractory bleeding, rebleeding, and bleeding-related mortality were insufficient, which is an emerging concern with respect to the increased SRI use among individuals who received antithrombotic therapy and is needed for further studies. Finally, it is possible that publication bias exists and might account for some of the effect estimates we observed. Moreover, our strength of evidence findings using the GRADE approach was based on a low or very low body of evidence. Therefore, the interpretation of our findings should be exercised.

### Implications for practice and future research

Given the limited strength of the body of evidence, this systematic review and meta-analysis provides the best available evidence that can emerge as insight with respect to the use of SRI among individuals receiving antithrombotic therapy in general practice. In cardiac patients receiving antithrombotic drugs, the risk-benefit ratio must account for the clear efficacy of antidepressants against adverse health outcomes, which should be balanced with safety concerns in terms of bleeding risk. Thus far, individuals receiving combination therapy including SRI and antithrombotic therapy warrant proactive monitoring of bleeding complications, especially among individuals with a history of bleeding, or pre-existing risk of bleeding—that is, peptic ulcer disease, chronic liver disease, chronic kidney disease, or received concomitant medication that may further increase the risk of bleeding (i.e. NSAIDs). The findings from this review support the interventions or strategies that promote appropriate SRI prescriptions and minimise risk in relation to drug–drug interactions in real-world practice. In addition, patients should also be informed about the benefits and risks of concomitant SRI and antithrombotic therapy in terms of bleeding risks to promote the rational use of medicines.

Further research in RCTs alongside collaborative pharmacoepidemiology research and proactive real-world evidence surveillance systems are needed to reaffirm and clarify the potential causal association between SRI and risk of bleeding among individuals receiving antithrombotic therapy. Such research should elaborate on the use of individual SRI, clinical diagnoses and indications, pathogenesis and mechanistic processes, the severity of clinical and bleeding risk conditions, and dose–effect and duration–effect response.

## Conclusions

This systematic review and meta-analysis revealed that SRI use among patients treated with antithrombotic therapy, especially anticoagulants may increase the risk of bleeding complications, including major bleeding, gastrointestinal bleeding, and any bleeding events. However, these findings were limited by the nature of non-randomised included studies and the low strength of the body of evidence. However, evidence for intracranial haemorrhage or those who received SRI concomitant with antiplatelet therapy are inconclusive. Further pharmacoepidemiologic research, including proactive longitudinal surveillance systems, is needed to clarify and confirm the safety of using SRI in concomitance with antithrombotic therapy and the subsequent risk of bleeding complications.

## Ethical approval

Ethical approval was nor required as this study did not require use of patient identifiers.

## Supplementary Material

Supplemental Material

## Data Availability

All data generated or analysed during this study are included in this article and its supplementary information files. The data that support the findings of this study are available from the corresponding author upon reasonable request.
