# Supplementary material for: Use of serotonin reuptake inhibitor antidepressants and the risk of bleeding complications in patients on anticoagulant or antiplatelet agents: a systematic review and meta-analysis
Source: Ann Med. 2021 Dec 27;54(1):80–97. doi: 10.1080/07853890.2021.2017474 (PMC8725830; doi:10.1080/07853890.2021.2017474)
Supplement: Supplemental Material [file IANN_A_2017474_SM7515.zip › Supplementart files/Supplement_Checklist_Ann_Med.docx]

Meta-Analysis of Observational Studies in Epidemiology (MOOSE) statement Checklist

| **Item No** | **Recommendation** | **Reported on Page No** |
| --- | --- | --- |
| **Reporting of background should include** | | |
| 1 | Problem definition | Page 4 |
| 2 | Hypothesis statement | Page 4 |
| 3 | Description of the study outcomes | Page 6 |
| 4 | Types of exposure or intervention | Page 6 |
| 5 | Type of study designs used | Page 6 |
| 6 | Study population | Page 6 |
| **Reporting of search strategy should include** | | |
| 7 | Qualifications of searchers (eg. librarians and investigators) | Page 5, eMethods |
| 8 | Search strategy, including time period included in the synthesis and keywords | Page 5, Table S2 |
| 9 | Effort to include all available studies, including contact with authors | eMethods |
| 10 | Databases and registries searched | Page 5, Table S2 |
| 11 | Search software used, name and version, including special features used (eg. explosion) | Table S2 |
| 12 | Use of hand searching (eg. reference list of obtained articles) | Page 5, eMethods |
| 13 | List of citations located and those excluded, including justification | Fig. 1, eReferences |
| 14 | Method of addressing articles published in languages other than English | Page 5 |
| 15 | Method of handling abstracts and unpublished studies | eMethods |
| 16 | Description of any contact with authors | Page 7 |
| **Reporting of methods should include** | | |
| 17 | Description of relevance or appropriateness of studies assembled for assessing the hypothesis to be tested | Page 6 |
| 18 | Rationale for the selection and coding of data (eg. sound clinical principles or convenience) | Page 6, 7 |
| 19 | Documentation of how data were classified and coded (eg. multiple raters, blinding and interrater reliability) | Page 7, eMethods |
| 20 | Assessment of confounding (eg. comparability of cases and controls in studies where appropriate) | Page 7, 8, 9 |

Stroup DF, Berlin JA, Morton SC, et al. Meta-analysis of observational studies in epidemiology: a proposal for reporting. Meta-analysis Of Observational Studies in Epidemiology (MOOSE) group. JAMA. 2000;283(15):2008-12.

**Table S1** Meta-Analysis of Observational Studies in Epidemiology (MOOSE) statement Checklist (Continued)

| **Item No** | **Recommendation** | **Reported on Page No** |
| --- | --- | --- |
| **Reporting of methods should include (Continued)** | | |
| 21 | Assessment of study quality, including blinding of quality assessors; stratification or regression on possible predictors of study results | Page 7 |
| 22 | Assessment of heterogeneity | Page 7, 8 |
| 23 | Description of statistical methods (eg. complete description of fixed or random effects models, justification of whether the chosen models account for predictions of study results, dose-response models, or cumulative meta-analysis) in sufficient detail to be replicated | Page 7 |
| 24 | Provision of appropriate tables and graphics | Throughout tables/figures |
| **Reporting of results should include** | | |
| 25 | Graph summarizing individual study estimates and overall estimate | Throughout figures |
| 26 | Table giving descriptive information for each study included | Throughout tables |
| 27 | Results of sensitivity testing (eg. subgroup analysis) | Page 11, 12 |
| 28 | Indication of statistical uncertainty of findings | Page 12 |
| **Reporting of discussion should include** | | |
| 29 | Quantitative assessment of bias (eg. publication bias) | Page 15 |
| 30 | Justification for exclusion (eg. exclusion of non-English language citations) | Not applicable |
| 31 | Assessment of quality of included studies | Page 15 |
| **Reporting of conclusion should include** | | |
| 32 | Consideration of alternative explanations for observed results | Page 13, 14 |
| 33 | Generalization of the conclusions (eg. appropriate for the data presented and within the domain of the literature review) | Page 11, 16 |
| 34 | Guidelines for future research | Page 15, 16 |
| 35 | Disclosure of funding source | Page 24 |

Stroup DF, Berlin JA, Morton SC, et al. Meta-analysis of observational studies in epidemiology: a proposal for reporting. Meta-analysis Of Observational Studies in Epidemiology (MOOSE) group. JAMA. 2000;283(15):2008-12.
