# Supplementary material for: Use of serotonin reuptake inhibitor antidepressants and the risk of bleeding complications in patients on anticoagulant or antiplatelet agents: a systematic review and meta-analysis
Source: Ann Med. 2021 Dec 27;54(1):80–97. doi: 10.1080/07853890.2021.2017474 (PMC8725830; doi:10.1080/07853890.2021.2017474)
Supplement: Supplemental Material [file IANN_A_2017474_SM7515.zip › Supplementart files/Supplement_SRIs_Ann_Med.pdf]

Supplementary Online

Use of Serotonin Reuptake Inhibitor Antidepressants and the Risk of Bleeding Complications in Patients on Anticoagulant or Antiplatelet Agents: A Systematic Review and Meta-Analysis

Surapon Nochaiwong*; Chidchanok Ruengorn; Ratanaporn Awiphan; Chatree Chai-Adisaksopha; Apichat Tantraworasin; Chabaphai Phosuya; Penkarn Kanjanarat; Wilaiwan Chongruksut; Manish M. Sood; Kednapa Thavorn

***Correspondence:**
Surapon Nochaiwong, PharmD
Pharmacoepidemiology and Statistics Research Center (PESRC)
Department of Pharmaceutical Care, Faculty of Pharmacy, Chiang Mai University, Chiang Mai 50200, Thailand, Phone: 66899973365, Fax: 6653222741, Email: surapon.nochaiwong@gmail.com

Supplementary Online Content

**eTable 1**	Systematic Review Search Strategy	S3
**eTable 2**	The PICOTS Format: Study Inclusion/Exclusion Criteria	S22
**eTable 3**	Measurement and Definition of Bleeding Events of Included Studies	S23
**eTable 4**	Methods of Included Studies in the Meta-Analysis	S33
**eTable 5**	Characteristics of Study Participants Included in the Meta-Analysis	S45
**eTable 6**	Risk of Bias Assessment of Included Studies	S48
**eTable 7**	Quality of Evidence Synthesis and GRADE Evidence Profile of Outcomes	S53
**eTable 8**	Summary of Additional Secondary Outcomes	S59
**eTable 9**	Subgroup Analysis	S60
**eTable 10**	Sensitivity Analysis: Restricted the Analysis to Studies that Adjustment for Key Determinants	S64
**eTable 11**	Sensitivity Analysis: Restricted the Analysis to the Highest-Quality Study	S65
**eTable 12**	Sensitivity Analysis: Restricted the Analysis to Studies with the Directness of Effect Estimates	S66
**eTable 13**	Sensitivity Analysis: Removing Unpublished Studies	S67
**eTable 14**	Sensitivity Analysis: Outcomes After Removing Individuals Studies	S68
**eTable 15**	Sensitivity Analysis: Using Fixed-Effects Models	S70
**eTable 16**	Meta-Regression of Included Studies	S71
**eTable 17**	Meta-Analysis of Included Studies with Calibration for Publication Bias	S76
**eFigure 1**	The Funnel Plot of Included Studies in the Meta-Analysis	S77
**eReferences**		S81

eTable 1. Systematic Review Search Strategy

OVID: Medline (From Inception to November 26, 2020)		
**Search**	**Query**	**Items Found**
#1	exp antithrombotic/	172,488
#2	(antithrombotic drugs or antithrombotic agents).tw,kw,rn.	3,223
#3	exp anticoagulants/	223,369
#4	exp Vitamin K/ or thrombin/ or factor Xa/ or exp Blood coagulation factors/	462,332
#5	exp antithrombins/ or hirudin therapy/	23,551
#6	(anticoagul$ or antithromb$).tw,kw,rn.	157,560
#7	(Vitamin K antagonist$ or VKA or VKAs).tw,kw,rn.	6,571
#8	(NOAC$ or DOAC$).tw,kw,rn.	4,738
#9	(direct$ adj3 thrombin adj3 inhib$).tw,kw,rn.	2,753
#10	DTI$1.tw,kw,rn.	11,884
#11	exp Heparin/	64,865
#12	heparin$.tw,kw,rn.	104,980
#13	(UFH or LMWH or LMH).tw,kw,rn.	6,796
#14	((factor Xa or factor 10a or fXa or autoprothrombin c or thrombokinase) adj3 inhib$).tw,kw,rn	3,946
#15	(activated adj3 (factor X or factor 10) adj3 inhib$).tw,kw,rn	204
#16	(acenocoumarol$ or dicoumarol$ or ethyl biscoumacetate$ or phenprocoumon$ or warfarin$ or ancrod$ or citric acid$ or coumarin$ or chromonar$ or coumestro$ or esculi$ or ochratoxin$ or umbelliferone$ or dermatan sulfate$ or dextran$ or edetic acid$ or enoxaparin$ or gabexate$ or heparin$ or lmwh$ or nadroparin$ or pentosan sulfuric polyester$ or phenindione$ or protein c or protein s or tedelparin$).tw,kw,rn.	284,207
#17	(tinzaparin or parnaparin or dalteparin or reviparin or danaparoid or lomoparan or org 10172 or mesoglycan or polysaccharide sulphate$ or sp54 or sp-54 or md805 or md-805 or cy222 or cy-222 or cy216 or cy-216).tw,kw,rn.	3,366
#18	(Marevan or Fragmin$ or Fraxiparin$ or Klexane).tw,kw,rn.	619
#19	(argatroban or dabigatran or ximelagatran or melagatran or efegatran or flovagatran or inogatran or napsagatran or bivalirudin or lepirudin or hirudin$ or desirudin or desulfatohirudin or hirugen or hirulog or AZD0837 or bothrojaracin or odiparcil).tw,kw,rn.	13,161
#20	(xabans or antistasin or apixaban or betrixaban or du 176b or eribaxaban or fondaparinux or idraparinux or otamixaban or razaxaban or rivaroxaban or yagin or ym 150 or ym150 or LY517717).tw,kw,rn.	9,315
#21	exp platelet aggregation inhibitors/ or exp platelet glycoprotein gpiib-iiia complex/	128,120

#22	exp Phosphodiesterase inhibitors/	85,733
#23	(antiplatelet$ or anti-platelet$ or antiaggreg$ or anti-aggreg$ or (platelet$ adj3 inhibit$) or (thrombocyt$ adj3 inhibit$) or thienopyridine$).tw,kw,rn.	52,596
#24	(alprostadil$ or aspirin$ or acetylsalicylic acid or acetyl salicylic acid$ or acetyl?salicylic acid or epoprostenol$ or ketanserin$ or ketorolac tromethamine$ or milrinone$ or mopidamol$ or procainamide$ or thiophen$ or trapidil$ or picotamide$ or ligustrazine$ or levamisol$ or suloctidil$ or ozagrel$ or oky046 or oky-046 or defibrotide$ or cilostazol or satigrel or sarpolgrelate or kbt3022 or kbt-3022 or isbogrel or cv4151 or cv-4151 or ((glycoprotein iib$ or gp iib$) adj5 (antagonist$ or inhibitor$)) or GR144053 or GR-144053 or triflusal).tw,kw,rn.	137,698
#25	(Beraprost or Cicaprost or Cilostazol or Clopidogrel or Dipyridamole or Iloprost or Indobufen or Lepirudin or Pentosan Polysulfate or Pentoxifylline or Piracetam or Prostacyclin or Sulfinpyrazone or Sulphinpyrazone or Ticlopidine or Triflusal or Abciximab or Disintegrin or Echistatin or Eptifibatide or Lamifiban or Orbofiban or Roxifiban or Sibrafiban or Tirofiban or Xemilofiban or terutroban or picotamide or prasugrel).tw,kw,rn.	64,760
#26	(Dispril or Albyl$ or Ticlid$ or Persantin$ or Plavix or ReoPro or Integrilin$ or Aggrastat).tw,kw,rn.	1,177
#27	or/1-26	1,074,523
#28	exp serotonin uptake inhibitors/ or serotonin noradrenalin reuptake inhibitor/	42,580
#29	(((serotonin$ or serotonergic$) adj3 inhib$) or ((norepinehprine$ or noradrenaline$) adj3 inhibi$)).tw,kw,rn.	22,815
#30	(SSRI$ or SNRI$ or SRI$).tw,kw,rn.	24,965
#31	(citalopram or Celexa or dapoxetin$ or Priligy or desvenlafaxin$ or Pristiq or duloxetine$ or Cymbalta or escitalopram or Lexapro or fluoxetine$ or Prozac or Sarafem or Symbyax or fluvoxamine$ or Luvox or levomilnacipran or Fetzima or milnacipran or Savella or paroxetine$ or Paxil or Brisdelle or sertraline$ or Zoloft or venlafaxine$ or Effexor).tw,kw,rn.	37,209
#32	or/28-31	80,609
#33	27 and 32	4,782
#34	exp h?emorrhage/ or h?ematoma/ or bleeding/	332,092
#35	(h?emorrhag$ or h?ematoma$ or bleed$ or re-bleed$ or rebleed$).tw,kw.	480,653
#36	((brain$ or cerebr$ or cerebell$ or intracerebral or intracran$ or parenchymal or intraparenchymal or intraventricular or infratentorial or supratentorial or basal gangli$ or putaminal or putamen or posterior fossa or hemispher$ or subdural$ or epidural$ or subarachnoid$ or gastrointest$ or GI or stmach or gastri$ or gastroduo$ or peptic$ or retroperito$ or urogenit$ or genital or postpartum$ or $ocular or $conjunctival) adj5 (bleed$ or h?emorrhag$)).tw,kw.	120,622
#37	(h?ematemesis or mel?ena or h?ematochezia or h?emoptysis or epistaxis).tw,kw.	25,133
#38	(ICH$ or SAH$ or GIB$ or UGIB$ or LGIB$ or OGIB$).tw,kw.	94,988
#39	(($operative or $operation) adj5 (bleed$ or transfusion)).tw,kw.	7,086

#40	or/34-39	698,905
#41	33 and 40	287
#42	Randomized Controlled Trials as Topic/	138,297
#43	random allocation/	104,083
#44	Controlled Clinical Trials as Topic/	5,549
#45	control groups/	1,708
#46	clinical trials as topic/ or clinical trials, phase i as topic/ or clinical trials, phase ii as topic/ or clinical trials, phase iii as topic/ or clinical trials, phase iv as topic/	211,243
#47	Placebos/ or placebo effect/	39,169
#48	(placebo$ or sham).tw,kw.	305,130
#49	randomized controlled trial.pt.	517,852
#50	controlled clinical trial.pt.	93,944
#51	(clinical trial or clinical trial phase i or clinical trial phase ii or clinical trial phase iii or clinical trial phase iv).pt.	574,627
#52	(random$ or RCT or RCTs).tw,kw.	1,184,599
#53	(randomi?ed or randomly or RCT$1 or placebo$).tw,kw.	1,007,557
#54	((singl$ or doubl$ or trebl$ or tripl$) adj5 (mask$ or blind$ or dumm$)).tw,kw.	178,833
#55	(controlled adj5 (trial$ or stud$)).tw,kw.	395,234
#56	(clinical$ adj5 trial$).tw,kw.	418,738
#57	((control or treatment or experiment$ or intervention) adj5 (group$ or subject$ or patient$)).tw,kw.	1,548,389
#58	(quasi-random$ or quasi random$ or pseudo-random$ or pseudo random$).tw,kw.	5,929
#59	((control or experiment$ or conservative) adj5 (treatment or therapy or procedure or manage$)).tw,kw.	220,417
#60	trial.ti.	229,673
#61	(assign$ or allocat$).tw.	442,552
#62	or/42-61	3,635,641
#63	41 and 62	90
#64	(nRCT or nRCTs or non-RCT?).tw,kw.	970
#65	(control$ adj2 stud$3).tw,kw.	238,715
#66	control group/	1,708
#67	(control$ adj2 group$1).tw,kw.	518,426
#68	exp comparative study/	1,876,259

#69	((comparative or comparison) adj (study or studies)).tw,kw.	112,326
#70	exp cohort study/	2,058,703
#71	(cohort$ adj2 stud$3).tw,kw.	242,460
#72	exp case control study/	1,121,452
#73	((case-control$ or case-based or case-comparison) adj (study or studies)).tw,kw.	109,979
#74	or/64-73	4,397,295
#75	41 and 74	119
#76	63 or 75	157
#77	exp Adolescent/ not (exp Adult/ and Adolescent/)	614,231
#78	exp Child/ not (exp Adult/ and Child/)	1,263,155
#79	exp Infant/ not (exp Adult/ and Infant/)	923,317
#80	or/77-79	2,018,364
#81	76 not 80	153
#82	exp Animals/ not (exp Animals/ and Humans/)	4,759,541
#83	81 not 82	149
#84	(comment or editorial or interview or letter or news or newspaper article).pt.	2,139,330
#85	83 not 84	147

eTable 1. Systematic Review Search Strategy (Continued)

**OVID: Embase (From Inception to November 26, 2020)**		
**Search**	**Query**	**Items Found**
#1	exp antithrombotic/	647,313
#2	(antithrombotic drugs or antithrombotic agents).tw,kw,rn.	4,866
#3	exp anticoagulants/	647,313
#4	exp Vitamin K/ or thrombin/ or factor Xa/ or exp Blood coagulation factors/	263,750
#5	exp antithrombins/ or hirudin therapy/	65,411
#6	(anticoagul$ or antithromb$).tw,kw,rn.	187,467
#7	(Vitamin K antagonist$ or VKA or VKAs).tw,kw,rn.	11,615
#8	(NOAC$ or DOAC$).tw,kw,rn.	9,640
#9	(direct$ adj3 thrombin adj3 inhib$).tw,kw,rn.	4,417
#10	DTI$1.tw,kw,rn.	20,314
#11	exp Heparin/	136,302
#12	heparin$.tw,kw,rn.	182,088
#13	(UFH or LMWH or LMH).tw,kw,rn.	12,884
#14	((factor Xa or factor 10a or fXa or autoprothrombin c or thrombokinase) adj3 inhib$).tw,kw,rn	6,438
#15	(activated adj3 (factor X or factor 10) adj3 inhib$).tw,kw,rn	271
#16	(acenocoumarol$ or dicoumarol$ or ethyl biscoumacetate$ or phenprocoumon$ or warfarin$ or ancrod$ or citric acid$ or coumarin$ or chromonar$ or coumestro$ or esculi$ or ochratoxin$ or umbelliferone$ or dermatan sulfate$ or dextran$ or edetic acid$ or enoxaparin$ or gabexate$ or heparin$ or lmwh$ or nadroparin$ or pentosan sulfuric polyester$ or phenindione$ or protein c or protein s or tedelparin$).tw,kw,rn.	443,077
#17	(tinzaparin or parnaparin or dalteparin or reviparin or danaparoid or lomoparan or org 10172 or mesoglycan or polysaccharide sulphate$ or sp54 or sp-54 or md805 or md-805 or cy222 or cy-222 or cy216 or cy-216).tw,kw,rn.	6,608
#18	(Marevan or Fragmin$ or Fraxiparin$ or Klexane).tw,kw,rn.	3,229
#19	(argatroban or dabigatran or ximelagatran or melagatran or efegatran or flovagatran or inogatran or napsagatran or bivalirudin or lepirudin or hirudin$ or desirudin or desulfatohirudin or hirugen or hirulog or AZD0837 or bothrojaracin or odiparcil).tw,kw,rn.	27,696
#20	(xabans or antistasin or apixaban or betrixaban or du 176b or eribaxaban or fondaparinux or idraparinux or otamixaban or razaxaban or rivaroxaban or yagin or ym 150 or ym150 or LY517717).tw,kw,rn.	29,205
#21	exp platelet aggregation inhibitors/ or exp platelet glycoprotein gpiib-iiia complex/	345,456

#22	exp Phosphodiesterase inhibitors/	76,053
#23	(antiplatelet$ or anti-platelet$ or antiaggreg$ or anti-aggreg$ or (platelet$ adj3 inhibit$) or (thrombocyt$ adj3 inhibit$) or thienopyridine$).tw,kw,rn.	81,335
#24	(alprostadil$ or aspirin$ or acetylsalicylic acid or acetyl salicylic acid$ or acetyl?salicylic acid or epoprostenol$ or ketanserin$ or ketorolac tromethamine$ or milrinone$ or mopidamol$ or procainamide$ or thiophen$ or trapidil$ or picotamide$ or ligustrazine$ or levamisol$ or suloctidil$ or ozagrel$ or oky046 or oky-046 or defibrotide$ or cilostazol or satigrel or sarpolgrelate or kbt3022 or kbt-3022 or isbogrel or cv4151 or cv-4151 or ((glycoprotein iib$ or gp iib$) adj5 (antagonist$ or inhibitor$)) or GR144053 or GR-144053 or triflusal).tw,kw,rn.	278,046
#25	(Beraprost or Cicaprost or Cilostazol or Clopidogrel or Dipyridamole or Iloprost or Indobufen or Lepirudin or Pentosan Polysulfate or Pentoxifylline or Piracetam or Prostacyclin or Sulfinpyrazone or Sulphinpyrazone or Ticlopidine or Triflusal or Abciximab or Disintegrin or Echistatin or Eptifibatide or Lamifiban or Orbofiban or Roxifiban or Sibrafiban or Tirofiban or Xemilofiban or terutroban or picotamide or prasugrel).tw,kw,rn.	163,398
#26	(Dispril or Albyl$ or Ticlid$ or Persantin$ or Plavix or ReoPro or Integrilin$ or Aggrastat).tw,kw,rn.	7,704
#27	or/1-26	1,201,998
#28	exp serotonin uptake inhibitors/ or serotonin noradrenalin reuptake inhibitor/	261,287
#29	(((serotonin$ or serotonergic$) adj3 inhib$) or ((norepinehprine$ or noradrenaline$) adj3 inhibi$)).tw,kw,rn.	30,637
#30	(SSRI$ or SNRI$ or SRI$).tw,kw,rn.	37,677
#31	(citalopram or Celexa or dapoxetin$ or Priligy or desvenlafaxin$ or Pristiq or duloxetine$ or Cymbalta or escitalopram or Lexapro or fluoxetine$ or Prozac or Sarafem or Symbyax or fluvoxamine$ or Luvox or levomilnacipran or Fetzima or milnacipran or Savella or paroxetine$ or Paxil or Brisdelle or sertraline$ or Zoloft or venlafaxine$ or Effexor).tw,kw,rn.	107,181
#32	or/28-31	291,942
#33	27 and 32	26,250
#34	exp h?emorrhage/ or h?ematoma/ or bleeding/	901,277
#35	(h?emorrhag$ or h?ematoma$ or bleed$ or re-bleed$ or rebleed$).tw,kw.	663,461
#36	((brain$ or cerebr$ or cerebell$ or intracerebral or intracran$ or parenchymal or intraparenchymal or intraventricular or infratentorial or supratentorial or basal gangli$ or putaminal or putamen or posterior fossa or hemispher$ or subdural$ or epidural$ or subarachnoid$ or gastrointest$ or GI or stmach or gastri$ or gastroduo$ or peptic$ or retroperito$ or urogenit$ or genital or postpartum$ or $ocular or $conjunctival) adj5 (bleed$ or h?emorrhag$)).tw,kw.	176,313
#37	(h?ematemesis or mel?ena or h?ematochezia or h?emoptysis or epistaxis).tw,kw.	40,259
#38	(ICH$ or SAH$ or GIB$ or UGIB$ or LGIB$ or OGIB$).tw,kw.	127,505
#39	(($operative or $operation) adj5 (bleed$ or transfusion)).tw,kw.	12,354

#40	or/34-39	1,180,073
#41	33 and 40	3,599
#42	Randomized Controlled Trials as Topic/	126,056
#43	random allocation/	85,215
#44	Controlled Clinical Trials as Topic/	9,929
#45	control groups/	110,526
#46	clinical trials as topic/ or clinical trials, phase i as topic/ or clinical trials, phase ii as topic/ or clinical trials, phase iii as topic/ or clinical trials, phase iv as topic/	136,902
#47	Placebos/ or placebo effect/	293,552
#48	(placebo$ or sham).tw,kw.	428,917
#49	(random$ or RCT or RCTs).tw,kw.	1,612,733
#50	(randomi?ed or randomly or RCT$1 or placebo$).tw,kw.	1,415,717
#51	((singl$ or doubl$ or trebl$ or tripl$) adj5 (mask$ or blind$ or dumm$)).tw,kw.	242,841
#52	(controlled adj5 (trial$ or stud$)).tw,kw.	534,667
#53	(clinical$ adj5 trial$).tw,kw.	603,538
#54	((control or treatment or experiment$ or intervention) adj5 (group$ or subject$ or patient$)).tw,kw.	2,307,763
#55	(quasi-random$ or quasi random$ or pseudo-random$ or pseudo random$).tw,kw.	6,919
#56	((control or experiment$ or conservative) adj5 (treatment or therapy or procedure or manage$)).tw,kw.	298,810
#57	trial.ti.	309,590
#58	(assign$ or allocat$).tw.	554,882
#59	or/42-58	4,632,645
#60	41 and 59	947
#61	(nRCT or nRCTs or non-RCT?).tw,kw.	1,360
#62	(control$ adj2 stud$3).tw,kw.	319,997
#63	control group/	110,526
#64	(control$ adj2 group$1).tw,kw.	737,906
#65	exp comparative study/	1,361,719
#66	((comparative or comparison) adj (study or studies)).tw,kw.	127,823
#67	exp cohort study/	639,649
#68	(cohort$ adj2 stud$3).tw,kw.	366,089

#69	exp case control study/	182,583
#70	((case-control$ or case-based or case-comparison) adj (study or studies)).tw,kw.	144,358
#71	or/61-70	3,041,740
#72	41 and 71	452
#73	60 or 72	1,187
#74	exp Adolescent/ not (exp Adult/ and Adolescent/)	559,629
#75	exp Child/ not (exp Adult/ and Child/)	1,957,474
#76	exp Infant/ not (exp Adult/ and Infant/)	794,670
#77	or/74-76	2,188,829
#78	73 not 77	1,170
#79	exp Animals/ not (exp Animals/ and Humans/)	12,400,586
#80	78 not 79	682
#81	(comment or editorial or interview or letter or news or newspaper article).pt.	1,788,466
#82	80 not 81	679

eTable 1. Systematic Review Search Strategy (Continued)

**PubMed (From Inception to November 26, 2020)**		
**Search**	**Query**	**Items Found**
#1	((antithrombotic[Title/Abstract])) OR ((((antithrombotic drugs[MeSH Terms]) OR antithrombotic agents[MeSH Terms]) OR anticoagulants[MeSH Terms]) OR antiplatelet agents[MeSH Terms])	147,014
#2	((((((((((((((vitamin K antagonists) OR novel oral anticoagulants) OR direct oral anticoagulants) OR heparin) OR unfractionated heparin) OR low molecular weight heparin) OR direct thrombin inhibitors) OR factor xa inhibitors) OR hirudin therapy) OR VKA) OR NOAC) OR DOAC) OR UFH) OR LMWH) OR LMH	135,235
#3	((acenocoumarol) OR dicoumarol) OR ethyl biscoumacetate) OR phenprocoumon) OR warfarin) OR ancrod) OR citric acid) OR coumarin) OR chromonar) OR coumestrol) OR esculin) OR ochratoxin) OR umbelliferone) OR dermatan sulfate) OR dextran) OR edetic acid) OR enoxaparin) OR gabexate) OR heparin) OR nadroparin) OR pentosan sulfuric polyester) OR phenindione) OR tedelparin) OR tinzaparin) OR parnaparin) OR dalteparin) OR reviparin) OR org 10172) OR sp54) OR sp-54) OR md805) OR md-805) OR cy222) OR cy-222) OR cy216) OR cy-216) OR danaparoid) OR lomoparan) OR mesoglycan) OR polysaccharide sulphate) OR fondaparinux) OR idraparinux) OR marvan) OR fragmin) OR fraxiparin) OR clexane) OR apixaban) OR argatroban) OR betrixaban) OR eribaxaban) OR otamixaban) OR razaxaban) OR rivaroxaban) OR xabans) OR dabigatran) OR ximelagatran) OR melagatran) OR efegatran) OR flovagatran) OR inogatran) OR napsagatran) OR antistasin) OR bivalirudin) OR lepirudin) OR hirudin) OR desirudin) OR desulfatohirudin) OR hirugen) OR hirulog) OR AZD0837) OR bothrojaracin) OR odiparcil) OR du 176b) OR yagi) OR ym 150) OR ym150) OR LY517717	328,023
#4	(((((((antiplatelet) OR anti-platelet) OR platelet aggregation inhibitors) OR platelet glycoprotein gpiib-iiia complex) OR glycoprotein inhibitors) OR phosphodiesterase inhibitors) OR thienopyridine) OR P2Y12 receptor inhibitors	375,972
#5	((alprostadil) OR aspirin) OR acetylsalicylic acid) OR acetyl salicylic acid) OR acetyl?salicylic acid) OR ASA) OR epoprostenol) OR ketanserin) OR ketorolac) OR tromethamine) OR milrinone) OR mopidamol) OR procainamide) OR thiophen) OR trapidil) OR picotamide) OR ligustrazine) OR levamisole) OR suloctidil) OR ozagrel) OR oky046) OR oky-046) OR defibrotide) OR cilostazol) OR satigrel) OR sarpogrelate) OR kbt3022) OR kbt-3022) OR isbogrel) OR cv4151) OR cv-4151) OR GR144053) OR GR-144053) OR triflusal) OR beraprost) OR cicaprost) OR clopidogrel) OR dipyridamole) OR iloprost) OR indobufen) OR lepirudin) OR pentosan polysulfate) OR pentoxifylline) OR piracetam) OR prostacyclin) OR sulfinpyrazone) OR sulphinpyrazone) OR ticlopidine) OR abciximab) OR disintegrin) OR echistatin) OR eptifibatide) OR lamifiban) OR orbofiban) OR roxifiban) OR sibrafiban) OR tirofiban) OR xemilofiban) OR terutroban) OR picotamide) OR prasugrel) OR disopril) OR alkyl) OR ticlid) OR persantin) OR plavix) OR reopro) OR integrilin) OR aggrastat	504,276
#6	#1 OR #2 OR #3 OR #4 OR #5	1,123,573
#7	(((((((((((((((serotonin uptake inhibitors[MeSH Terms]) OR selective serotonin reuptake inhibitors) OR (serotonin antagonist and reuptake inhibitor)) OR serotonergic serotonin-norepinephrine reuptake inhibitors) OR serotoninergic serotonin-norepinephrine	85,340

	reuptake inhibitors) OR selective serotonin reuptake inhibitor antidepressants) OR (serotonin and norepinephrine reuptake inhibitors)) OR (serotonin and noradrenaline reuptake inhibitors)) OR serotonin-norepinephrine reuptake inhibitors) OR serotonin reuptake inhibitors) OR serotonin releasing agents) OR selective specific reuptake inhibitors) OR serotonin-specific reuptake inhibitors) OR SSRI) OR SNRI) OR SRI	
#8	(((((((((((((((((((((((((citalopram) OR Celexa) OR dapoxetine) OR Priligy) OR desvenlafaxine) OR Pristiq) OR duloxetine) OR Cymbalta) OR escitalopram) OR Lexapro) OR fluoxetine) OR Prozac) OR Sarafem) OR Symbyax) OR fluvoxamine) OR Luvox) OR levomilnacipran) OR Fetzima) OR milnacipran) OR Savella) OR paroxetine) OR Paxil) OR Brisdelle) OR sertraline) OR Zoloft) OR venlafaxine) OR Effexor	44,197
#9	#7 OR #8	96,019
#10	#6 AND #9	6,194
#11	((h?emorrhage) OR h?ematoma) OR bleed) OR bleeding) OR re-bleed) OR rebleed) OR blood loss) OR systemic bleeding) OR abnormal bleeding) OR h?emorrhagic bleeding) OR h?emorrhagic complications) OR major bleeding) OR minor bleeding) OR h?emorrhagic stroke) OR intracranial h?emorrhage) OR intracranial bleeding) OR intracerebral h?emorrhage) OR intraparenchymal h?emorrhage) OR subdural h?emorrhage) OR epidermal h?emorrhage) OR subarachnoid h?emorrhage) OR brain h?emorrhage) OR brain bleeding) OR gastrointestinal bleeding) OR gi bleeding) OR retroperitoneal bleeding) OR urogenital bleeding) OR genital bleeding) OR ocular bleeding) OR intraocular bleeding) OR postpartum h?emorrhage) OR h?ematemesis) OR melena) OR h?ematochezia) OR h?emoptysis) OR epistaxis) OR ICH) OR SAH) OR GIB) OR ogib) OR ugib) OR lgib) OR perioperative blood loss) OR postoperative bleeding) OR blood transfusion	809,952
#12	#10 AND #11	360
#13	(((((((Case Reports[Publication Type]) OR Comment[Publication Type]) OR Editorial[Publication Type]) OR Guideline[Publication Type]) OR Letter[Publication Type]) OR News[Publication Type]) OR Newspaper Article[Publication Type]) OR Review[Publication Type]	6,624,763
#14	#12 NOT #13	228
#15	Filters: Humans	173

eTable 1. Systematic Review Search Strategy (Continued)

OVID: PsycINFO (From Inception to November 26, 2020)		
**Search**	**Query**	**Items Found**
#1	(antithrombotic or antithrombotic drugs or antithrombotic agents).mp.	255
#2	(Vitamin K or thrombin or factor Xa or blood coagulation factors or antithrombins or hirudin therapy or heparin$ or UFH or LMWH or LMH).mp.	1,241
#3	(anticoagul$ or antithromb$ or vitamin K antagonist$ or VKA$ or NOAC$ or DOAC$ or DTI$).mp.	5,825
#4	(direct$ adj3 thrombin adj3 inhib$).mp.	43
#5	((factor Xa or factor 10a or fXa or autoprothrombin c or thrombokinase) adj3 inhib$).mp.	32
#6	(acenocoumarol$ or dicoumarol$ or ethyl biscoumacetate$ or phenprocoumon$ or warfarin$ or ancrod$ or citric acid$ or coumarin$ or chromonar$ or coumestro$ or esculi$ or ochratoxin$ or umbelliferone$ or dermatan sulfate$ or dextran$ or edetic acid$ or enoxaparin$ or gabexate$ or heparin$ or lmwh$ or nadroparin$ or pentosan sulfuric polyester$ or phenindione$ or protein c or protein s or tedelparin$ or tinzaparin or parnaparin or dalteparin or reviparin or danaparoid or lomoparan or org 10172 or mesoglycan or polysaccharide sulphate$ or sp54 or sp-54 or md805 or md-805 or cy222 or cy-222 or cy216 or cy-216 or Marevan or Fragmin$ or Fraxiparin$ or Klexane or argatroban or dabigatran or ximelagatran or melagatran or efegatran or flovagatran or inogatran or napsagatran or bivalirudin or lepirudin or hirudin$ or desirudin or desulfatohirudin or hirugen or hirulog or AZD0837 or bothrojaracin or odiparcil or xabans or antistasin or apixaban or betrixaban or du 176b or eribaxaban or fondaparinux or idraparinux or otamixaban or razaxaban or rivaroxaban or yagin or ym 150 or ym150 or LY517717).mp.	3,264
#7	(platelet aggregation inhibitors or platelet glycoprotein gpiib-iiia complex or phosphodiesterase inhibitors).mp.	1,098
#8	(antiplatelet$ or anti-platelet$ or antiaggreg$ or anti-aggreg$ or (platelet$ adj3 inhibit$) or (thrombocyt$ adj3 inhibit$) or thienopyridine$).mp.	867
#9	((glycoprotein iib$ or gp iib$) adj5 (antagonist$ or inhibitor$)).mp.	5
#10	(alprostadil$ or aspirin$ or acetylsalicylic acid or acetyl salicylic acid$ or acetyl?salicylic acid or epoprostenol$ or ketanserin$ or ketorolac tromethamine$ or milrinone$ or mopidamol$ or procainamide$ or thiophen$ or trapidil$ or picotamide$ or ligustrazine$ or levamisol$ or suloctidil$ or ozagrel$ or oky046 or oky-046 or defibrotide$ or cilostazol or satigrel or sarpolgrelate or kbt3022 or kbt-3022 or isbogrel or cv4151 or cv-4151 or GR144053 or GR-144053 or triflusal or beraprost or cicaprost or cilostazol or clopidogrel or dipyridamole or iloprost or indobufen or lepirudin or pentosan polysulfate or pentoxifylline or piracetam or prostacyclin or sulfinpyrazone or sulphinpyrazone or ticlopidine or triflusal or abciximab or disintegrin or echistatin or eptifibatide or lamifiban or orbofiban or roxifiban or sibrafiban or tirofiban or xemilofiban or terutroban or picotamide or prasugrel or Dispril or Albyl$ or Ticlid$ or Persantin$ or Plavix or ReoPro or Integrilin$ or Aggrastat).mp.	6,779
#11	or/1-10	16,447
#12	(serotonin uptake inhibitors or serotonin noradrenalin reuptake inhibitor or SSRI$ or SNRI$ or SRI$).mp	15,264

#13	(((serotonin$ or serotonergic$) adj3 inhib$) or ((norepinehprine$ or noradrenaline$) adj3 inhibi$)).mp.	12,712
#14	(citalopram or Celexa or dapoxetin$ or Priligy or desvenlafaxin$ or Pristiq or duloxetine$ or Cymbalta or escitalopram or Lexapro or fluoxetine$ or Prozac or Sarafem or Symbyax or fluvoxamine$ or Luvox or levomilnacipran or Fetzima or milnacipran or Savella or paroxetine$ or Paxil or Brisdelle or sertraline$ or Zoloft or venlafaxine$ or Effexor).mp.	19,095
#15	or/12-14	30,337
#16	11 and 15	875
#17	(h?emorrhag$ or h?ematoma$ or bleed$ or re-bleed$ or rebleed$).mp.	11,022
#18	((brain$ or cerebr$ or cerebell$ or intracerebral or intracran$ or parenchymal or intraparenchymal or intraventricular or infratentorial or supratentorial or basal gangli$ or putaminal or putamen or posterior fossa or hemispher$ or subdural$ or epidural$ or subarachnoid$ or gastrointest$ or GI or stmach or gastri$ or gastroduo$ or peptic$ or retroperito$ or urogenit$ or genital or postpartum$ or $ocular or $conjunctival) adj5 (bleed$ or h?emorrhag$)).mp	6,082
#19	(h?ematemesis or mel?ena or h?ematochezia or h?emoptysis or epistaxis or ICH$ or SAH$ or GIB$ or UGIB$ or LGIB$ or OGIB$).mp.	14,892
#20	(($operative or $operation) adj5 (bleed$ or transfusion)).mp.	24
#21	or/11-20	68,691
#22	16 and 21	875
#23	limit 22 to human	658
#24	limit 23 to "300 adulthood <age 18 yrs and older>"	491

eTable 1. Systematic Review Search Strategy (Continued)

**Cochrane Library (From Inception to November 26, 2020)**		
**Search**	**Query**	**Items Found**
#1	antithrombotic OR "antithrombotic drugs" OR "antithrombotic agents" OR anticoagulants OR "antiplatelet agents"	9,073
#2	"vitamin K antagonists" OR "novel oral anticoagulants" OR "direct oral anticoagulants" OR heparin OR "unfractionated heparin" OR "low molecular weight heparin" OR "direct thrombin inhibitors" OR "factor xa inhibitors" OR "hirudin therapy" OR VKA OR NOAC OR DOAC OR UFH OR LMWH OR LMH	13,475
#3	antiplatelet OR anti-platelet OR "platelet aggregation inhibitors" OR "platelet glycoprotein gpiib-iiia complex" OR "glycoprotein inhibitors" OR "phosphodiesterase inhibitors" OR thienopyridine OR "P2Y12 receptor inhibitors"	10,193
#4	#1 OR #2 OR #3	26,394
#5	serotonin uptake inhibitors OR selective serotonin reuptake inhibitors OR serotonin antagonist and reuptake inhibitor OR serotonergic serotonin-norepinephrine reuptake inhibitors OR serotoninergic serotonin-norepinephrine reuptake inhibitors OR selective serotonin reuptake inhibitor antidepressants OR serotonin and norepinephrine reuptake inhibitors OR serotonin and noradrenaline reuptake inhibitors OR serotonin-norepinephrine reuptake inhibitors OR serotonin reuptake inhibitors OR serotonin releasing agents OR selective specific reuptake inhibitors OR serotonin-specific reuptake inhibitors OR SSRI OR SNRI OR SRI	7,884
#6	citalopram OR Celexa OR dapoxetine OR Priligy OR desvenlafaxine OR Pristiq OR duloxetine OR Cymbalta OR escitalopram OR Lexapro OR fluoxetine OR Prozac OR Sarafem OR Symbyax OR fluvoxamine OR Luvox OR levomilnacipran OR Fetzima OR milnacipran OR Savella OR paroxetine OR Paxil OR Brisdelle OR sertraline OR Zoloft OR venlafaxine OR Effexor	13,827
#7	#5 OR #6	17,452
#8	#4 AND #7	116
#9	h?emorrhage OR h?ematoma OR bleed OR bleeding OR re-bleed OR rebleed OR blood loss OR systemic bleeding OR abnormal bleeding OR h?emorrhagic bleeding OR h?emorrhagic complications OR major bleeding OR minor bleeding OR h?emorrhagic stroke OR intracranial h?emorrhage OR intracranial bleeding OR intracerebral h?emorrhage OR intraparenchymal h?emorrhage OR subdural h?emorrhage OR epidermal h?emorrhage OR subarachnoid h?emorrhage OR brain h?emorrhage OR brain bleeding OR gastrointestinal bleeding OR gi bleeding OR retroperitoneal bleeding OR urogenital bleeding OR genital bleeding OR ocular bleeding OR intraocular bleeding OR postpartum h?emorrhage OR h?ematemesis OR melena OR h?ematochezia OR h?emoptysis OR epistaxis OR ICH OR SAH OR GIB OR ogib OR ugib OR lgib OR perioperative blood loss OR postoperative bleeding OR blood transfusion	102,363
#10	#8 AND #9	68

eTable 1. Systematic Review Search Strategy (Continued)

Web of Science (From Inception to November 26, 2020)		
**Search**	**Query**	**Items Found**
#1	TS=(((((antithrombotic) OR antithrombotic drugs) OR antithrombotic agents) OR anticoagulants) OR antiplatelet agents)	65,720
#2	TS=(((((((((((((((vitamin K antagonists) OR novel oral anticoagulants) OR direct oral anticoagulants) OR heparin) OR unfractionated heparin) OR low molecular weight heparin) OR direct thrombin inhibitors) OR factor xa inhibitors) OR hirudin therapy) OR VKA) OR NOAC) OR DOAC) OR UFH) OR LMWH) OR LMH)	70,111
#3	TS=(((acenocoumarol) OR dicoumarol) OR ethyl biscoumacetate) OR phenprocoumon) OR warfarin) OR ancrod) OR citric acid) OR coumarin) OR chromonar) OR coumestrol) OR esculin) OR ochratoxin) OR umbelliferone) OR dermatan sulfate) OR dextran) OR edetic acid) OR enoxaparin) OR gabexate) OR heparin) OR nadroparin) OR pentosan sulfuric polyester) OR phenindione) OR tedelparin) OR tinzaparin) OR parnaparin) OR dalteparin) OR reviparin) OR org 10172) OR sp54) OR sp-54) OR md805) OR md-805) OR cy222) OR cy-222) OR cy216) OR cy-216) OR danaparoid) OR lomoparan) OR mesoglycan) OR polysaccharide sulphate) OR fondaparinux) OR idraparinux) OR marvan) OR fragmin) OR fraxiparin) OR clexane) OR apixaban) OR argatroban) OR betrixaban) OR eribaxaban) OR otamixaban) OR razaxaban) OR rivaroxaban) OR xabans) OR dabigatran) OR ximelagatran) OR melagatran) OR efegatran) OR flovagatran) OR inogatran) OR napsagatran) OR antistasin) OR bivalirudin) OR lepirudin) OR hirudin) OR desirudin) OR desulfatohirudin) OR hirugen) OR hirulog) OR AZD0837) OR bothrojaracin) OR odiparcil) OR du 176b) OR yagi) OR ym 150) OR ym150) OR LY517717)	180,017
#4	TS=((((((((antiplatelet) OR anti-platelet) OR platelet aggregation inhibitors) OR platelet glycoprotein gpiib-iiia complex) OR glycoprotein inhibitors) OR phosphodiesterase inhibitors) OR thienopyridine) OR P2Y12 receptor inhibitors)	68,734
#5	TS=(((alprostadil) OR aspirin) OR acetylsalicylic acid) OR acetyl salicylic acid) OR acetyl?salicylic acid) OR ASA) OR epoprostenol) OR ketanserin) OR ketorolac) OR tromethamine) OR milrinone) OR mopidamol) OR procainamide) OR thiophen) OR trapidil) OR picotamide) OR ligustrazine) OR levamisole) OR suloctidil) OR ozagrel) OR oky046) OR oky-046) OR defibrotide) OR cilostazol) OR satigrel) OR sarpogrelate) OR kbt3022) OR kbt-3022) OR isbogrel) OR cv4151) OR cv-4151) OR GR144053) OR GR-144053) OR triflusal) OR beraprost) OR cicaprost) OR clopidogrel) OR dipyridamole) OR iloprost) OR indobufen) OR lepirudin) OR pentosan polysulfate) OR pentoxifylline) OR piracetam) OR prostacyclin) OR sulfinpyrazone) OR sulphinpyrazone) OR ticlopidine) OR abciximab) OR disintegrin) OR echistatin) OR eptifibatide) OR lamifiban) OR orbofiban) OR roxifiban) OR sibrafiban) OR tirofiban) OR xemilofiban) OR terutroban) OR picotamide) OR prasugrel) OR disopril) OR alkyl) OR ticlid) OR persantin) OR plavix) OR reopro) OR integrilin) OR aggrastat)	215,307
#6	#1 OR #2 OR #3 OR #4 OR #5	457,967
#7	TS=((((((((((((((((serotonin uptake inhibitors) OR selective serotonin reuptake inhibitors) OR (serotonin antagonist and reuptake inhibitor)) OR serotonergic serotonin-norepinephrine reuptake inhibitors) OR serotoninergic serotonin-norepinephrine reuptake inhibitors) OR selective serotonin reuptake inhibitor antidepressants) OR (serotonin and norepinephrine reuptake inhibitors)) OR	39,893

	(serotonin and noradrenaline reuptake inhibitors)) OR serotonin-norepinephrine reuptake inhibitors) OR serotonin reuptake inhibitors) OR serotonin releasing agents) OR selective specific reuptake inhibitors) OR serotonin-specific reuptake inhibitors) OR SSRI) OR SNRI) OR SRI)	
#8	TS=(((((((((((((((((((((((((((citalopram) OR Celexa) OR dapoxetine) OR Priligy) OR desvenlafaxine) OR Pristiq) OR duloxetine) OR Cymbalta) OR escitalopram) OR Lexapro) OR fluoxetine) OR Prozac) OR Sarafem) OR Symbyax) OR fluvoxamine) OR Luvox) OR levomilnacipran) OR Fetzima) OR milnacipran) OR Savella) OR paroxetine) OR Paxil) OR Brisdelle) OR sertraline) OR Zoloft) OR venlafaxine) OR Effexor)	35,467
#9	#7 OR #8	63,857
#10	#6 AND #9	1,606
#11	TS=(((h?emorrhage) OR h?ematoma) OR bleed) OR bleeding) OR re-bleed) OR rebleed) OR blood loss) OR systemic bleeding) OR abnormal bleeding) OR h?emorrhagic bleeding) OR h?emorrhagic complications) OR major bleeding) OR minor bleeding) OR h?emorrhagic stroke) OR intracranial h?emorrhage) OR intracranial bleeding) OR intracerebral h?emorrhage) OR intraparenchymal h?emorrhage) OR subdural h?emorrhage) OR epidermal h?emorrhage) OR subarachnoid h?emorrhage) OR brain h?emorrhage) OR brain bleeding) OR gastrointestinal bleeding) OR gi bleeding) OR retroperitoneal bleeding) OR urogenital bleeding) OR genital bleeding) OR ocular bleeding) OR intraocular bleeding) OR postpartum h?emorrhage) OR h?ematemesis) OR melena) OR h?ematochezia) OR h?emoptysis) OR epistaxis) OR ICH) OR SAH) OR GIB) OR ogib) OR ugib) OR lgib) OR perioperative blood loss) OR postoperative bleeding) OR blood transfusion)	318,300
#12	#10 AND #11	260
#13	Refined by: DOCUMENT TYPES: (ARTICLE OR MEETING ABSTRACT OR PROCEEDINGS PAPER OR EARLY ACCESS)	199

eTable 1. Systematic Review Search Strategy (Continued)

Scopus (From Inception to November 26, 2020)		
**Search**	**Query**	**Items Found**
#1	TITLE-ABS-KEY (antithrombotic OR "antithrombotic drugs" OR "antithrombotic agents" OR anticoagulants OR "antiplatelet agents")	197,304
#2	TITLE-ABS-KEY ("vitamin k antagonists" OR "novel oral anticoagulants" OR "direct oral anticoagulants" OR heparin OR "unfractionated heparin" OR "low molecular weight heparin" OR "direct thrombin inhibitors" OR "factor xa inhibitors" OR "hirudin therapy" OR vka OR noac OR doac OR ufh OR lmwh OR lmh)	206,332
#3	TITLE-ABS-KEY (acenocoumarol OR dicoumarol OR "ethyl biscoumacetate" OR phenprocoumon OR warfarin OR ancrod OR "citric acid" OR coumarin OR chromonar OR coumestrol OR esculin OR ochratoxin OR umbelliferone OR "dermatan sulfate" OR dextran OR "edetic acid" OR enoxaparin OR gabexate OR heparin OR nadroparin OR "pentosane sulfuric polyester" OR phenindione OR tedelparin OR tinzaparin OR parnaparin OR dalteparin OR reviparin OR "org 10172" OR sp54 OR sp-54 OR md805 OR md-805 OR cy222 OR cy-222 OR cy216 OR cy-216 OR danaparoid OR lomoparan OR mesoglycan OR "polysaccharide sulphate" OR fondaparinux OR idraparinux OR marvan OR fragmin OR fraxiparin OR clexane OR apixaban OR argatroban OR betrixaban OR eribaxaban OR otamixaban OR razaxaban OR rivaroxaban OR xabans OR dabigatran OR ximelagatran OR melagatran OR efegatran OR flovagatran OR inogatran OR napsagatran OR antistasin OR bivalirudin OR lepirudin OR hirudin OR desirudin OR desulfatohirudin OR hirugen OR hirulog OR azd0837 OR bothrojaracin OR odiparcil OR "du 176b" OR yagi OR ym-150 OR ym150 OR ly517717)	521,317
#4	TITLE-ABS-KEY (antiplatelet OR anti-platelet OR "platelet aggregation inhibitors" OR "platelet glycoprotein gpiib-iiia complex" OR "glycoprotein inhibitors" OR "phosphodiesterase inhibitors" OR thienopyridine OR "p2y12 receptor inhibitors")	84,112
#5	TITLE-ABS-KEY (alprostadil OR aspirin OR "acetylsalicylic acid" OR "acetyl salicylic acid" OR "acetyl?salicylic acid" OR asa OR epoprostenol OR ketanserin OR ketorolac OR tromethamine OR milrinone OR mopidamol OR procainamide OR thiophen OR trapidil OR picotamide OR ligustrazine OR levamisole OR suloctidil OR ozagrel OR oky046 OR oky-046 OR defibrotide OR cilostazol OR satigrel OR sarpogrelate OR kbt3022 OR kbt-3022 OR isbogrel OR cv4151 OR cv-4151 OR gr144053 OR gr-144053 OR triflusal OR beraprost OR cicaprost OR clopidogrel OR dipyridamole OR iloprost OR indobufen OR lepirudin OR "pentosan polysulfate" OR pentoxifylline OR piracetam OR prostacyclin OR sulfinpyrazone OR sulphinpyrazone OR ticlopidine OR abciximab OR disintegrin OR echistatin OR eptifibatide OR lamifiban OR orbofiban OR roxifiban OR sibrafiban OR tirofiban OR xemilofiban OR terutroban OR picotamide OR prasugrel OR disopril OR alkyl OR ticlid OR persantin OR plavix OR reopro OR integrilin OR aggrastat)	582,448
#6	#1 OR #2 OR #3 OR #4 OR #5	1,141,693
#7	TITLE-ABS-KEY ("serotonin uptake inhibitors" OR "selective serotonin reuptake inhibitors" OR "serotonin antagonist and reuptake inhibitor" OR "serotonergic serotonin-norepinephrine reuptake inhibitors" OR "serotoninergic serotonin-norepinephrine reuptake inhibitors" OR "selective serotonin reuptake inhibitor antidepressants" OR "serotonin and norepinephrine reuptake	90,671

	inhibitors" OR "serotonin and noradrenaline reuptake inhibitors" OR "serotonin-norepinephrine reuptake inhibitors" OR "serotonin reuptake inhibitors" OR "serotonin releasing agents" OR "selective specific reuptake inhibitors" OR "serotonin-specific reuptake inhibitors" OR ssri OR snri OR sri)	
#8	TITLE-ABS-KEY (citalopram OR celexa OR dapoxetine OR priligy OR desvenlafaxine OR pristiq OR duloxetine OR cymbalta OR escitalopram OR lexapro OR fluoxetine OR prozac OR sarafem OR symbyax OR fluvoxamine OR luvox OR levomilnacipran OR fetzima OR milnacipran OR savella OR paroxetine OR paxil OR brisdelle OR sertraline OR zoloft OR venlafaxine OR effexor)	96,255
#9	#7 OR #8	153,215
#10	#6 AND #9	10,904
#11	TITLE-ABS-KEY (h?emorrhage OR hematoma OR bleed OR bleeding OR re-bleed OR rebleed OR "blood loss" OR "systemic bleeding" OR "abnormal bleeding" OR "hemorrhagic bleeding" OR "hemorrhagic complications" OR "major bleeding" OR "minor bleeding" OR "h?emorrhagic stroke" OR "intracranial h?emorrhage" OR "intracranial bleeding" OR "intracerebral h?emorrhage" OR "intraparenchymal h?emorrhage" OR "subdural h?emorrhage" OR "epidermal h?emorrhage" OR "subarachnoid h?emorrhage" OR "brain h?emorrhage" OR "brain bleeding" OR "gastrointestinal bleeding" OR "gi bleeding" OR "retroperitoneal bleeding" OR "urogenital bleeding" OR "genital bleeding" OR "ocular bleeding" OR "intraocular bleeding" OR "postpartum h?emorrhage" OR h?ematemesis OR melena OR h?ematochezia OR h?emoptysis OR epistaxis OR ich OR sah OR gib OR ogib OR ugib OR lgib OR "perioperative blood loss" OR "postoperative bleeding" OR "blood transfusion")	759,275
#12	#10 AND #11	1,321
#13	(LIMIT-TO (DOCTYPE , "ar") OR LIMIT-TO (DOCTYPE , "cp")) AND (LIMIT-TO (SRCTYPE , "j"))	657

eTable 1. Systematic Review Search Strategy (Continued)

CINAHL (From Inception to November 26, 2020)		
**Search**	**Query**	**Items Found**
#1	AB ((((antithrombotic) OR antithrombotic drugs) OR antithrombotic agents) OR anticoagulants) OR antiplatelet agents	21,108
#2	AB ((((((((((((((vitamin K antagonists) OR novel oral anticoagulants) OR direct oral anticoagulants) OR heparin) OR unfractionated heparin) OR low molecular weight heparin) OR direct thrombin inhibitors) OR factor xa inhibitors) OR hirudin therapy) OR VKA) OR NOAC) OR DOAC) OR UFH) OR LMWH) OR LMH	10,182
#3	AB ((acenocoumarol) OR dicoumarol) OR ethyl biscoumacetate) OR phenprocoumon) OR warfarin) OR ancrod) OR citric acid) OR coumarin) OR chromonar) OR coumestrol) OR esculin) OR ochratoxin) OR umbelliferone) OR dermatan sulfate) OR dextran) OR edetic acid) OR enoxaparin) OR gabexate) OR heparin) OR nadroparin) OR pentosan sulfuric polyester) OR phenindione) OR tedelparin) OR tinzaparin) OR parnaparin) OR dalteparin) OR reviparin) OR org 10172) OR sp54) OR sp-54) OR md805) OR md-805) OR cy222) OR cy-222) OR cy216) OR cy-216) OR danaparoid) OR lomoparan) OR mesoglycan) OR polysaccharide sulphate) OR fondaparinux) OR idraparinux) OR marvan) OR fragmin) OR fraxiparin) OR clexane) OR apixaban) OR argatroban) OR betrixaban) OR eribaxaban) OR otamixaban) OR razaxaban) OR rivaroxaban) OR xabans) OR dabigatran) OR ximelagatran) OR melagatran) OR efegatran) OR flovagatran) OR inogatran) OR napsagatran) OR antistasin) OR bivalirudin) OR lepirudin) OR hirudin) OR desirudin) OR desulfatohirudin) OR hirugen) OR hirulog) OR AZD0837) OR bothrojaracin) OR odiparcil) OR du 176b) OR yagi) OR ym 150) OR ym150) OR LY517717	16,991
#4	AB (((((((antiplatelet) OR anti-platelet) OR platelet aggregation inhibitors) OR platelet glycoprotein gpiib-iiia complex) OR glycoprotein inhibitors) OR phosphodiesterase inhibitors) OR thienopyridine) OR P2Y12 receptor inhibitors	8,815
#5	AB ((alprostadil) OR aspirin) OR acetylsalicylic acid) OR acetyl salicylic acid) OR acetyl?salicylic acid) OR ASA) OR epoprostenol) OR ketanserin) OR ketorolac) OR tromethamine) OR milrinone) OR mopidamol) OR procainamide) OR thiophen) OR trapidil) OR picotamide) OR ligustrazine) OR levamisole) OR suloctidil) OR ozagrel) OR oky046) OR oky-046) OR defibrotide) OR cilostazol) OR satigrel) OR sarpogrelate) OR kbt3022) OR kbt-3022) OR isbogrel) OR cv4151) OR cv-4151) OR GR144053) OR GR-144053) OR triflusal) OR beraprost) OR cicaprost) OR clopidogrel) OR dipyridamole) OR iloprost) OR indobufen) OR lepirudin) OR pentosan polysulfate) OR pentoxifylline) OR piracetam) OR prostacyclin) OR sulfinpyrazone) OR sulphinpyrazone) OR ticlopidine) OR abciximab) OR disintegrin) OR echistatin) OR eptifibatide) OR lamifiban) OR orbofiban) OR roxifiban) OR sibrafiban) OR tirofiban) OR xemilofiban) OR terutroban) OR picotamide) OR prasugrel) OR disopril) OR alkyl) OR ticlid) OR persantin) OR plavix) OR reopro) OR integrilin) OR aggrastat	20,020
#6	S1 OR S2 OR S3 OR S4 OR S5	47,270
#7	AB (((((((((((((((serotonin uptake inhibitors) OR selective serotonin reuptake inhibitors) OR (serotonin antagonist and reuptake inhibitor)) OR serotonergic serotonin-norepinephrine reuptake inhibitors) OR serotoninergic serotonin-norepinephrine reuptake inhibitors) OR selective serotonin reuptake inhibitor antidepressants) OR (serotonin and norepinephrine reuptake inhibitors)) OR	7,217

	(serotonin and noradrenaline reuptake inhibitors)) OR serotonin-norepinephrine reuptake inhibitors) OR serotonin reuptake inhibitors) OR serotonin releasing agents) OR selective specific reuptake inhibitors) OR serotonin-specific reuptake inhibitors) OR SSRI) OR SNRI) OR SRI	
#8	AB ((((((((((((((((((((((((((citalopram) OR Celexa) OR dapoxetine) OR Priligy) OR desvenlafaxine) OR Pristiq) OR duloxetine) OR Cymbalta) OR escitalopram) OR Lexapro) OR fluoxetine) OR Prozac) OR Sarafem) OR Symbyax) OR fluvoxamine) OR Luvox) OR levomilnacipran) OR Fetzima) OR milnacipran) OR Savella) OR paroxetine) OR Paxil) OR Brisdelle) OR sertraline) OR Zoloft) OR venlafaxine) OR Effexor	5,134
#9	S7 OR S8	10,924
#10	S6 AND S9	243
#11	AB ((h?emorrhage) OR h?ematoma) OR bleed) OR bleeding) OR re-bleed) OR rebleed) OR blood loss) OR systemic bleeding) OR abnormal bleeding) OR h?emorrhagic bleeding) OR h?emorrhagic complications) OR major bleeding) OR minor bleeding) OR h?emorrhagic stroke) OR intracranial h?emorrhage) OR intracranial bleeding) OR intracerebral h?emorrhage) OR intraparenchymal h?emorrhage) OR subdural h?emorrhage) OR epidermal h?emorrhage) OR subarachnoid h?emorrhage) OR brain h?emorrhage) OR brain bleeding) OR gastrointestinal bleeding) OR gi bleeding) OR retroperitoneal bleeding) OR urogenital bleeding) OR genital bleeding) OR ocular bleeding) OR intraocular bleeding) OR postpartum h?emorrhage) OR h?ematemesis) OR melena) OR h?ematochezia) OR h?emoptysis) OR epistaxis) OR ICH) OR SAH) OR GIB) OR ogib) OR ugib) OR lgib) OR perioperative blood loss) OR postoperative bleeding) OR blood transfusion	86,461
#12	S10 AND S11	74
#13	Expanders: Apply equivalent subjects<br>Source Types: Academic Journals	72

eTable 2. The PICOTS Format: Study Inclusion/Exclusion Criteria

Study Elements	Criteria for Inclusion	Criteria for Exclusion
Populations	• Adult participants aged 18 years or older and treated with antithrombotic agents (anticoagulant or antiplatelet) for any indications which addressed at least one of the outcome of interest<br>• Other subgroups analysis will be included if studies providing data to calculate the effect estimates of the outcome of interest	• In vitro or animal studies<br>• Studies including less than 50 participants will be excluded owing to they lacked statistically significant power
Interventions	• SRI therapy for any indications	• NA
Comparators	• Non-SRI users	• Studies without control groups
Outcomes	• Primary outcomes<br>❖ Major bleeding<br>❖ Brain hemorrhage<br>❖ GI bleeding<br>❖ Any bleeding<br>• Secondary outcomes<br>❖ Blood transfusion<br>❖ Endoscopy-refractory bleeding<br>❖ Rebleeding<br>❖ Bleeding-related death	• Studies not providing data to calculate the effect estimates of the outcome of interest
Timing	• An extensive search strategy from the inception of bibliographic databases forward to assure all published literature was identified	• No limit timing of start date
Setting	• Both RCTs and observational nonrandomized trials (cohort studies and case-control studies)<br>• Grey literature and ongoing trial will be browsed<br>• Studies will not be limited language	• N-of-one, cross-sectional, case series/case reports, pharmacokinetic/pharmacodynamics study, and phase I or II study design<br>• Reports not involving primary data including, narrative review, systematic review, meta-analysis, news items, consensus statement, guidelines, and opinion/editorials

Abbreviations: GI, gastrointestinal; NA, not applicable; PICOTS, populations, interventions, comparators, outcomes, timing, setting; RCTs, randomized controlled trial; SRI, serotonin-reuptake inhibitors.

eTable 3. Measurement and Definition of Bleeding Events of Included Studies

First Author, Year	Outcomes Measurement	Clinical Endpoint Definition				
		**Major Bleeding**	**Brain Hemorrhage**	**Bleeding in Surgical Setting**	**GI Bleeding**	**Any Bleeding**
Kurdyak et al,[1] 2005	• Multiple-linked database ICD-9 codes	• NR	• NR	• NR	• Hospitalization with UGIB: ICD-9 codes<br>• Positive predictive value of 86% for UGIB	• NR
Kharofa et al,[2] 2007	• Medical record and discharge ICD-9 codes<br>• Prospective screening of neurosurgery admissions	• NR	• ED diagnoses of ICH and SAH, ICD-9 discharge<br>• Screening of admission in neurosurgery department	• NR	• NR	• NR
de Abajo et al,[3] 2008	• EHRs: THIN Database	• NR	• NR	• NR	• Medical diagnoses with read codes (conformation rate of 97% with general practitioner review)	• NR
Schalekamp et al,[4] 2008	• EHRs: PHARMO Record Linkage System	• Hospitalization with abnormal bleeding: GI tract, intracranial, uterus, urinary tract, joint, eye, nose, hemoptysis, bleeding complicating a procedure, hemoperitoneum, spontaneous ecchymosed (ICD-9-CM codes)	• Hospitalization with abnormal bleeding: intracranial (ICD-9-CM codes)	• NR	• Hospitalization with abnormal bleeding: GI tract (ICD-9-CM codes)	• NR

Abbreviations: ED, emergency department; EHRs, electronic health records; GI, gastrointestinal; ICD, International Classification of Diseases; ICH, intracerebral hemorrhage; NR, not reported; SAH, subarachnoid hemorrhage; THIN, The Health Improvement Network; UGIB, upper gastrointestinal tract bleeding.

eTable 3. Measurement and Definition of Bleeding Events of Included Studies (Continued)

First Author, Year	Outcomes Measurement	Clinical Endpoint Definition				
		Major Bleeding	Brain Hemorrhage	Bleeding in Surgical Setting	GI Bleeding	Any Bleeding
Dall et al,[5] 2009	• EHRs: from 3 different sources; the FPAS, OPED, and Danish CPR<br>• ICD-8, ICD-9, and ICD-10	• Bleeding event that required an intervention; included holding ≥1 dose of warfarin administering, blood transfusion, ED visit, or hospitalization	• NR	• NR	• Admission with peptic ulcer or gastritis as main diagnosis and have significant bleeding (either melena, subnormal Hb, or transfusions)<br>• Potential bleeding source in the stomach/duodenum (identified by endoscopy or surgery)	• NR
Wallerstedt et al,[6] 2009	• Medical record: local patient database	• Bleeding-related hospital admission (ICD-10 codes)	• NR	• NR	• NR	• NR
Cochran et al,[7] 2011	• Medical record (NS)	• Bleeding event that required an intervention during 6-month assessment period,<br>• Intervention including, holding ≥1 dose of warfarin, administering phytonadione, blood transfusion, ED visit, or hospitalization	• NR	• NR	• NR	• Any bleeding during 6-month assessment period

Abbreviations: CPR, Central Person Register; ED, emergency department; EHRs, electronic health record; FPAS, Funen County Patient Administrative System; GI, gastrointestinal; Hb, hemoglobin; ICD, International Classification of Diseases; NR, not reported; NS, not specified; OPED, Odense University Pharmacoepidemiological Database;

eTable 3. Measurement and Definition of Bleeding Events of Included Studies (Continued)

First Author, Year	Outcomes Measurement	Clinical Endpoint Definition				
		Major Bleeding	Brain Hemorrhage	Bleeding in Surgical Setting	GI Bleeding	Any Bleeding
Labos et al,[8] 2011	• EHRs: the provincial health services administrative databases	• Bleeding episode (gastrointestinal bleeding, hemorrhagic stroke or other bleeding) that either necessitated admission to hospital or occurred in in-hospital during follow-up<br>• Diagnostic codes (ICD-9/ICD-10)	• NR	• NR	• Diagnostic codes (ICD-9/ICD-10)	• NR
Schelleman et al,[9] 2011	• EHRs: existing data of CMS Database of California, Florida, New York, Ohio, and Pennsylvania	• NR	• NR	• NR	• Hospitalized with a principal or non-principle ICD-9 code for GI bleeding—a positive predictive value of 81%	• NR
Vitry et al,[10] 2011	• EHRs: Australian Department of Veterans' Affairs administrative claims database	• Bleeding-related hospital admission (ICD-10 of hemorrhage)	• NR	• NR	• NR	• NR

Abbreviations: CMS, Centers for Medicare and Medicaid Services; ED, emergency department; EHRs, electronic health record; GI, gastrointestinal; ICD, International Classification of Diseases; NR, not reported.

eTable 3. Measurement and Definition of Bleeding Events of Included Studies (Continued)

First Author, Year	Outcomes Measurement	Clinical Endpoint Definition				
		Major Bleeding	Brain Hemorrhage	Bleeding in Surgical Setting	GI Bleeding	Any Bleeding
Baillargeon et al,[11] 2012	• EHRs: Medicare beneficiaries, including Medicare enrollment files, MEDPAR files, Outpatient Standard Analytic Files, Medicare Carrier files, and Prescription Drug Event records	• Bleeding requiring hospitalization at any time in 2008 based on ICD-9-CM, in the primary diagnosis position, from MEDPAR files: GI, non-GI, intracranial, and general warfarin toxicity	• NR	• NR	• NR	• NR
Lin et al,[12] 2013	• EHRs: NHIRD—LHID2000, including enrollment files, claims data, catastrophic illness files, and registry for drug prescription	• NR	• NR	• NR	• Hospitalized with a nonvariceal UGIB and LGIB as the main diagnosis (ICD-9-CM)	• NR
Mosholder et al,[13] 2013	• EHRs: CMS nationwide data, including Medicare Parts A, B, and D beneficiaries	• Major bleeding using inpatient or ED ICD-9 codes for epistaxis, GI bleeding, hematuria, and intracranial bleeding	• ICD-9 codes	• NR	• ICD-codes	• NR
Seitz et al,[14] 2013	• Medical record with a primary diagnosis of hip fracture and underwent surgery (ICD-10 code S72)	• NR	• NR	• Receipt of any RBC transfusion during hospital admission for hip fracture surgery	• NR	• NR

Abbreviations: CMS, Centers for Medicare and Medicaid Services; ED, emergency department; EHRs, electronic health record; GI, gastrointestinal ICD, International Classification of Diseases; LGIB, lower gastrointestinal tract bleeding; LHID, Longitudinal Health Insurance Database; MEDPAR, Medicare Provider Analysis and Review; NHIRD, National Health Insurance Research Database; NR, not reported; RBC, red blood cell; UGIB, upper gastrointestinal tract bleeding.

eTable 3. Measurement and Definition of Bleeding Events of Included Studies (Continued)

First Author, Year	Outcomes Measurement	Clinical Endpoint Definition				
		Major Bleeding	Brain Hemorrhage	Bleeding in Surgical Setting	GI Bleeding	Any Bleeding
Giang et al,[15] 2014	• EHRs: VASDHS Database	• NR	• NR	• NR	• NR	• Using the TIMI bleeding criteria (major, minor, and minimal) and other minimal bleeding types (non-TIMI)
Nguyen et al,[16] 2014	• NR	• NR	• NR	• NR	• NR	• NS
Quinn et al,[17] 2014	• EHRs and medical chart review based on previously validated algorithms<br>• Hospitalizations for incident major hemorrhages	• Fatal, requiring ≥2 units of transfused blood, or hemorrhage into a critical anatomic site (e.g. intracranial, retroperitoneal, or intraocular)	• Primary and secondary diagnosed (ICD-9 code), including ICH or SAH	• NR	• NR	• NR
Rashid et al,[18] 2016	• NR	• Bleeding within 30 days of ACS according to BARC criteria 3-5	• NR	• NR	• NR	• Bleeding within 30 days of ACS according to BARC criteria 1-5
Lai et al,[19] 2017	• EHRs, Sutter Health—an integrated healthcare system in Northern California (includes 24 centers)	• NR	• NR	• NR	• Inpatient diagnosis: endoscopic examinations were extracted by chart review	• NR

Abbreviations: ACS, acute coronary syndrome; BARC, Bleeding Academic Research Consortium; EHRs, electronic health record; GI, gastrointestinal ICD, International Classification of Diseases; ICH, intracerebral hemorrhage; NR, not reported; NS, not specified; SAH, subarachnoid hemorrhage; TIMI, Thrombolysis in Myocardial Infarction; VASDHS, VA San Diego Healthcare System.

eTable 3. Measurement and Definition of Bleeding Events of Included Studies (Continued)

First Author, Year	Outcomes Measurement	Clinical Endpoint Definition				
		Major Bleeding	Brain Hemorrhage	Bleeding in Surgical Setting	GI Bleeding	Any Bleeding
Laursen et al,[20] 2017	• Danish Clinical Registry of Emergency Surgery	• NR	• NR	• Endoscopy-refractory bleeding: bleeding that could not be controlled by endoscopic therapy<br>• Rebleeding: further symptoms of PUB within 5 days of initial endoscopy; decline in Hb or confirmed by endoscopy, surgery or transcatheter arterial embolism	• NR	• NR
Renoux et al,[21] 2017	• EHRs: United Kingdom's CPRD	• NR	• Read code related intracranial hemorrhage, including ICH not otherwise specified, nontraumatic suburalor extradural or extradural hematoma, hemorrhagic stroke, ICH, or SAH	• NR	• NR	• NR
Samuel et al,[22] 2017	• EHRs: tertiary care referral country teaching hospital (University Medical Center, Lubbock, TX)	• Defined as the ISTH-SCC: fatal bleeding, and/or bleeding causing a fall in Hb level ≥2 g/dL, or leading to transfusion of ≥2 units of whole blood or red cells	• NR	• NR	• NR	• NR

Abbreviations: CPRD, Clinical Practice Research Datalink; EHRs, electronic health record; GI, gastrointestinal; Hb, hemoglobin; ICH, intracerebral hemorrhage; ISTH-SCC, International Society on thrombosis and Haemostasis/Scientific and Standardization Committee; NR, not reported; PUB, peptic ulcer bleeding; SAH, subarachnoid hemorrhage.

eTable 3. Measurement and Definition of Bleeding Events of Included Studies (Continued)

First Author, Year	Outcomes Measurement	Clinical Endpoint Definition				
		Major Bleeding	Brain Hemorrhage	Bleeding in Surgical Setting	GI Bleeding	Any Bleeding
Scheitz et al,[23] 2017	• Prospective registries data	• NR	• Occurrence of symptomatic ICH according to ECASS II criteria; any ICH associated with an increase in the NIHSS score of at least 4 points within 36 hours after thrombolysis	• NR	• NR	• NR
Quinn et al,[24] 2018	• Along with ROCKET AF trial followed up	• Clinically overt bleeding associated with any of the following: fatal outcome, involvement of a critical anatomic site (intracranial, spinal, ocular, pericardial, articular, retroperitoneal, or intramuscular with compartment syndrome), fall in Hb ≥2 g/dL, transfusion of ≥2 units of whole blood or packed RBC, or permanent disability	• NR	• NR	• NR	• Composite of major bleeding and non-major clinically relevant bleeding as following overt bleeding not meeting criteria for major bleeding but requiring medical intervention, unscheduled contact with a physician, temporary interruption of study drug, pain, or impairment of daily activities

Abbreviations: ECASS, European Cooperative Acute Stroke Study; GI, gastrointestinal; Hb, hemoglobin; ICH, intracerebral hemorrhage; NIHSS, National Institutes of Health Stroke Scale; NR, not reported; RBC, red blood cell.

eTable 3. Measurement and Definition of Bleeding Events of Included Studies (Continued)

First Author, Year	Outcomes Measurement	Clinical Endpoint Definition				
		Major Bleeding	Brain Hemorrhage	Bleeding in Surgical Setting	GI Bleeding	Any Bleeding
Iasella et al,[25] 2019	• Medical record review through EHRs	• Composite endpoint 1-year follow-up by modified TIMI and GUSTO criteria<br>• Decrease in Hb of ≥5 g/dL, transfusion of ≥4 units of packed red blood cells, or remission with the presence of a primary admission ICD-9-CM code for bleeding	• NR	• NR	• NR	• Composite of 1-year follow-up major bleeding and minor bleeding by modified TIMI and GUSTO criteria<br>• Mainor bleeding defined as a decrease in Hb of ≥2 g/dL but <5 g/dL, or the needs for ≥2 units but <4 unit of packed RBC
Luo et al,[26] 2019	• EHRs: NHIRD—LHID2000, including enrollment files, claims data, catastrophic illness files, and registry for drug prescription	• NR	• NR	• NR	• Hospitalized with a nonvariceal UGIB as the main diagnosis (ICD-9-CM codes)	• NR

Abbreviations: EHRs, electronic health record; GI, gastrointestinal; GUSTO, Global Use of Strategies to Open Occluded Coronary Arteries; Hb, hemoglobin; ICD, International Classification of Diseases; LHID, Longitudinal Health Insurance Databases; NHIRD, National Health Insurance Research Database; NR, not reported; RBC, red blood cell; TIMI, Thrombolysis in Myocardial Infarction.

eTable 3. Measurement and Definition of Bleeding Events of Included Studies (Continued)

First Author, Year	Outcomes Measurement	Clinical Endpoint Definition				
		Major Bleeding	Brain Hemorrhage	Bleeding in Surgical Setting	GI Bleeding	Any Bleeding
Gaist et al,[27] 2020	• EHRs: Danish National patient Registry, Danish National Prescription Registry, Danish Civil Registry System	• NR	• Defined as the ICD-10 code (S065): traumatic SDH in Denmark with a positive predictive value of 96%	• NR	• NR	• NR
Komen et al,[28] 2020	• EHRs: Stockholm healthcare database	• Severe bleed, using ICD-10 codes with a positive predictive value of 95.5%	• ICD-10 codes with a positive predictive value of 95.5%	• NR	• ICD-10 codes with a positive predictive value of 95.5%	• NR
Lee et al,[29] 2020	• EHRs: National Health Information Database (NHIS-2019-1-402), a single-payer organization in Korea	• Hospitalization or emergency department visit for major bleeding events using ICD-10 CM	• Defined as the ICD-10 code: intracranial bleeding	• NR	• Defined as the ICD-10 code: UGIB, LGIB	• NR
Marchena et al,[30] 2020	• Prospective registry data	• Overt and required blood transfusion of ≥2 units, retroperitoneal, spinal, or intracranial	• Intracranial bleeding	• NR	• NR	• NR

Abbreviations: EHRs, electronic health record; GI, gastrointestinal; ICD, International Classification of Diseases; LGIB, lower gastrointestinal tract bleeding; NHIS, National Health Insurance Service; NR, not reported; SDH, subdural hematoma; UGIB, upper gastrointestinal tract bleeding.

eTable 3. Measurement and Definition of Bleeding Events of Included Studies (Continued)

First Author, Year	Outcomes Measurement	Clinical Endpoint Definition				
		Major Bleeding	Brain Hemorrhage	Bleeding in Surgical Setting	GI Bleeding	Any Bleeding
Mawardi et al,[31] 2019	• Medical record review	• NR	• NR	• NR	• Medical diagnosis with GI bleeding: confirmed by either a positive stool hemoccult test or endoscopic study	• NR
Zhang et al,[32] 2018	• EHRs: United Kingdoms' CPRD linked to secondary care data from the HES	• ICD-10 codes with primary discharge diagnosis of major bleeding: composite of GI, intracranial and other symptomatic bleeding in a critical area or organ defined use an adapted version of the definition of ISTH	• NR	• NR	• Defined as the ICD-10 code	• NR

Abbreviations: CPRD, Clinical Practice Research Datalink; EHRs, electronic health record; GI, gastrointestinal; HES, Hospital Episode Statistics; ICD, International Classification of Diseases; ISTH, International Society on Thrombosis and Haemostasis; NR, not reported.

eTable 4. Methods of Included Studies in the Meta-Analysis

First Author, Year	Population/Case Patients	Selection of Control	SRIs Use Defined as	Analysis Method	Factors Controlled for in Analysis
Kurdyak et al,[1] 2005	• Elderly patients (>65 years) who received warfarin continuously for at least 1 year before hospitalization for UGIB	• Ten controls for each case, matching on age (within 30 days of birth date), sex, and continuous use of warfarin on the index date	• Exposure 90 days before UGIB hospitalization	• Multivariable conditional logistic regression models	• Matching variables<br>• Previous hospitalizations for UGIB, medication (NSAIDs, aspirin, glucocorticoids, PPI/H2RA), CYP2C9 inhibitors and inducers, diabetes, and medical comorbidity (drug use proxy)
Kharofa et al,[2] 2007	• Cases were all emergency visit and discharge ICD-9 codes with ICH and SAH	• Controls were matched by age, race, and gender to population-based controls by random digit dialing	• Exposure 2 weeks before index date	• Multivariable conditional logistic regression models	• Matching variables<br>• Alcohol use, heart disease, history of ischemic stroke, BMI, hypertension, untreated hypertension, statin, untreated hypercholesterolemia, smoking status, and education level
de Abajo et al,[3] 2008	• Adult patients aged 40-84 years who have been seen for at least 2 years by a general practitioner<br>• All patients who had a history of cancer, liver disease, coagulopathy, Mallory-Weiss syndrome, esophageal varices, or alcohol-related disorders were excluded	• Randomly selected from the sources population using a density-based sampling method<br>• Controls were matched by age (within 1 year), sex, and calendar year of the index year	• Current users: prescribed SRIs lasted until the index date or were discontinued within 30 days of the index date	• Multivariable unconditional logistic regression models	• Matching variables (age, sex, and calendar year), smoking status, alcohol intake, antecedents of GI disorder, and concomitant use of other medications associated with UGIB (NSAIDs, systemic corticosteroids)

Abbreviations: BMI, body mass index; CYP, cytochrome P450; GI, gastrointestinal; H2RAs, histamine H2-receptor antagonists; ICD, International Classification of Disease; ICH, intracerebral hemorrhage; NSAIDs, nonsteroidal anti-inflammatory drugs; PPIs, proton pump inhibitors; SAH, subarachnoid hemorrhage; SRIs, serotonin reuptake inhibitors; UGIB, upper gastrointestinal tract bleeding.

eTable 4. Methods of Included Studies in the Meta-Analysis (Continued)

First Author, Year	Population/Case Patients	Selection of Control	SRIs Use Defined as	Analysis Method	Factors Controlled for in Analysis
Schalekamp et al,[4] 2008	• Adult patients who received a first prescription of acenocoumarol or phenprocoumon<br>• Patients who had a history of hospital admission with major bleeding were excluded	• Up to 4 non-hospitalized control subjects were randomly selected from the cohort by risk-set sampling<br>• Controls were matched by sex, age (± 5 years), coumarin anticoagulant used (± 90 days from dispensing date), and geographical region	• Current users: use of SRIs extended with 10% ended on or beyond the index date	• Multivariable conditional logistic regression models	• Matching variables<br>• NSAIDs, antiplatelet agents, antibiotics, glucocorticoids, gastroprotective agents (PPIs, H2RAs, misoprostol), inhibitors and inducers of coumarin metabolism, and the comorbidities (diabetes, thyroid disorders, CHF, and cancer)
Dall et al,[5] 2009	• Adult patients who hospitalized with peptic ulcer/gastritis and have significant bleeding (either melena, subnormal Hb, or the need for transfusions)<br>• Patients with variceal bleeding were excluded	• Age and gender matched up to 10 controls were sampled by use of a risk set sampling technique	• Current users	• Multivariable conditional logistic regression models	• Matching variables<br>• Medication (PPIs, warfarin, clopidogrel, dipyridamole, steroids), alcohol abuse, cerebral ischemia, stroke, *Helicobacter* eradication, peptic ulcer, UGIB, and cirrhosis
Wallerstedt et al,[6] 2009	• Adult patients aged 55-80 years with warfarin due to AF at the Centre of Coagulation, Sahlgrenska University Hospital	• Cohort study: unexposed patients without SRIs treatment were randomly selected and matched for age and sex on a 1:1 basis	• Patients treated with SRIs at any time during study period<br>• Use of SRIs within 3 week of starting warfarin treatment were excluded	• Multivariable Cox regression models	• Age, sex, and medication (NSAIDs, aspirin, glucosamine)

Abbreviations: AF, atrial fibrillation; CHF, congestive heart failure; H2RAs, histamine H2-receptor antagonists; Hb, hemoglobin; NSAIDs, nonsteroidal anti-inflammatory drugs; PPIs, proton pump inhibitors; SRIs, serotonin reuptake inhibitors; UGIB, upper gastrointestinal tract bleeding.

eTable 4. Methods of Included Studies in the Meta-Analysis (Continued)

First Author, Year	Population/Case Patients	Selection of Control	SRIs Use Defined as	Analysis Method	Factors Controlled for in Analysis
Cochran et al,[7] 2011	• Adult outpatients who were treated with warfarin for ≥6 months and followed in the pharmacist-managed Antithrombosis Clinic at the University of Illinois Medical Center	• Cohort study: unexposed patients were matched by age and race	• Baseline: SSRIs users (NS)	• Multivariable logistic regression models	• Matching variables<br>• Sex, history of stroke, history of GI bleeding, hematocrit <30%, and serum creatinine >1.5 mg/dL
Labos et al,[8] 2011	• Adult patients aged ≥50 years who were discharged from hospital with a primary diagnosis of acute myocardial infarction (ICD-9 410.x or ICD-10 I21.x)<br>• Patients who had been admitted to hospital due to GI bleeding during the year before the discharge date, and those with any bleeding episode during the index admission were excluded	• Population-based cohort study: unexposed patients were selected from the same cohort	• SSRIs users (NS)	• Multivariable Cox regression models	• Age, sex, comorbidities (cancer, renal failure, CHF, anemia or other hematologic disease), angioplasty during index admission, anticoagulant use at discharge, corticosteroid use at discharge, antihyperglycemic use in prior year, antihypertensive use in prior year, PUD diagnosed in prior year, visit to gastroenterologist in prior year, other bleeding in prior year

Abbreviations: CHF, congestive heart failure; GI, gastrointestinal; ICD, International Classification of Disease; NS, not specified; PUD, peptic ulcer disease; SRIs, serotonin reuptake inhibitors; SSRIs, selective serotonin reuptake inhibitors.

eTable 4. Methods of Included Studies in the Meta-Analysis (Continued)

First Author, Year	Population/Case Patients	Selection of Control	SRIs Use Defined as	Analysis Method	Factors Controlled for in Analysis
Schelleman et al,[9] 2011	• Adults patients aged ≥18 years who exposed to warfarin (outpatient prescriptions only)<br>• Warfarin users who filled a prescription for an antidepressant of interest 90 days before or on the same days as their first observed warfarin prescription were excluded	• Fifty controls were selected at random for each case during eligible person-time exposed to warfarin<br>• Controls were matched by the index date and state, using incidence density sampling	• Exposed patients who received SRIs within 29 days prior or on the index date<br>• Patients who exposed to ≥2 different SRIs in the index date were excluded	• Multivariable conditional logistic regression models	• Matching variables<br>• Age, gender, race, no. of warfarin prescriptions filled on the index date, nursing home, use of dementia, liver disease, prior GI bleeding, renal disease, and medication (acetaminophen, levofloxacin, PPIs)
Vitry et al,[10] 2011	• Elderly veterans aged ≥65 years who had eligible for all health services subsidized by the Department of Veterans' Affairs<br>• New users of warfarin, defined as those not having had a prescription for warfarin dispensed in the previous 6 months	• Cohort study: non-SSRIs users	• Patients who prescribed with concurrent SSRIs at any time between Jul 2002 and Jun 2006<br>• SSRIs users was limited to a period of 28 days	• Multivariable Poisson regression models	• Age, sex, socioeconomic index, no. of comorbidities, no. of prescribers, no. of different pharmacies used, previous bleeding-related hospitalizations during the 1-yesr period before first warfarin prescription, no. of different medicines prescribed during the study period, residential status and if bleeding occurred in the first 2 weeks of warfarin initiation

Abbreviations: GI, gastrointestinal; PPIs, proton pump inhibitors; SRIs, serotonin reuptake inhibitors; SSRIs, selective serotonin reuptake inhibitors.

eTable 4. Methods of Included Studies in the Meta-Analysis (Continued)

First Author, Year	Population/Case Patients	Selection of Control	SRIs Use Defined as	Analysis Method	Factors Controlled for in Analysis
Baillargeon et al,[11] 2012	• Elderly patients aged ≥65 years who were continuous users of warfarin for ≥180 days in 2007; and have received at least one prescription for warfarin (for any duration) in 2008<br>• Hospitalized with bleeding event at any time in 2007 were excluded	• Three controls were selected for each case from the cohort of warfarin users<br>• Controls were matched by event month, indication for warfarin use, age, sex, race/ethnicity, and index of multiple indications for warfarin use	• Exposed patients who received SRIs at least 1 day in the 15 days before the event/index date	• Multivariable logistic regression models	• Matching variables<br>• Comorbidity index, having stayed in a nursing home in the 90 days before event/index date, medication (any antibiotic agent, corticosteroid, antiplatelet, CYP2C inhibitors
Lin et al,[12] 2013	• Adult patients who took clopidogrel with an average dose of >150 DDD per one-half year, those who took ticlopidine during study period were excluded	• Cohort study: non-SSRIs users	• Defined as a prescription of SSRIs for >4 weeks within 8 weeks before the index date	• Multivariable Cox regression models	• Age, sex, CAD, hypertension, diabetes, COPD, chronic renal disease, cirrhosis, ischemic stroke, uncomplicated PUD, PUB, dyslipidemia, and medication (NSAIDs, COX-II inhibitor, aspirin, steroid, warfarin, alendronate, H2RAs)
Mosholder et al,[13] 2013	• Adult patients treated warfarin for ≥1 month prior to a prescription for a concomitant SSRIs<br>• Bleeding within 30 days preceding the prescription SSRIs to warfarin were excluded	• Cohort study: non-SSRIs users	• Defined as prescription of SSRIs over a 30-day look back period	• Multivariable logistic regression models	• Age, sex, geographical region, income subsidy, recent hospitalization or ED visit, liver disease, cystitis, ulcer, diverticulosis, other chronic medical conditions, warfarin duration, and medication (NSAIDs, PPIs, platelet inhibitors, known drugs that interact with warfarin)

Abbreviations: CAD, coronary artery disease; COPD, chronic obstructive pulmonary disease; COX-II, cyclooxygenase-II; CYP, cytochrome P450; DDD, defined daily dose; ED, emergency department; H2RAs, histamine H2-receptor antagonists; NSAIDs, nonsteroidal anti-inflammatory drugs; PPIs, proton pump inhibitors; PUB, peptic ulcer bleeding; PUD, peptic ulcer disease; SRIs, serotonin reuptake inhibitors; SSRIs, selective serotonin reuptake inhibitors.

eTable 4. Methods of Included Studies in the Meta-Analysis (Continued)

First Author, Year	Population/Case Patients	Selection of Control	SRIs Use Defined as	Analysis Method	Factors Controlled for in Analysis
Seitz et al,[14] 2013	• Elderly patients aged ≥66 years who underwent hip fracture surgery<br>• Individuals who had received palliative care or had a hip fracture associated with major trauma were excluded	• Cohort study: control group was recent former users of high-affinity SRIs—had at least1 antidepressant in the 3 years preceding hip fracture with no prescription within 90 days of hip fracture	• Defined as current users of high-affinity of SRIs—who had a minimum of 2 antidepressant prescriptions in the time period preceding hip fracture with the date of last antidepressant prescription overlapping the date of surgery<br>• Current users needs to have a minimum of 60 days of antidepressant therapy	• Multivariable logistic regression models	• Age, sex, Charlson comorbidity score, total number of unique medications in the year preceding index, place of residence
Giang et al,[15] 2014	• Adult veterans aged ≥18years and receiving DAPT following coronary stent placement<br>• The study observation period started from the first dose of ADP receptor antagonists and continued for 12 months	• Cohort study: non-SSRIs users	• Defined as prescription of SSRIs during the observation period	• NS	• None
Nguyen et al,[16] 2014	• Adult veterans who taking warfarin between 2009 and 2011	• Cohort study: non-SSRI users (NS)	• SSRIs users(NS)	• NS	• None
Quinn et al,[17] 2014	• Adult patients enrolled in the ATRIA study with diagnosed AF from Jul, 1996, to Dec 1997 and followed up for a median of 6 years	• Cohort study: non-SRIs users	• Exposure to SRIs defined as receipt ≥ prescriptions found in pharmacy database dispensing data	• Multivariable Poisson regression models	• ATRIA bleeding risk score and time in INR range ≥3.0

Abbreviations: ADP, adenosine diphosphate; AF, atrial fibrillation; ATRIA, AnTicoagulation and Risk factors In Atrial fibrillation; DAPT, dual antiplatelet therapy; INR, international normalized ratio; NS, not specified; SRIs, serotonin reuptake inhibitors; SSRIs, selective serotonin reuptake inhibitors.

eTable 4. Methods of Included Studies in the Meta-Analysis (Continued)

First Author, Year	Population/Case Patients	Selection of Control	SRIs Use Defined as	Analysis Method	Factors Controlled for in Analysis
Rashid et al,[18] 2016	• Adult patients with acute coronary syndrome who received DAPT following angioplasty<br>• Death within 24 hours of admission was excluded	• Cohort study: non-SSRIs users (NS)	• SSRIs users (NS)	• NS	• None
Lai et al,[19] 2017	• Adult patients aged ≥18 years who received DOACs (rivaroxaban, dabigatran, or apixaban) for ≥3 month	• Cohort study: non-SSRIs users (NS)	• SSRIs users (NS)	• Univariate analysis (NS)	• None
Laursen et al,[20] 2017	• Adult patients who admitted to hospital with PUB<br>• Use of low-dose aspirin ≤150 mg/d	• Cohort study: non-SSRIs	• SSRIs users: any SSRI-agent, irrespective of dosage, at time of hospitalization	• Multivariable logistic regression models	• Age, ASA-score, high alcohol consumption, daily smoking status, Hb, duodenal ulcer location, year of admission to hospital, and medication (anticoagulants/ADP receptor inhibitors, NSAIDs, steroids)
Renoux et al,[21] 2017	• Adult patients aged ≥18 years who prescribed antithrombotic agents within 1 month before index date	• Randomly selected as many as 30 among the cohort members after matching for sex, age, calendar year of cohort entry, and duration of follow-up<br>• Reference was current (within 30 days before index date) use of TCAs	• New current users of SSRIs—the duration of their last prescription included the index date or ended within 30 days before the index date	• Multivariable conditional logistic regression models	• Matching variables<br>• Age, sex, BMI, smoking status, alcohol abuse, hyperlipidemia, hypertension, diabetes, AF, TIA, CAD, CHF, PVD, COPD, renal failure, depression, cancer, liver disease, disorders of hemostasis, brain vascular disease, history of bleeding, no. of physician visits, NSAIDs, antipsychotic, pain

Abbreviations: ADP, adenosine diphosphate; AF, atrial fibrillation; ASA, American Society of Anesthesiologists; BMI, body mass index; CAD, coronary artery disease; CHF, congestive heart failure; COPD, chronic obstructive pulmonary disease; DAPT, dual antiplatelet therapy; DOACs, direct oral anticoagulants; Hb, hemoglobin; NS, not specified; NSAIDs, nonsteroidal anti-inflammatory drugs; PUB, peptic ulcer bleeding; PVD, peripheral vascular disease; SRIs, serotonin reuptake inhibitors; SSRIs, selective serotonin reuptake inhibitors; TIA, transient ischemic attack.

eTable 4. Methods of Included Studies in the Meta-Analysis (Continued)

First Author, Year	Population/Case Patients	Selection of Control	SRIs Use Defined as	Analysis Method	Factors Controlled for in Analysis
Samuel et al,[22] 2017	• Adult patients aged >18 and ≤89 years, with a primary or secondary diagnosis of an acute pulmonary embolism or deep-vein thrombosis, and treated with full dose of enoxaparin	• Cohort study: non-SSRIs users (NS)	• SSRIs users (NS)	• Multivariable logistic regression models	• Alcohol use, acid suppressive therapy
Scheitz et al,[23] 2017	• Patients with ischemic stroke aged ≥18 treated with intravenous thrombolysis—alteplase within 4.5-hour time window<br>• Preadmission use of oral anticoagulants (INR <1.7)	• Cohort study: no preadmission use of SSRIs	• Preadmission use of SSRIs (NS)	• Multivariable logistic regression models	• Age, independent status before stroke, NIHSS score, additional endovascular therapy, systolic blood pressure, glucose, diabetes, and use of antiplatelet
Quinn et al,[24] 2018	• Adult patients undergoing the ROCKET AF trial treated with rivaroxaban or warfarin for the prevention of stroke or systemic embolism	• Cohort study: non-SSRIs users	• SSRIs users: medication record listed at any time throughout the ROCKET AF trial	• Propensity score matching 1:1 ratio for SSRIs users and non-users<br>• Multivariable Cox regression models	• Age, sex, geographical region, prior stroke/TIA, prior GI bleeding, COPD, diastolic blood pressure, creatinine clearance, platelets, albumin, medication (aspirin, vitamin K antagonist/thienopyridine, NSAIDs, randomized anticoagulant treatment)

Abbreviations: COPD, chronic obstructive pulmonary disease; DOACs, direct oral anticoagulants; GI, gastrointestinal; INR, international normalized ratio; NIHSS, National Institutes of Health Stroke Scale; NR, not reported; NS, not specified; NSAIDs, nonsteroidal anti-inflammatory drugs; ROCKET AF, Rivaroxaban once daily Oral direct factor xa inhibition Compared with vitamin K antagonism for prevention of Embolism and stroke Trial in Atrial Fibrillation; SRIs, serotonin reuptake inhibitors; SSRIs, selective serotonin reuptake inhibitors; TIA, transient ischemic attack.

eTable 4. Methods of Included Studies in the Meta-Analysis (Continued)

First Author, Year	Population/Case Patients	Selection of Control	SRIs Use Defined as	Analysis Method	Factors Controlled for in Analysis
Iasella et al,[25] 2019	• Adult patients treated with clopidogrel-based DAPT following percutaneous coronary intervention procedures	• Cohort study: non-SSRIs users	• SSRIs users: use of any duration	• Multivariable Cox regression models	• Age, race, sex, history of valvular disease, CHF, chronic kidney disease, dyslipidemia, COPD, coronary artery bypass graft, hypertension
Luo et al,[26] 2019	• Adult patients aged >20 years who took aspirin with an average dose of >14 DDD per month	• Cohort study: non-SSRIs users	• SSRIs users: prescription for ≥14 days within 1 months before the index date or censoring	• Multivariable Cox regression models	• Age, sex, CAD, hypertension, diabetes, COPD, chronic renal disease, cirrhosis, ischemic stroke, dyslipidemia, uncomplicated PUD, and medication (NSAIDs, COX-II inhibitor, steroids, warfarin, dipyridamole, thienopyridines, medication of *H. pylori* eradication)
Gaist et al,[27] 2020	• Adult patients aged 20-89 with current use (supply with grace period extended [60 days] up to cover index date) of antithrombotic agents (low-dose aspirin, clopidogrel, warfarin, or DOACs)	• Using risk sampling method, up to 40 controls were matched to birth year, and sex	• Current use of SSRIs: prescriptions dispended supply with grace period extended (60 days) up to cover index date	• Multivariable conditional logistic regression models	• Matching variables<br>• Age, sex, calendar period, socioeconomic status (education level and income), hypertension, stroke, epilepsy, dementia, COPD, high alcohol consumption, chronic hepatic disease, chronic renal insufficiency, diabetes, myocardial infarction, angina, unstable angina, PVD, and medication (low-dose aspirin, clopidogrel, DOACs, vitamin K antagonist, NSAIDs, hormone replacement therapy, steroids)

Abbreviations: CHF, congestive heart failure; CHF, congestive heart failure; COPD, chronic obstructive pulmonary disease; COX-II, cyclooxygenase-II; DAPT, dual antiplatelet therapy; DDD, defined daily dose; DOACs, direct oral anticoagulants; NSAIDs, nonsteroidal anti-inflammatory drugs; PVD, peripheral vascular disease; SRIs, serotonin reuptake inhibitors; SSRIs, selective serotonin reuptake inhibitors.

eTable 4. Methods of Included Studies in the Meta-Analysis (Continued)

First Author, Year	Population/Case Patients	Selection of Control	SRIs Use Defined as	Analysis Method	Factors Controlled for in Analysis
Komen et al,[28] 2020	• Adult AF patients with a new prescription for either DOACs or warfarin<br>• Patients who had a prescription for any oral anticoagulants during the year prior to inclusion were excluded	• Cohort study: non-antidepressant users	• Current antidepressant use: treatment episode from the claim of an antidepressant prescription until the calculated end of the treatment period	• Multivariable Cox regression models	• Age, sex, oral anticoagulants class, year of inclusion, years since AF diagnosis, anemia, prior major bleeding, stroke/TIA/embolism, alcoholism, hypertension, renal disease, CHF, diabetes, vascular disease, cancer, COPD, history of ≥2 falls, dementia/delirium/other mental disorder, schizophrenia, mood disorder, anxiety, behavioral syndromes, disorder in personality and behavior, unspecified mental disorder, and medication (aspirin, NSAIDs, clopidogrel, other antiplatelet, steroids, diuretics, beta-blocker, calcium channel blocker, RAAS inhibitor, lipid lowering agent, antidiabetic drug, gastro protective agent)
Lee et al,[29] 2020	• Adult AF patients (ICD-10, I48) with a new prescription for DOACs (apixaban, rivaroxaban, edoxaban, dabigatran)	• Up to 20 controls were randomly selected by age (± 5 years), sex, episode status, and duration from diagnosis of AF to the prescription of DOACs (± 1 year)	• SSRIs users overlapped during a continuous DOACs treatment episode for at least 3 days	• Multivariable conditional logistic regression models	• Matching variables<br>• Age, sex, myocardial infarction, dementia, liver/renal disease), and medications (beta-blocker, calcium channel blocker, angiotensin II receptor blockers, statins, diuretics)

Abbreviations: AF, atrial fibrillation; CHF, congestive heart failure; COPD, chronic obstructive pulmonary disease; DOACs, direct oral anticoagulants; ICD, International Classification of Diseases; NSAIDs, nonsteroidal anti-inflammatory drugs; RAAS, renin-angiotensin-aldosterone system; SRIs, serotonin reuptake inhibitors; SSRIs, selective serotonin reuptake inhibitors; TIA, transient ischemic attack.

eTable 4. Methods of Included Studies in the Meta-Analysis (Continued)

First Author, Year	Population/Case Patients	Selection of Control	SRIs Use Defined as	Analysis Method	Factors Controlled for in Analysis
Marchena et al,[30] 2020	• Registro Infromatizado Enfermedad TromboEmbolica (RIETE) registry cohort: adult patients diagnosed with venous thromboembolism (deep vein thrombosis or pulmonary embolism)	• Cohort study: non psychotropic users	• Baseline: SSRI/SNRI users	• Multivariable Cox regression models	• For major bleeding: age, sex, body weight, CHF, creatinine clearance levels, recent major bleeding, anemia, cancer, transient risk factors for venous thromboembolism (recent immobility, recent surgery, estrogen use, pregnancy/puerperium), initial venous thromboembolism presentation (deep vein thrombosis vs. pulmonary embolism), and medication (antiplatelet drugs, corticosteroids)<br>• For intracranial bleeding: age, body weight, CHF, creatinine clearance levels, recent major bleeding, anemia, cancer, transient risk factors for venous thromboembolism (recent immobility, recent surgery, estrogen use, pregnancy/puerperium), and medication (antiplatelet drugs, corticosteroids)
Mawardi et al,[31] 2019	• Adult patients undergoing left ventricular assist device treated with warfarin and aspirin (81 or 325 mg) per institutional protocols	• Cohort study: non-SSRIs/SNRIs users	• SSRI/SNRI users: prescription for ≥ 30 contiguous days post-left ventricular assist device	• Multivariable logistic regression models	• Age, ischemic cardiomyopathy, and platelet count

Abbreviations: AF, atrial fibrillation; CAD, coronary artery disease; CHF, congestive heart failure; COPD, chronic obstructive pulmonary disease; DOACs, direct oral anticoagulants; SRIs, serotonin reuptake inhibitors; SSRIs, selective serotonin reuptake inhibitors.

eTable 4. Methods of Included Studies in the Meta-Analysis (Continued)

First Author, Year	Population/Case Patients	Selection of Control	SRIs Use Defined as	Analysis Method	Factors Controlled for in Analysis
Zhang et al,[32] 2020	• Adult patients with a new prescription for DOACs (apixaban, dabigatran, rivaroxaban)	• Up to 4 controls were matched to each case based on age (± 1 year), sex, region, and index date	• SSRIs users: within 30 days window prior to the index date	• Multivariable conditional logistic regression models	• Matching variables<br>• Smoking, history of major bleeding, stroke/TIA, diabetes, hypertension, myocardial infarction, CHF, chronic renal disease, hepatic impairment, peripheral vascular disease, COPD, peptic ulcer disease, cancer, and medication (beta-blocker, angiotensin-converting enzyme inhibitors, non-P-glycoprotein inhibitor statins, proton pump inhibitors, cytochrome P450 enzyme inducers)

Abbreviations: CHF, congestive heart failure; COPD, chronic obstructive pulmonary disease; DOACs, direct oral anticoagulants; SRIs, serotonin reuptake inhibitors; SSRIs, selective serotonin reuptake inhibitors; TIA, transient ischemic attack.

eTable 5. Characteristics of Study Participants Included in the Meta-Analysis

First Author, Year	Heavy Alcohol Use, n (%)	Current smoker, n (%)	Diabetes, n (%)	AF, n (%)	CHF, n (%)	CAD, n (%)	Renal Failure, n (%)	Cancer, n (%)	*H. pylori*, n (%)	Prior bleeding, n (%)	SRIs, n (%)	NSAIDs, n (%)	Steroid, n (%)	PPIs/ H2RAs, n (%)
Kurdyak et al,[1] 2005	NR	NR	2,627 (15.7%)	NR	NA	NR	NR	NR	NR	195 (1.2%)[a]	1,036 (6.2%)	1,549 (9.2%)	855 (5.1%)	3,430 (20.5%)
Kharofa et al,[2] 2007[b]	176 (6.5%)	795 (29.5%)	NR	NR	NA	478 (17.8%)	NR	NR	NR	NR	229 (8.5%)	NR	NR	NR
de Abajo et al,[3] 2008	214 (1.9%)	1,800 (15.9%)	NR	NR	NA	NR	NR	0 (0.0%)	NR	NR	423 (3.7%)	815 (7.2%)	NR	1,372 (12.1%)
Schalekamp et al,[4] 2008	NR	NR	1,203 (15.7%)	NR	2,088 (27.2%)	NR	NR	129 (1.7%)	NR	0 (0.0%)	174 (2.3%)	521 (6.8%)	289 (3.8%)	1,016 (13.2%)[c]
Dall et al,[5] 2009[b]	688 (1.7%)[d]	NR	1,565 (3.9%)	NR	2,269 (5.6%)	4,029 (10.0%)	237 (0.6%)	NR	699 (1.7%)	305 (0.8%)[a]	5,488 (13.7%)	5,100 (12.7%)	1,799 (4.5%)	3,601 (9.0%)
Wallerstedt et al,[6] 2009	NR	NR	NR	234 (100.0%)	NR	NR	NR	NR	NR	NR	117 (50.0%)	63 (26.9%)	21 (9.0%)	NR
Cochran et al,[7] 2011	NR	NR	26 (26.0%)	22 (22.0%)	NR	1 (1.0%)	NR	10 (10.0%)	NR	13 (13.0%)[a]	25 (25.0%)	NR	NR	18 (18.0%)
Labos et al,[8] 2011	NR	NR	NR	NR	7,088 (26.2%)	27,058 (100.0%)	1,512 (5.6%)	1,207 (4.5%)	62 (0.2%)[e]	105 (0.4%)[f]	690 (2.6%)	176 (0.6%)	527 (1.9%)	5,352 (19.8%)
Schelleman et al,[9] 2011	NR	NR	NR	NR	NR	NR	98,498 (14.8%)	NR	NR	114,799 (17.2%)[a]	50,901 (7.6%)	22,822 (3.4%)	NR	126,480 (19.0%)[g]
Vitry et al,[10] 2011	NR	NR	NR	NR	NR	NR	NR	NR	NR	NR	2,767 (14.0%)[h]	NR	NR	NR
Baillargeon et al,[11] 2012	NR	NR	NR	1,736 (54.4%)	NR	NR	NR	NR	NR	NR	587 (18.4%)	NR	156 (4.9%)	NR

[a]History of gastrointestinal bleeding.
[b]Baseline characteristics were based on the entire study population.
[c]To Includes PPIs, H2RAs, and misoprostol.
[d]Based on alcohol related diagnosis: ICD-8, 295-300; ICD-10, F20, 30-33, KK70.0-K70.9, and F10.
[e]Based on use of Hp-PAC *Helicobacter pylori* infection.
[f]History of hemorrhagic stroke or non-gastrointestinal bleeding.
[g]To Include only PPIs.
[h]Number of episodes (incidence rate).
[i]To Includes only H2RAs.
[j]Baseline characteristics were based on the current and former serotonergic users.
Abbreviations: AF, atrial fibrillation; CAD, coronary artery disease; CHF, chronic heart failure; H2RAs, histamine H2-receptor antagonists; NSAIDs, non-steroidal anti-inflammatory drugs; PPIs, proton pump inhibitors; SRIs, serotonin reuptake inhibitors.

eTable 5. Characteristics of Study Participants Included in the Meta-Analysis (Continued)

First Author, Year	Heavy Alcohol Use, n (%)	Current smoker, n (%)	Diabetes, n (%)	AF, n (%)	CHF, n (%)	CAD, n (%)	Renal Failure, n (%)	Cancer, n (%)	*H. pylori*, n (%)	Prior bleeding, n (%)	SRIs, n (%)	NSAIDs, n (%)	Steroid, n (%)	PPIs/ H2RAs, n (%)
Lin et al,[12] 2013	NR	NR	1,447 (44.7%)	NR	NR	2,145 (66.2%)	611 (18.9%)	NR	NR	451 (13.9%)	52 (1.6%)	647 (20.0%)	189 (5.8%)	277 (8.6%)[i]
Mosholder et al,[13] 2013	NR	NR	NR	NR	NR	NR	NR	NR	NR	NR	NR	NR	NR	NR
Seitz et al,[14] 2013[j]	NR	NR	NR	NR	NR	3132 (36.5%)	1,059 (12.4%)	602 (7.0%)	NR	427 (5.0)[a]	NA	1,701 (19.8%)	550 (6.4%)	3,664 (42.8%)
Giang et al,[15] 2014	NR	NR	NR	NR	NR	162 (100.0%)	NR	NR	NR	NR	32 (19.8%)	NR	NR	NR
Nguyen et al,[16] 2014	NR	NR	NR	NR	NR	NR	NR	NR	NR	NR	527 (16.7%)	NR	NR	NR
Quinn et al,[17] 2014	NR	NR	1,902 (20.7%)	9,186 (100.0%)	3,426 (37.3%)	3,031 (33.0%)	NR	NR	NR	1,029 (11.2%)	542 (5.9%)	NR	NR	NR
Rashid et al,[18] 2016	NR	368 (43.9%)	215 (25.6%)	NR	NR	839 (100.0%)	NR	NR	NR	NR	NR	NR	NR	NR
Lai et al,[19] 2017	NR	NR	5,051 (23.5%)	15,234 (70.8%)	4,976 (23.1%)	5,176 (24.1%)	4,110 (19.1%)	NR	NR	NR	4,318 (20.1%)	NR	7,984 (37.1%)	9,674 (45.0%)
Laursen et al,[20] 2017[b]	2,222 (15.5%)	4,403 (30.7%)	NR	NR	NR	NR	NR	NR	NR	NR	NR	3,323 (23.2%)	1,105 (7.7%)	NR
Renoux et al,[21] 2017	758 (0.8%)	NR	10,020 (10.8%)	1,383 (1.5%)	1,062 (1.1%)	4,876 (5.2%)	3,526 (3.8%)	2,894 (3.1%)	NR	2,836 (3.1%)	14,929 (16.1%)[k]	21,605 (23.3%)	NR	NR
Samuel et al,[22] 2017	NR	NR	NR	NR	NR	NR	NR	NR	NR	0 (0.0%)	92 (16.0%)	16 (2.8%)	NR	NR

[a]History of gastrointestinal bleeding.
[b]Baseline characteristics were based on the entire study population.
[c]To Includes PPIs, H2RAs, and misoprostol.
[d]Based on alcohol related diagnosis: ICD-8, 295-300; ICD-10, F20, 30-33, KK70.0-K70.9, and F10.
[e]Based on use of Hp-PAC *Helicobacter pylori* infection.
[f]History of hemorrhagic stroke or non-gastrointestinal bleeding.
[g]To Include only PPIs.
[h]Number of episodes (incidence rate).
[i]To Includes only H2RAs.
[j]Baseline characteristics were based on the current and former serotonergic users.
Abbreviations: AF, atrial fibrillation; CAD, coronary artery disease; CHF, chronic heart failure; H2RAs, histamine H2-receptor antagonists; NSAIDs, non-steroidal anti-inflammatory drugs; PPIs, proton pump inhibitors; SRIs, serotonin reuptake inhibitors.

eTable 5. Characteristics of Study Participants Included in the Meta-Analysis (Continued)

First Author, Year	Heavy Alcohol Use, n (%)	Current smoker, n (%)	Diabetes, n (%)	AF, n (%)	CHF, n (%)	CAD, n (%)	Renal Failure, n (%)	Cancer, n (%)	*H. pylori*, n (%)	Prior bleeding, n (%)	SRIs, n (%)	NSAIDs, n (%)	Steroid, n (%)	PPIs/ H2RAs, n (%)
Scheitz et al,[23] 2017[b]	NR	NR	1,110 (17.8%)	1,668 (26.7%)	NR	NR	NR	NR	NR	NR	266 (4.3%)	NR	NR	NR
Quinn et al,[24] 2018	NR	NR	657 (44.6%)	1,474 (100.0%)	806 (54.7%)	273 (18.5%)	NR	NR	NR	NR	737 (50.0%)[l]	129 (8.8%)	NR	NR
Iasella et al,[25] 2019	NR	NR	2,449 (35.9%)	NR	1,300 (19.1%)	6,819 (100.0%)	863 (12.6%)	NR	NR	NR	820 (12.0%)	NR	NR	NR
Luo et al,[26] 2019	NR	NR	2,623 (23.6%)	NR	NR	1,227 (11.0%)	448 (4.0%)	NR	NR	NR	102 (0.9%)	1,967 (17.7%)	567 (5.1%)	NR
Gaist et al,[27] 2020[b]	22,557 (5.1%)	NR	43,321 (9.7%)	38,870 (8.7%)	NR	31,580 (7.1%)	7,369 (1.6%)	NR	NR	NR	30,991 (6.9%)	38,437 (8.6%)	NR	NR
Komen et al,[28] 2020[b]	1,449 (4.7%)	NR	5,741 (18.8%)	30,595 (100.0%)	7,276 (23.8%)	NR	2,539 (8.3%)	6,700 (21.9%)	NR	2,296 (7.5%)	2,625 (8.6%)[m]	3,797 (12.4%)	2,454 (8.0%)	6,163 (20.1%)
Lee et al,[29] 2020	NR	NR	10,713 (41.4%)	25,893 (100.0%)	11,065 (42.7%)	1,207 (4.7%)	1,024 (4.0%)	0 (0.0%)	NR	0 (0.0%)	1,130 (4.4%)	10,841 (41.9%)	NR	9,137 (35.3%)[g]
Marchena et al,[30] 2020	NR	NR	NR	NR	2,554 (5.4%)	NR	13,578 (28.8%)	9,786 (20.8%)	NR	962 (2.0%)	3,273 (7.0%)	2,463 (5.2%)	3,446 (7.3%)	NR
Mawardi et al,[31] 2019	NR	NR	NR	NR	NR	NR	NR	NR	NR	NR	105 (42.3%)	NR	NR	NR
Zhang et al,[32] 2020	NR	159 (8.4%)	363 (19.2%)	1,502 (79.6%)	323 (17.1%)	445 (23.6%)	163 (8.6%)	NR	NR	844 (44.7%)	136 (7.2%)	26 (1.4%)	NR	785 (41.6%)[g]

[a]History of gastrointestinal bleeding.
[b]Baseline characteristics were based on the entire study population.
[c]To Includes PPIs, H2RAs, and misoprostol.
[d]Based on alcohol related diagnosis: ICD-8, 295-300; ICD-10, F20, 30-33, KK70.0-K70.9, and F10.
[e]Based on use of Hp-PAC *Helicobacter pylori* infection.
[f]History of hemorrhagic stroke or non-gastrointestinal bleeding.
[g]To Include only PPIs.
[h]Number of episodes (incidence rate).
[i]To Includes only H2RAs.
[j]Baseline characteristics were based on the current and former serotonergic users.
Abbreviations: AF, atrial fibrillation; CAD, coronary artery disease; CHF, chronic heart failure; H2RAs, histamine H2-receptor antagonists; NSAIDs, non-steroidal anti-inflammatory drugs; PPIs, proton pump inhibitors; SRIs, serotonin reuptake inhibitors.

eTable 6. Risk of Bias Assessment of Included Studies: Using NOS—Cohort Studies[33]

First Author, Year	Selection				Comparability		Outcomes			Total NOS
	Representa-tiveness	Non-Exposed: Selection	Exposure: Ascertainment	Outcomes Not Present at Entry	Controls for: Age, sex, and history of bleeding	Control for: additional Factors[a]	Assessment	Follow-up Long Enough	Adequacy of follow-up	
Wallerstedt et al,[6] 2009	*	*	*	*	…….	…….	*	*	*	7
Cochran et al,[7] 2011	*	*	…….	*	*	*	*	*	*	8
Labos et al,[8] 2011	*	*	…….	*	*	*	*	*	*	8
Vitry et al,[10] 2011	*	*	*	*	*	…….	*	*	*	8
Lin et al,[12] 2013	*	*	*	*	…….	*	*	*	*	8
Mosholder et al,[13] 2013	*	*	*	*	…….	*	*	*	*	8
Seitz et al,[14] 2013	*	*	*	*	…….	…….	*	…….	*	6
Giang et al,[15] 2014	*	*	*	*	…….	…….	*	*	…….	6
Nguyen et al,[16] 2014	*	*	…….	*	…….	…….	…….	*	…….	4
Quinn et al,[17] 2014	*	*	*	*	…….	…….	*	*	*	7
Rashid et al,[18] 2016	*	*	…….	*	…….	…….	…….	…….	…….	3

[a]Study control for 3 of additional factors: race, alcohol use, diabetes, chronic hepatic disease, stages of kidney disease, CHF, COPD, stroke/TIA, hypertension, anemia, INR levels, NSAIDs, steroids, or PPIs/H2RAs/gastro protective agents.

Abbreviations: CHF, chronic heart failure; COPD, chronic obstructive pulmonary disease; H2RAs, histamine H2-receptor antagonists; INR, international normalized ratio; NSAIDs, nonsteroidal anti-inflammatory drugs; NOS, Newcastle-Ottawa Scale; PPIs, proton pump inhibitors; TIA, transient ischemic attack.

eTable 6. Risk of Bias Assessment of Included Studies: Using NOS—Cohort Studies[33] (Continued)

First Author, Year	Selection				Comparability		Outcomes			Total NOS
	Representa-tiveness	Non-Exposed: Selection	Exposure: Ascertainment	Outcomes Not Present at Entry	Controls for: Age, sex, and history of bleeding	Control for: additional Factors[a]	Assessment	Follow-up Long Enough	Adequacy of follow-up	
Lai et al,[19] 2017	*	*	…….	*	…….	…….	*	*	…….	5
Laursen et al,[20] 2017	*	*	*	*	…….	*	*	*	*	8
Samuel et al,[22] 2017	*	*	…….	*	…….	…….	*	*	*	6
Scheitz et al,[23] 2017	*	*	…….	*	…….	…….	*	*	*	6
Quinn et al,[24] 2018	*	*	*	*	*	*	*	*	*	9
Iasella et al,[25] 2019	*	*	…….	*	…….	*	*	*	*	7
Luo et al,[26] 2019	*	*	*	*	…….	*	*	*	*	8
Komen et al,[28] 2019	*	*	*	*	*	*	*	*	*	9
Machena et al,[30] 2020	*	*	…….	*	*	…….	*	*	*	7
Mawardi et al,[31] 2019	*	*	*	*	…….	…….	*	*	*	7

[a]Study control for 3 of additional factors: race, alcohol use, diabetes, chronic hepatic disease, stages of kidney disease, CHF, COPD, stroke/TIA, hypertension, anemia, INR levels, NSAIDs, steroids, or PPIs/H2RAs/gastro protective agents.

Abbreviations: CHF, chronic heart failure; COPD, chronic obstructive pulmonary disease; F/U, follow-up; H2RAs, histamine H2-receptor antagonists; INR, international normalized ratio; NSAIDs, nonsteroidal anti-inflammatory drugs; NOS, Newcastle-Ottawa Scale; PPIs, proton pump inhibitors; TIA, transient ischemic attack.

eTable 6. Risk of Bias Assessment of Included Studies: Using NOS—Case-Control Studies[33] (Continued)

First Author, Year	Selection				Comparability		Exposure			Total NOS
	Cases: Definition	Cases: Representa-tiveness	Controls: Selection	Controls: Definitions	Controls for: Age, sex, and history of bleeding	Control for: additional Factors[a]	Ascertainment	Same Method	Non-Response Rate	
Kurdyak et al,[1] 2005	*	*	*	*	*	*	*	*	*	9
Kharofa et al,[2] 2007	*	*	*	*	…….	*	*	*	*	8
de Abajo et al,[3] 2008	*	*	*	*	…….	*	*	*	*	8
Schalekamp et al,[4] 2008	*	*	*	*	…….	*	*	*	*	8
Dall et al,[5] 2009	*	*	*	*	*	*	*	*	*	9
Schelleman et al,[9] 2011	*	*	*	*	*	*	*	*	*	9
Baillargeon et al,[11] 2012	*	*	*	*	…….	…….	*	*	*	7
Renoux et al,[21] 2017	*	*	*	*	*	*	*	*	*	9
Gaist et al,[28] 2020	*	*	*	*	…….	*	*	*	*	8
Lee et al,[29] 2020	*	*	*	*	*	…….	*	*	*	8
Zhang et al,[32] 2020	*	*	*	*	*	*	*	*	*	9

[a]Study control for 3 of additional factors: race, alcohol use, diabetes, chronic hepatic disease, stages of kidney disease, CHF, stroke/TIA, hypertension, anemia, INR levels, NSAIDs, steroids, or PPIs/H2RAs/gastro protective agents.

Abbreviations: CHF, chronic heart failure; COPD, chronic obstructive pulmonary disease; H2RAs, histamine H2-receptor antagonists; INR, international normalized ratio; NSAIDs, nonsteroidal anti-inflammatory drugs; NOS, Newcastle-Ottawa Scale; PPIs, proton pump inhibitors; TIA, transient ischemic attack.

eTable 6. Risk of Bias Assessment of Included Studies: Using ROBINS-I[34] (Continued)

First Author, Year	ROBINS-I Domains							
	**Confounders**	**Participant Selection**	**Interventions**	**Deviation From Intended Intervention**	**Missing Data**	**Outcome Measurement**	**Reported Result**	**Overall**[†]
Kurdyak et al,[1] 2005	Low	Low	Moderate	Moderate	Low	Low	Low	Moderate
Kharofa et al,[2] 2007	Moderate	Low	Moderate	Moderate	Low	Low	Low	Moderate
de Abajo et al,[3] 2008	Moderate	Low	Moderate	Moderate	Low	Low	Low	Moderate
Schalekamp et al,[4] 2008	Moderate	Low	Moderate	Moderate	Low	Low	Low	Moderate
Dall et al,[5] 2009	Low	Low	Moderate	Moderate	Low	Low	Low	Moderate
Wallerstedt et al,[6] 2009	Serious	Moderate	Moderate	Moderate	Low	Low	Low	Serious
Cochran et al,[7] 2011	Low	Moderate	Moderate	Moderate	Low	Low	Low	Moderate
Labos et al,[8] 2011	Low	Low	Moderate	Moderate	Low	Low	Low	Moderate
Schelleman et al,[9] 2011	Low	Low	Moderate	Moderate	Low	Low	Low	Moderate
Vitry et al,[10] 2011	Moderate	Low	Moderate	Moderate	Low	Low	Low	Moderate
Baillargeon et al,[11] 2012	Moderate	Low	Moderate	Moderate	Low	Low	Low	Moderate
Lin et al,[12] 2013	Moderate	Low	Moderate	Moderate	Low	Low	Low	Moderate
Mosholder et al,[13] 2013	Moderate	Low	Moderate	Moderate	Low	Low	Low	Moderate
Seitz et al,[14] 2013	Serious	Low	Moderate	Moderate	Low	Low	Low	Serious
Giang et al,[15] 2014	No information	Moderate	Moderate	No information	No information	Moderate	No information	No information
Nguyen et al,[16] 2014	No information	Low	No information	No information	No information	No information	No information	No information
Quinn et al,[17] 2014	Serious	Low	Moderate	Moderate	Low	Low	Low	Serious
Rashid et al,[18] 2016	No information	Low	No information	No information	No information	No information	No information	No information
Lai et al,[19] 2017	No information	Low	No information	No information	No information	Moderate	No information	No information
Laursen et al,[20] 2017	Moderate	Low	Low	Low	Low	Low	Low	Moderate
Renoux et al,[21] 2017	Low	Low	Moderate	Moderate	Low	Low	Low	Moderate
Samuel et al,[22] 2017	Serious	Moderate	Moderate	Moderate	Low	Low	Low	Serious
Scheitz et al,[23] 2017	Serious	Low	Low	Moderate	Low	Low	Low	Serious
Quinn et al,[24] 2018	Low	Low	Low	Low	Low	Low	Low	Low

[†]Overall judgment includes the following categories: low risk of bias (the study is comparable to a well performed randomized trial with regard to this domain [the study is judged to have a low risk of bias for all domains]); moderate risk of bias (the study is sound for a non-randomized study with regard to this domain but cannot be considered comparable to a well performed randomized trial [the study is judged to have a low or moderate risk of bias for all domains]); serious risk of bias (the study has some important problems in this domain [the study is judged to have a low or moderate risk of bias for most domains but is at serious risk of bias in at least one domain]); critical risk of bias (the study is too problematic in this domain to provide any useful evidence [the study is judged to have a critical risk of bias in at least one domain]); and no information (no information on which to base a judgment about risk of bias for this domain [there is a lack of information in one or more key domains of bias for the outcome])
Abbreviation: ROBINS-I, risk of bias in non-randomized studies.

eTable 6. Risk of Bias Assessment of Included Studies: Using ROBINS-I[34] (Continued)

First Author, Year	ROBINS-I Domains							
	**Confounders**	**Participant Selection**	**Interventions**	**Deviation From Intended Intervention**	**Missing Data**	**Outcome Measurement**	**Reported Result**	**Overall**[†]
Iasella et al,[25] 2019	Moderate	Low	Moderate	Moderate	Low	Low	Low	Moderate
Luo et al,[26] 2019	Moderate	Low	Moderate	Moderate	Low	Low	Low	Moderate
Gaist et al,[27] 2020	Moderate	Low	Moderate	Moderate	Low	Low	Low	Moderate
Komen et al,[28] 2020	Low	Low	Moderate	Moderate	Low	Low	Low	Moderate
Lee et al,[29] 2020	Moderate	Low	Moderate	Moderate	Low	Low	Low	Moderate
Marchena et al,[30] 2020	Moderate	Low	Moderate	Moderate	Low	Low	Low	Moderate
Mawardi et al,[31] 2019	Serious	Moderate	Moderate	Moderate	Low	Low	Low	Serious
Zhang et al,[32] 2020	Low	Low	Moderate	Moderate	Low	Low	Low	Moderate

[†]Overall judgment includes the following categories: low risk of bias (the study is comparable to a well performed randomized trial with regard to this domain [the study is judged to have a low risk of bias for all domains]); moderate risk of bias (the study is sound for a non-randomized study with regard to this domain but cannot be considered comparable to a well performed randomized trial [the study is judged to have a low or moderate risk of bias for all domains]); serious risk of bias (the study has some important problems in this domain [the study is judged to have a low or moderate risk of bias for most domains but is at serious risk of bias in at least one domain]); critical risk of bias (the study is too problematic in this domain to provide any useful evidence [the study is judged to have a critical risk of bias in at least one domain]); and no information (no information on which to base a judgment about risk of bias for this domain [there is a lack of information in one or more key domains of bias for the outcome])

Abbreviation: ROBINS-I, risk of bias in non-randomized studies.

eTable 7. Quality of Evidence Synthesis and GRADE Evidence Profile of Outcomes[35]

Outcomes	No. of Studies (Ref)	Study Design (Sample Size)	Quality Assessment: Required Domains					Other Issues	Finding and Direction (Magnitude) of Effect	Strength of Evidence
			Study Limitations	Directions	Consistency	Precision	Reporting Bias			
**Major bleeding**										
Anticoagulant therapy	13 (4, 6, 7, 10, 11, 13, 17, 22, 24, 28, 29, 30, 32)	Non-RCTs (469,869)	High	Direct	Consistent	Precise	Suspected[a]	• Dose-response association could not be determined<br>• Weak strength of association (magnitude of effect)	• Thirteen non-RCTs with a large sample size revealed high study limitations and reporting bias, in which testing for publication bias indicates a substantial likelihood of bias<br>• The summary pooled OR was 1.39 (95% CI, 1.23-1.58; *P*<0.001)<br>• The findings were robust with respect to a set of sensitivity analysis	Low
Antiplatelet therapy	2 (8,18)	Non-RCTs (27,897)	High	Direct	Consistent	Imprecise	Suspected	• Dose-response association could not be determined<br>• Weak strength of association (magnitude of effect)	• One published and one unpublished non-RCTs with high study limitations and imprecise effect estimates owing to optimal information size is not met<br>• A qualitative assessment suggests the likelihood of publication bias because of the small number of studies included<br>• The summary pooled OR was 1.45 (95% CI, 1.17-1.80; *P*=0.001)	Very low

[a]Trim and fill method showed publication bias for outcome.

Abbreviations: CI, confidence interval; GRADE, Grading of Recommendations Assessment, Development and Evaluation; OR, odds ratio; RCTs, randomized-controlled trials.

eTable 7. Quality of Evidence Synthesis and GRADE Evidence Profile of Outcomes[35] (Continued)

Outcomes	No. of Studies (Ref)	Study Design (Sample Size)	Quality Assessment: Required Domains					Other Issues	Finding and Direction (Magnitude) of Effect	Strength of Evidence
			Study Limitations	Directions	Consistency	Precision	Reporting Bias			
**Brain Hemorrhage**										
Anticoagulant therapy	10 (2, 4, 13, 17, 21, 23, 27, 28, 29, 30)	Non-RCTs (443,904)	High	Direct	Inconsistent	Precise	Suspected[a]	• Dose-response association could not be determined<br>• Weak strength of association (magnitude of effect)	• Ten non-RCTs with high study limitations, inconsistency, and reporting bias<br>• The summary pooled OR was 1.31 (95% CI, 1.02-1.68; *P*=0.031), however, no longer association was observed after calibration for the publication bias by the Trim and fill method	Very low
Antiplatelet therapy	3 (2, 21, 27)	Non-RCTs (81,173)	High	Indirect	Inconsistent	Imprecise	Suspected	• Dose-response association could not be determined<br>• Weak strength of association (magnitude of effect)	• Three non-RCTs with high study limitations, indirectness, and imprecision<br>• Publication bias cannot be ruled out owing to the small number of studies included<br>• According to the sensitivity analysis, the result was affected by de Abajo et al,[3] 2008. The pooled OR estimate was 1.08 (95% CI, 0.93-1.26)	Very low

[a]Trim and fill method showed publication bias for outcome.

Abbreviations: CI, confidence interval; GRADE, Grading of Recommendations Assessment, Development and Evaluation; OR, odds ratio; RCTs, randomized-controlled trials.

eTable 7. Quality of Evidence Synthesis and GRADE Evidence Profile of Outcomes[35] (Continued)

Outcomes	No. of Studies (Ref)	Study Design (Sample Size)	Quality Assessment: Required Domains					Other Issues	Finding and Direction (Magnitude) of Effect	Strength of Evidence
			Study Limitations	Directions	Consistency	Precision	Reporting Bias			
**GI Bleeding**										
Anticoagulant therapy	10 (1, 3, 4, 9, 13, 19, 28, 29, 31, 32)	Non-RCTs (1,085,014)	High	Direct	Consistent	Precise	Suspected[a]	• Dose-response association could not be determined<br>• Weak strength of association (magnitude of effect)	• Ten non-RCTs with a large sample size revealed high study limitations<br>• The summary pooled OR was 1.32 (95% CI, 1.17-1.49; *P*<0.001), and results were robust with respect to a set of sensitivity analysis	Low
Antiplatelet therapy	5 (3, 5, 8, 12, 26)	Non-RCTs (52,571)	High	Direct	Consistent	Precise	Suspected[a]	• Dose-response association could not be determined<br>• Weak strength of association (magnitude of effect)	• Five non-RCTs with high study limitations and suspected of publication bias<br>• The summary OR was 1.30 (95% CI, 1.04-1.63; *P*=0.021), however, no longer association was observed after calibration for the publication bias by the Trim and fill method	Very low

[a]Trim and fill method showed publication bias for outcome.

Abbreviations: CI, confidence interval; GI, gastrointestinal; GRADE, Grading of Recommendations Assessment, Development and Evaluation; OR, odds ratio; RCTs, randomized-controlled trials.

eTable 7. Quality of Evidence Synthesis and GRADE Evidence Profile of Outcomes[35] (Continued)

Outcomes	No. of Studies (Ref)	Study Design (Sample Size)	Quality Assessment: Required Domains					Other Issues	Finding and Direction (Magnitude) of Effect	Strength of Evidence
			Study Limitations	Directions	Consistency	Precision	Reporting Bias			
**Any Bleeding**										
Anticoagulant therapy	23 (1, 2, 3, 4, 6, 7, 9, 10, 11, 13, 16, 17, 19, 21, 22, 23, 24, 27, 28, 29, 30, 31, 32)	Non-RCTs (1,209,421)	High	Direct	Consistent	Precise	Suspected[a]	• Dose-response association could not be determined<br>• Weak strength of association (magnitude of effect)	• Twenty-three non-RCTs with a large sample size revealed high study limitations and suspected publication bias<br>• The pooled OR estimate was 1.39 (95% CI, 1.24-1.55; *P*<0.001), and results were robust with respect to a set of sensitivity analysis<br>• The direction and magnitude may be affected by the definitions of bleeding events	Low
Antiplatelet therapy	11 (2, 3, 5, 8, 12, 15, 18, 21, 25, 26, 27)	Non-RCTs (153,790)	High	Direct	Consistent	Precise	Suspected[a]	• Dose-response association could not be determined<br>• Weak strength of association (magnitude of effect)	• Eleven non-RCTs with high study limitations and suspected of publication bias<br>• The summary OR was 1.15 (95% CI, 1.06-1.25; *P*=0.001)<br>• The direction and magnitude may be affected by the definitions of exposure and bleeding outcomes	Low

[a]Trim and fill method showed publication bias for outcome.

Abbreviations: CI, confidence interval; GRADE, Grading of Recommendations Assessment, Development and Evaluation; OR, odds ratio; RCTs, randomized-controlled trials.

eTable 7. Quality of Evidence Synthesis and GRADE Evidence Profile of Outcomes[35] (Continued)

Outcomes	No. of Studies (Ref)	Study Design (Sample Size)	Quality Assessment: Required Domains					Other Issues	Finding and Direction (Magnitude) of Effect	Strength of Evidence
			Study Limitations	Directions	Consistency	Precision	Reporting Bias			
**Blood Transfusion**										
Anticoagulant therapy	1 (14)	Non-RCTs (516)	High	Indirect	Unknown	Precise	Suspected	• Dose-response association could not be determined<br>• Present plausible confounding that would decrease the observed effect	• A single study with high study limitations and indirectness of effect estimates by Seitz et al[14] revealed statistical insignificance (OR 1.55; 95% CI, 0.98-2.46)	NA (Insufficient)
Antiplatelet therapy	1 (14)	Non-RCTs (280)	High	Indirect	Unknown	Imprecise	Suspected	• Dose-response association could not be determined<br>• Present plausible confounding that would decrease the observed effect	• A single study with high study limitations and indirectness of effect estimates by Seitz et al[14] revealed statistical significance (OR 2.10; 95% CI, 1.02-4.33), but the evidence sample size did not meet optimal information size	NA (Insufficient)

[a]Trim and fill method showed publication bias for outcome.

Abbreviations: CI, confidence interval; GRADE, Grading of Recommendations Assessment, Development and Evaluation; NA, not applicable; OR, odds ratio; RCTs, randomized-controlled trials.

eTable 7. Quality of Evidence Synthesis and GRADE Evidence Profile of Outcomes[35] (Continued)

Outcomes	No. of Studies (Ref)	Study Design (Sample Size)	Quality Assessment: Required Domains					Other Issues	Finding and Direction (Magnitude) of Effect	Strength of Evidence
			Study Limitations	Directions	Consistency	Precision	Reporting Bias			
**Endoscopy-Refractory Bleeding**										
Antiplatelet therapy	1 (20)	Non-RCTs (4,316)	High	Indirect	Unknown	Precise	Suspected	• Dose-response association could not be determined<br>• Present plausible confounding that would decrease the observed effect	• A single study with high limitations and indirectness of effect estimates by Laursen et al[20] revealed statistical insignificance (OR 0.95; 95% CI, 0.60-1.51)	NA (Insufficient)
**Rebleeding**										
Antiplatelet therapy	1 (20)	Non-RCTs (4,344)	High	Indirect	Unknown	Precise	Suspected	• Dose-response association could not be determined<br>• Present plausible confounding that would decrease the observed effect	• A single study with high limitations and indirectness of effect estimates by Laursen et al[20] revealed statistical insignificance (OR 1.02; 95% CI, 0.80-1.31)	NA (Insufficient)

[a]Trim and fill method showed publication bias for outcome.

Abbreviations: CI, confidence interval; GRADE, Grading of Recommendations Assessment, Development and Evaluation; OR, odds ratio; RCTs, randomized-controlled trials.

eTable 8. Summary of Additional Secondary Outcomes

Bleeding Complication	No. of Studies Included (Ref)	No. of Participants	Odds Ratio (95% CI)	*P* Value	Heterogeneity			
					Q Statistic	*P* Value	I^2 Index (95% CI)	τ^2
**Blood Transfusion**								
Anticoagulant therapy	1 (14)	516	1.55 (0.98-2.46)	0.063	NA	NA	NA	NA
Antiplatelet Therapy	1 (14)	280	2.10 (1.02-4.33)	0.044	NA	NA	NA	NA
**Endoscopy-Refractory Bleeding**								
Antiplatelet Therapy	1 (20)	4,316	0.95 (0.60-1.51)	0.828	NA	NA	NA	NA
**Rebleeding**								
Antiplatelet Therapy	1 (20)	4,344	1.02 (0.80-1.31)	0.876	NA	NA	NA	NA

Abbreviations: CI, confidence interval; NA, not applicable.

eTable 9. Subgroup Analysis: Major Bleeding

Subgroup Comparison	No. of Studies (Ref)	No. of Participants	Odds Ratio (95% CI)	*P* Value	Heterogeneity			
					Q Statistic	*P* Value	I^2 Index (95% CI)	τ^2
**Anticoagulant Therapy**								
**Sample Size**								
<5,000	6 (6, 7, 11, 22, 24, 32)	7,462	1.39 (1.15-1.69)	0.001	7.56	0.272	20.6% (0.0-66.5)	0.013
≥5,000	7 (4, 10, 13, 17, 28, 29, 30)	462,407	1.39 (1.17-1.64)	<0.001	22.74	0.002	69.2% (16.9-83.5)	0.035
**Study Design**								
Cohort	4 (4, 11, 29,32)	38,638	1.28 (1.13-1.45)	<0.001	14.65	0.145	31.7% (0.0-66.4)	0.0112
Case-control	9 (6, 7, 10, 13, 17, 22, 24, 28, 30)	431,231	1.54 (1.25-1.91)	<0.001	6.57	0.087	54.3% (0.0-83.0)	0.024
**Study Location**								
North America	5 (7, 11, 13, 17, 22)	337,409	1.23 (1.06-1.43)	0.006	5.97	0.201	33.0% (0.0-74.8)	0.009
Non-North America	8 (4, 6, 10, 24, 28, 29, 30, 32)	132,460	1.46 (1.25-1.71)	<0.001	16.50	0.057	45.5% (0.0-72.2)	0.026
**Antiplatelet Therapy**								
**Sample Size**								
<5,000	2 (8, 18)	24,546	1.29 (0.65-2.57)	0.464	0.79	0.374	0.0% (NA)	<0.001
≥5,000	1 (8)	3,351	1.47 (1.17-1.84)	0.001	0.17	0.678	0.0% (NA)	<0.001
**Study Design**								
Cohort	2 (8, 18)	27,897	1.45 (1.17-1.80)	0.001	1.08	0.782	0.0% (0.0-67.9)	<0.001
Case-control	NA	NA	NA	NA	NA	NA	NA	NA
**Study Location**								
North America	1 (8)	27,058	1.47 (1.19-1.83)	<0.001	0.19	0.912	0.0% (0.0-72.9)	<0.001
Non-North America	1 (18)	839	0.74 (0.18-3.02)	0.676	NA	NA	NA	NA

Abbreviations: CI, confidence interval; NA, not applicable.

eTable 9. Subgroup Analysis: Brain Hemorrhage (Continued)

Subgroup Comparison	No. of Studies (Ref)	No. of Participants	Odds Ratio (95% CI)	*P* Value	Heterogeneity			
					Q Statistic	*P* Value	I^2 Index (95% CI)	τ^2
**Anticoagulant Therapy**								
**Sample Size**								
<5,000	5 (2, 21, 23, 27, 29)	10,459	2.16 (0.89-5.29)	0.090	21.70	<0.001	81.6% (45.0-90.4)	0.787
≥5,000	6 (4, 13, 17, 27, 28, 30)	433,445	1.13 (0.97-1.31)	0.123	6.74	0.345	11.0% (0.0-62.9)	0.005
**Study Design**								
Cohort	5 (13, 17, 23, 28, 30)	411,420	1.36 (1.03-1.80)	0.032	7.60	0.180	34.2% (0.0-73.1)	0.040
Case-control	5 (2, 4, 21, 27, 29)	32,484	1.30 (0.87-1.95)	0.202	15.87	0.007	68.5% (0.0-84.7)	0.142
**Study Location**								
North America	3 (2, 13, 17)	336,234	1.29 (1.00-1.68)	0.054	0.33	0.849	0.0% (0.0-72.9)	<0.001
Non-North America	7 (4, 21, 23, 27, 28, 29, 30)	107,670	1.36 (0.97-1.92)	0.074	26.24	0.001	69.5% (24.6-83.1)	0.150
**Antiplatelet Therapy**								
**Sample Size**								
<5,000	1 (2)	2,692	0.82 (0.60-1.11)	0.202	NA	NA	NA	NA
≥5,000	2 (21, 27)	78,481	1.16 (1.04-1.29)	0.006	3.42	0.181	41.4% (NA)	0.004
**Study Design**								
Cohort	NA	NA	NA	NA	NA	NA	NA	NA
Case-control	3 (2, 21, 27)	81,173	1.08 (0.93-1.26)	0.325	8.98	0.030	66.6% (0.0-86.4)	0.014
**Study Location**								
North America	1 (2)	2,692	0.82 (0.60-1.11)	0.202	NA	NA	NA	NA
Non-North America	2 (21, 27)	78,481	1.16 (1.04-1.29)	0.006	3.42	0.181	41.4% (NA)	0.004

Abbreviations: CI, confidence interval; NA, not applicable.

eTable 9. Subgroup Analysis: GI Bleeding (Continued)

Subgroup Comparison	No. of Studies (Ref)	No. of Participants	Odds Ratio (95% CI)	*P* Value	Heterogeneity			
					Q Statistic	*P* Value	I^2 Index (95% CI)	τ^2
**Anticoagulant Therapy**								
**Sample Size**								
<5,000	3 (4, 31, 32)	3,518	1.24 (0.78-1.99)	0.367	3.26	0.196	38.6% (0.0-82.1)	0.067
≥5,000	7 (1, 3, 9, 13, 19, 28, 29)	1,081,496	1.34 (1.19-1.51)	<0.001	12.26	0.092	42.9% (0.0-73.2)	0.010
**Study Design**								
Cohort	4 (13, 19, 28, 31)	376,702	1.40 (1.16-1.67)	<0.001	7.15	0.128	44.1% (0.0-78.1)	0.017
Case-control	6 (1, 3, 4, 9, 29, 32)	708,312	1.29 (1.08-1.55)	0.006	8.36	0.137	40.2% (0.0-75.0)	0.017
**Study Location**								
North America	5 (1, 9, 13, 19, 31)	1,029,076	1.34 (1.16-1.55)	<0.001	11.39	0.023	64.9% (0.0-84.5)	0.016
Non-North America	5 (3, 4, 28, 29, 32)	55,938	1.36 (1.08-1.71)	0.009	4.11	0.533	0.0% (0.0-61.0)	<0.001
**Antiplatelet Therapy**								
**Sample Size**								
<5,000	1 (12)	3,238	1.72 (0.99-3.00)	0.056	NA	NA	NA	NA
≥5,000	4 (3, 5, 8, 26)	49,333	1.23 (0.99-1.53)	0.061	4.07	0.254	26.3% (0.0-75.6)	0.015
**Study Design**								
Cohort	3 (8, 12, 26)	29,175	1.59 (1.20-2.10)	0.001	0.25	0.881	0.0% (0.0-72.9)	<0.001
Case-control	2 (3, 5)	23,396	1.09 (0.94-1.26)	0.278	0.12	0.726	0.0% (NA)	<0.001
**Study Location**								
North America	1 (8)	14,832	1.50 (1.05-2.15)	0.027	NA	NA	NA	NA
Non-North America	4 (3, 5, 12, 26)	37,739	1.24 (0.96-1.60)	0.095	3.94	0.268	23.9% (0.0-74.9)	0.021

Abbreviations: CI, confidence interval; GI, gastrointestinal; NA, not applicable.

eTable 9. Subgroup Analysis: Any Bleeding (Continued)

Subgroup Comparison	No. of Studies (Ref)	No. of Participants	Odds Ratio (95% CI)	*P* Value	Heterogeneity			
					Q Statistic	*P* Value	I^2 Index (95% CI)	τ^2
**Anticoagulant Therapy**								
**Sample Size**								
<5,000	11 (2, 6, 7, 11, 16, 21, 22, 23, 24, 31, 32)	16,629	1.59 (1.25-2.02)	<0.001	34.15	0.001	64.9% (25.7-79.1)	0.098
≥5,000	12 (1, 3, 4, 9, 10, 13, 17, 19, 27, 28, 29, 30)	1,192,792	1.33 (1.18-1.50)	<0.001	42.66	<0.001	71.9% (44.8-82.6)	0.030
**Study Design**								
Cohort	13 (6, 7, 10, 13, 16, 17, 19, 22, 23, 24, 28, 30, 31)	456,368	1.48 (1.27-1.72)	<0.001	37.40	0.001	62.6% (24.6-77.2)	0.041
Case-control	10 (1, 2, 3, 4, 9, 11, 21, 27, 29, 32)	753,053	1.29 (1.09-1.54)	0.004	41.63	<0.001	76.0% (51.8-85.3)	0.053
**Study Location**								
North America	11 (1, 2, 7, 9, 11, 13, 16, 17, 19, 22, 31)	1,047,974	1.39 (1.22-1.59)	<0.001	27.71	0.002	63.9% (15.5-79.5)	0.023
Non-North America	12 (3, 4, 6, 10, 21, 23, 24, 27, 28, 29, 30, 32)	161,447	1.40 (1.15-1.69)	0.001	51.16	<0.001	72.6% (49.9-82.4)	0.083
**Antiplatelet Therapy**								
**Sample Size**								
<5,000	5 (2, 8, 12, 15, 18)	9,443	1.10 (0.82-1.48)	0.510	6.81	0.146	41.3% (0.0-77.2)	0.044
≥5,000	6 (3, 5, 21, 25, 26, 27)	144,347	1.17 (1.08-1.26)	<0.001	11.59	0.170	31.0% (0.0-67.4)	0.004
**Study Design**								
Cohort	6 (8, 12, 15, 18, 25, 26)	49,221	1.27 (1.08-1.50)	0.004	9.87	0.196	29.1% (0.0-68.0)	0.014
Case-control	5 (2, 3, 5, 21, 27)	104,569	1.10 (0.99-1.22)	0.088	10.03	0.074	50.2% (0.0-78.3)	0.008
**Study Location**								
North America	4 (2, 8, 15, 25)	36,731	1.18 (0.96-1.45)	0.108	10.91	0.053	54.2% (0.0-79.7)	0.031
Non-North America	7 (3, 8, 15, 18, 21, 26, 27)	117,059	1.16 (1.07-1.26)	<0.001	8.70	0.275	19.5% (0.0-64.3)	0.002

Abbreviations: CI, confidence interval; NA, not applicable.

eTable 10. Sensitivity Analysis: Restricted the Analysis to Studies that Adjustment for Key Determinants (Age, Sex, and History of Bleeding)

Bleeding Complication	No. of Studies (Ref)	No. of Participants	Odds Ratio (95% CI)	*P* Value	Heterogeneity			
					Q Statistic	*P* Value	I^2 Index (95% CI)	τ^2
**Major Bleeding**								
Anticoagulant therapy	7 (7, 10, 24, 28, 29, 30, 32)	124,660	1.44 (1.22-1.70)	<0.001	13.77	0.088	41.9% (0.0-71.7)	0.023
Antiplatelet therapy	1 (8)	27,058	1.47 (1.19-1.83)	0.001	0.19	0.912	0.0% (0.0-72.9)	<0.001
**Brain Hemorrhage**								
Anticoagulant therapy	4 (21, 28, 29, 30)	82,918	1.58 (1.06-2.34)	0.024	6.02	0.198	33.6% (0.0-74.9)	0.067
Antiplatelet therapy	1 (21)	5,732	0.94 (0.65-1.35)	0.737	NA	NA	NA	NA
**GI Bleeding**								
Anticoagulant therapy	5 (1, 9, 28, 29, 32)	725,067	1.35 (1.18-1.55)	<0.001	6.31	0.277	20.8% (0.0-68.6)	0.006
Antiplatelet therapy	2 (5, 8)	26,907	1.46 (1.05-2.02)	0.025	0.17	0.684	0.0% (NA)	<0.001
**Any Bleeding**								
Anticoagulant therapy	10 (1, 7, 9, 10, 21, 24, 28, 29, 30, 32)	808,209	1.43 (1.25-1.65)	<0.001	24.43	0.011	55.0% (0.0-74.9)	0.026
Antiplatelet therapy	3 (5, 8, 21)	44,865	1.30 (1.07-1.59)	0.010	4.58	0.333	12.7% (0.0-68.4)	0.007

Abbreviations: CI, confidence interval, NA, not applicable.

eTable 11. Sensitivity Analysis: Restricted the Analysis to the Highest-Quality Study (NOS ≥8 Points)

Bleeding Complication	No. of Studies (Ref)	No. of Participants	Odds Ratio (95% CI)	*P* Value	Heterogeneity			
					Q Statistic	*P* Value	I^2 Index (95% CI)	τ^2
**Major Bleeding**								
Anticoagulant therapy	8 (4, 7, 10, 13, 24, 28, 29, 32)	409,632	1.41 (1.18-1.69)	<0.001	24.91	0.003	63.9% (10.2-80.0)	0.041
Antiplatelet therapy	1 (8)	27,058	1.47 (1.19-1.83)	0.001	0.19	0.912	0.0% (0.0-72.9)	<0.001
**Brain Hemorrhage**								
Anticoagulant therapy	7 (2, 4, 13, 21, 27, 28, 29)	387,435	1.17 (0.91-1.51)	0.217	17.20	0.028	53.5% (0.0-76.3)	0.063
Antiplatelet therapy	3 (2, 21, 27)	81,173	1.08 (0.93-1.26)	0.325	8.98	0.030	66.6% (0.0-86.4)	0.014
**GI Bleeding**								
Anticoagulant therapy	8 (1, 3, 4, 9, 13, 28, 29, 32)	1,085,014	1.28 (1.14-1.43)	<0.001	11.08	0.197	27.8% (0.0-66.1)	0.007
Antiplatelet therapy	5 (3, 5, 8, 12, 26)	52,571	1.30 (1.04-1.63)	0.021	5.93	0.204	32.6% (0.0-74.6)	0.021
**Any Bleeding**								
Anticoagulant therapy	14 (1, 2, 3, 4, 7, 9, 10, 13, 21, 24, 27, 28, 29, 32)	1,124,047	1.31 (1.15-1.50)	<0.001	55.74	<0.001	71.3% (49.2-81.3)	0.042
Antiplatelet therapy	8 (2, 3, 5, 8, 12, 21, 26, 27)	145,970	1.18 (1.06-1.30)	0.002	17.46	0.065	42.7% (0.0-70.2)	0.009

Abbreviations: CI, confidence interval; NA, not applicable; NOS, Newcastle-Ottawa scale.

eTable 12. Sensitivity Analysis: Restricted the Analysis to Studies with the Directness of Effect Estimates

Bleeding Complication	No. of Studies (Ref)	No. of Participants	Odds Ratio (95% CI)	*P* Value	Heterogeneity			
					Q Statistic	*P* Value	I^2 Index (95% CI)	τ^2
**Major Bleeding**								
Anticoagulant therapy	10 (4, 6, 7, 17, 22, 24, 28, 29, 30, 32)	124,660	1.45 (1.27-1.76)	<0.001	17.19	0.102	36.0% (0.0-66.5)	0.019
Antiplatelet therapy	2 (8, 18)	27,897	1.45 (1.17-1.80)	0.001	1.08	0.782	0.0% (0.0-67.9)	<0.001
**Brain Hemorrhage**								
Anticoagulant therapy	5 (4, 17, 28, 29, 30)	99,190	1.46 (1.13-1.87)	0.003	4.86	0.433	0.0% (0.0-61.0)	<0.001
Antiplatelet therapy	NA	NA	NA	NA	NA	NA	NA	NA
**GI Bleeding**								
Anticoagulant therapy	7 (1, 4, 9, 28, 29, 31, 32)	727,834	1.35 (1.16-1.55)	<0.001	9.66	0.209	27.5% (0.0-67.4)	0.011
Antiplatelet therapy	1 (8)	14,832	1.50 (1.05-2.15)	0.027	NA	NA	NA	NA
**Any Bleeding**								
Anticoagulant therapy	14 (1, 4, 6, 7, 9, 16, 17, 22, 24, 28, 29, 30, 31, 32)	811,030	1.46 (1.30-1.63)	<0.001	28.78	0.017	47.9% (0.0-69.5)	0.021
Antiplatelet therapy	4 (8, 15, 18, 25)	34,878	1.21 (1.03-1.43)	0.021	6.99	0.221	28.5% (0.0-71.2)	0.012

Abbreviations: CI, confidence interval; NA, not applicable.

eTable 13. Sensitivity Analysis: Removing Unpublished Studies

Bleeding Complication	No. of Studies (Ref)	No. of Participants	Odds Ratio (95% CI)	*P* Value	Heterogeneity			
					Q Statistic	*P* Value	I^2 Index (95% CI)	τ^2
**Major Bleeding**								
Anticoagulant therapy	13 (4, 6, 7, 10, 11, 13, 17, 22, 24, 28, 29, 30, 32)	469,869	1.39 (1.23-1.58)	<0.001	31.27	0.005	55.2% (4.7-73.6)	0.026
Antiplatelet therapy	1 (8)	27,058	1.47 (1.19-1.83)	0.001	0.19	0.912	0.0% (0.0-72.9)	<0.001
**Brain Hemorrhage**								
Anticoagulant therapy	10 (2, 4, 13, 17, 21, 23, 27, 28, 29, 30)	443,904	1.31 (1.02-1.68)	0.031	28.48	0.003	61.4% (11.8-77.9)	0.091
Antiplatelet therapy	3 (2, 21, 27)	81,173	1.08 (0.93-1.26)	0.325	8.98	0.030	66.6% (0.0-86.4)	0.014
**GI Bleeding**								
Anticoagulant therapy	9 (1, 3, 4, 9, 13, 28, 29, 31, 32)	1,063,511	1.29 (1.16-1.45)	<0.001	12.36	0.194	27.2% (0.0-64.6)	0.007
Antiplatelet therapy	5 (3, 5, 8, 12, 26)	52,571	1.30 (1.04-1.63)	0.021	5.93	0.204	32.6% (0.0-74.6)	0.021
**Any Bleeding**								
Anticoagulant therapy	21 (1, 2, 3, 4, 6, 7, 9, 10, 11, 13, 17, 21, 22, 23, 24, 27, 28, 29, 30, 31, 32)	1,184,765	1.35 (1.21-1.52)	<0.001	70.55	<0.001	67.4% (46.8-77.7)	0.039
Antiplatelet therapy	9 (2, 3, 5, 8, 12, 21, 25, 26, 27)	152,789	1.16 (1.06-1.27)	0.001	19.17	0.058	42.6% (0.0-69.4)	0.008

Abbreviations: CI, confidence interval; NA, not applicable.

eTable 14. Sensitivity Analysis: Outcomes After Removing Individuals Studies

First Author, Year	OR (95% CI): Major Bleeding		OR (95% CI): Brain Hemorrhage	
	**Anticoagulant**	**Antiplatelet**	**Anticoagulant**	**Antiplatelet**
All studies	1.39 (1.23-1.58)	1.45 (1.17-1.80)	1.31 (1.02-1.68)	1.08 (0.93-1.26)
Kurdyak et al,[1] 2005	NA	NA	NA	NA
Kharofa et al,[2] 2007	NA	NA	1.30 (1.01-1.68)	1.16 (1.04-1.29)
de Abajo et al,[3] 2008	NA	NA	NA	NA
Schalekamp et al,[4] 2008	1.40 (1.22-1.60)	NA	1.30 (1.00-1.68)	NA
Dall et al,[5] 2009	NA	NA	NA	NA
Wallerstedt et al,[6] 2009	1.36 (1.21-1.54)	NA	NA	NA
Cochran et al,[7] 2011	1.39 (1.22-1.58)	NA	NA	NA
Labos et al,[8] 2011 (Aspirin cohort)	NA	1.50 (1.07-2.10)	NA	NA
Labos et al,[8] 2011 (Clopidogrel cohort)	NA	1.44 (1.16-1.80)	NA	NA
Labos et al,[8] 2011 (DAPT cohort)	NA	1.40 (1.09-1.81)	NA	NA
Schelleman et al,[9] 2011	NA	NA	NA	NA
Vitry et al,[10] 2011	1.38 (1.22-1.57)	NA	NA	NA
Baillargeon et al,[11] 2012	1.41 (1.22-1.63)	NA	NA	NA
Lin et al,[12] 2013	NA	NA	NA	NA
Mosholder et al,[13] 2013	1.43 (1.27-1.61)	NA	1.35 (1.02-1.80)	NA
Seitz et al,[14] 2013	NA	NA	NA	NA
Giang et al,[15] 2014	NA	NA	NA	NA
Nguyen et al,[16] 2014	NA	NA	NA	NA
Quinn et al,[17] 2014	1.39 (1.22-1.60)	NA	1.32 (1.01-1.72)	NA
Rashid et al,[18] 2016	NA	1.47 (1.19-1.83)	NA	NA
Lai et al,[19] 2017	NA	NA	NA	NA
Laursen et al,[20] 2017	NA	NA	NA	NA
Renoux et al,[21] 2017	NA	NA	1.27 (0.99-1.62)	1.10 (0.93-1.30)
Samuel et al,[22] 2017	1.39 (1.22-1.57)	NA	NA	NA
Scheitz et al,[23] 2017	NA	NA	1.23 (0.98-1.53)	NA
Quinn et al,[24] 2018 (DOACs cohort)	1.41 (1.23-1.60)	NA	NA	NA
Quinn et al,[24] 2018 (VKAs cohort)	1.42 (1.23-1.63)	NA	NA	NA
Iasella et al,[25] 2019	NA	NA	NA	NA
Luo et al,[26] 2019	NA	NA	NA	NA
Gaist et al,[27] 2020 (VKAs cohort)	NA	NA	1.42 (1.06-1.90)	NA
Gaist et al,[27] 2020 (DOACs cohort)	NA	NA	1.42 (1.10-1.82)	NA
Gaist et al,[27] 2020 (Aspirin cohort)	NA	NA	NA	0.99 (0.82-1.20)
Gaist et al,[27] 2020 (Clopidogrel cohort)	NA	NA	NA	1.02 (0.76-1.35)
Komen et al,[28] 2020 (VKAs cohort)	1.40 (1.23-1.61)	NA	1.35 (1.03-1.76)	NA
Komen et al,[28] 2020 (DOACs cohort)	1.38 (1.21-1.57)	NA	1.34 (1.03-1.74)	NA
Lee et al,[29] 2020	1.30 (1.17-1.44)	NA	1.22 (0.97-1.55)	NA
Marchena et al,[30] 2020	1.43 (1.24-1.64)	NA	1.28 (0.99-1.67)	NA
Mawardi et al,[31] 2019	NA	NA	NA	NA
Zhang et al,[32] 2020	1.38 (1.21-1.57)	NA	NA	NA

Abbreviations: CI, confidence interval; DAPT, dual antiplatelet therapy; DOACs, direct oral anticoagulants; NA, not applicable; OR, odds ratio; VKAs, vitamin K antagonists.

eTable 14. Sensitivity Analysis: Outcomes After Removing Individuals Studies (Continued)

First Author, Year	OR (95% CI): GI Bleeding		OR (95% CI): Any Bleeding	
	Anticoagulant	Antiplatelet	Anticoagulant	Antiplatelet
All studies	1.34 (1.19-1.50)	1.30 (1.04-1.63)	1.39 (1.24-1.55)	1.15 (1.06-1.25)
Kurdyak et al,[1] 2005	1.38 (1.23-1.55)	NA	1.41 (1.25-1.58)	NA
Kharofa et al,[2] 2007	NA	NA	1.38 (1.24-1.55)	1.17 (1.091.26)
de Abajo et al,[3] 2008	1.34 (1.19-1.51)	1.55 (1.19-2.02)	1.39 (1.24-1.55)	1.16 (1.06-1.28)
Schalekamp et al,[4] 2008	1.35 (1.21-1.51)	NA	1.39 (1.24-1.55)	NA
Dall et al,[5] 2009	NA	1.34 (1.02-1.76)	NA	1.15 (1.05-1.25)
Wallerstedt et al,[6] 2009	NA	NA	1.37 (1.23-1.52)	NA
Cochran et al,[7] 2011	NA	NA	1.37 (1.23-1.53)	NA
Labos et al,[8] 2011 (Aspirin cohort)	NA	1.24 (0.96-1.60)	NA	1.13 (1.04-1.23)
Labos et al,[8] 2011 (Clopidogrel cohort)	NA	NA	NA	1.15 (1.05-1.25)
Labos et al,[8] 2011 (DAPT cohort)	NA	NA	NA	1.14 (1.05-1.23)
Schelleman et al,[9] 2011	1.31 (1.15-1.48)	NA	1.38 (1.23-1.56)	NA
Vitry et al,[10] 2011	NA	NA	1.38 (1.23-1.54)	NA
Baillargeon et al,[11] 2012	NA	NA	1.40 (1.24-1.57)	NA
Lin et al,[12] 2013	NA	1.23 (0.99-1.53)	NA	1.14 (1.05-1.24)
Mosholder et al,[13] 2013	1.38 (1.23-1.56)	NA	1.41 (1.26-1.59)	NA
Seitz et al,[14] 2013	NA	NA	NA	NA
Giang et al,[15] 2014	NA	NA	NA	1.15 (1.05-1.25)
Nguyen et al,[16] 2014	NA	NA	1.37 (1.22-1.53)	NA
Quinn et al,[17] 2014	NA	NA	1.39 (1.24-1.55)	NA
Rashid et al,[18] 2016	NA	NA	NA	1.16 (1.06-1.26)
Lai et al,[19] 2017	1.29 (1.16-1.45)	NA	1.37 (1.23-1.54)	NA
Laursen et al,[20] 2017	NA	NA	NA	NA
Renoux et al,[21] 2017	NA	NA	1.38 (1.23-1.54)	1.16 (1.06-1.26)
Samuel et al,[22] 2017	NA	NA	1.38 (1.24-1.54)	NA
Scheitz et al,[23] 2017	NA	NA	1.37 (1.23-1.52)	NA
Quinn et al,[24] 2018 (DOACs cohort)	NA	NA	1.40 (1.25-1.57)	NA
Quinn et al,[24] 2018 (VKAs cohort)	NA	NA	1.38 (1.23-1.54)	NA
Iasella et al,[25] 2019	NA	NA	NA	1.16 (1.06-1.28)
Luo et al,[26] 2019	NA	1.27 (1.00-1.61)	NA	1.14 (1.05-1.24)
Gaist et al,[27] 2020 (VKAs cohort)	NA	NA	1.42 (1.27-1.58)	NA
Gaist et al,[27] 2020 (DOACs cohort)	NA	NA	1.42 (1.27-1.58)	NA
Gaist et al,[27] 2020 (Aspirin cohort)	NA	NA	NA	1.13 (1.02-1.24)
Gaist et al,[27] 2020 (Clopidogrel cohort)	NA	NA	NA	1.16 (1.05-1.28)
Komen et al,[28] 2020 (VKAs cohort)	1.33 (1.18-1.49)	NA	1.39 (1.24-1.56)	NA
Komen et al,[28] 2020 (DOACs cohort)	1.34 (1.18-1.51)	NA	1.38 (1.23-1.54)	NA
Lee et al,[29] 2020	1.32 (1.18-1.48)	NA	1.35 (1.21-1.50)	NA
Marchena et al,[30] 2020	NA	NA	1.40 (1.25-1.58)	NA
Mawardi et al,[31] 2019	1.32 (1.18-1.48)	NA	1.38 (1.23-1.54)	NA
Zhang et al,[32] 2020	1.34 (1.19-1.51)	NA	1.38 (1.23-1.54)	NA

Abbreviations: CI, confidence interval; DAPT, dual antiplatelet therapy; DOACs, direct oral anticoagulants; GI, gastrointestinal; NA not applicable; OR, odds ratio; VKAs, vitamin K antagonists.

eTable 15. Sensitivity Analysis: Using Fixed-Effects Models

Bleeding Complication	No. of Studies (Ref)	No. of Participants	Odds Ratio (95% CI)		*P* Value	Heterogeneity		
			Random Effect Model	Fixed Effect Model		*Q* Statistic	*P* Value	I^2 Index (95% CI)
**Major Bleeding**								
Anticoagulation therapy	13 (4, 6, 7, 10, 11, 13, 17, 22, 24, 28, 29, 30, 32)	469869	1.39 (1.23-1.58)	Not estimated	<0.001	31.27	0.005	55.2% (4.7-73.6)
Antiplatelet therapy	2 (8, 18)	27897	1.45 (1.17-1.80)	1.45 (1.17-1.80)	0.001	1.08	0.782	0.0% (0.0-67.9)
**Brain Hemorrhage**								
Anticoagulation therapy	10 (2, 4, 13, 17, 21, 23, 27, 28, 29, 30)	443904	1.31 (1.02-1.68)	Not estimated	0.031	28.48	0.003	61.4% (11.8-77.9)
Antiplatelet therapy	3 (2, 21, 27)	81173	1.08 (0.93-1.26)	Not estimated	0.325	8.98	0.030	66.6% (0.0-86.4)
**GI Bleeding**								
Anticoagulation therapy	10 (1, 3, 4, 9, 13, 19, 28, 29, 31, 32)	1085014	1.34 (1.19-1.50)	Not estimated	<0.001	15.57	0.113	35.8% (0.0-67.2)
Antiplatelet therapy	5 (3, 5, 8, 12, 26)	52571	1.30 (1.04-1.63)	Not estimated	0.021	5.93	0.204	32.6% (0.0-74.6)
**Any Bleeding**								
Anticoagulation therapy	23 (1, 2, 3, 4, 6, 7, 9, 10, 11, 13, 16, 17, 19, 21, 22, 23, 24, 27, 28, 29, 30, 31, 32)	1209421	1.39 (1.24-1.55)	Not estimated	<0.001	79.03	<0.001	68.4% (49.8-78.0)
Antiplatelet therapy	11 (2, 3, 5, 8, 12, 15, 18, 21, 25, 26, 27)	153790	1.15 (1.06-1.25)	Not estimated	0.001	20.08	0.093	35.3% (0.0-64.6)

Abbreviation: CI, confidence interval; GI, gastrointestinal.

eTable 16. Meta-Regression of Included Studies: Major Bleeding

Covariate	Anticoagulant			Antiplatelet		
	No. of Studies (Ref.)	Odd Ratio (95% CI)[a]	*P* Value	No. of Studies (Ref.)	Odd Ratio (95% CI)[a]	*P* Value
**Risk of Bias Assessment**						
NOS (per 1 point)	13 (4, 6, 7, 10, 11, 13, 17, 22, 24, 28, 29, 30, 32)	0.99 (0.84-1.17)	0.914	2 (8, 18)	1.15 (0.61-2.15)	0.444
**Study Characteristics**						
Study design (cohort vs. case-control)	13 (4, 6, 7, 10, 11, 13, 17, 22, 24, 28, 29, 30, 32)	0.83 (0.65-1.07)	0.141	2 (8, 18)	NA	NA
Sample size (<5,000 vs. ≥5,000)	13 (4, 6, 7, 10, 11, 13, 17, 22, 24, 28, 29, 30, 32)	0.97 (0.72-1.30)	0.833	2 (8, 18)	1.14(0.23-5.56)	0.763
Study locations (North America vs. non-North America)	13 (4, 6, 7, 10, 11, 13, 17, 22, 24, 28, 29, 30, 32)	1.16 (0.89-1.50)	0.244	2 (8, 18)	0.50 (0.02-11.50)	0.444
**Baseline Study Level**						
Age (mean, per 1 year)	10 (4, 6, 7, 10, 22, 24, 28, 29, 30, 32)	1.02 (0.99-1.06)	0.191	2 (8, 18)	1.06 (0.80-1.42)	0.444
Male sex (per %)	13 (4, 6, 7, 10, 11, 13, 17, 22, 24, 28, 29, 30, 32)	1.00 (0.98-1.01)	0.581	2 (8, 18)	0.92 (0.64-1.33)	0.444
Diabetes (per %)	7 (4, 7, 17, 24, 28, 29, 32)	1.00 (0.98-1.02)	0.970	NA	NA	NA
Atrial fibrillation (per %)	8 (6, 7, 11, 17, 24, 28, 29, 32)	1.00 (0.99-1.01)	0.714	NA	NA	NA
Chronic heart failure (per %)	7 (4, 17, 24, 28, 29, 30, 32)	1.00 (0.99-1.01)	0.735	1 (8)	NA	NA
History of bleeding (per %)	8 (4, 7, 17, 22, 28, 29, 30, 32)	1.00 (0.99-1.02)	0.747	1 (8)	NA	NA
Patients received SRIs (per %)	12 (4, 6, 7, 10, 11, 17, 22, 24, 28, 29, 30, 32)	1.00 (0.99-1.01)	0.486	1 (8)	NA	NA
NSAIDs (per %)	8 (4, 6, 22, 24, 28, 29, 30, 32)	**1.01 (1.00-1.02)**	**0.018**	1 (8)	NA	NA
Corticosteroids (per %)	5 (4, 6, 11, 28, 30)	1.01 (0.89-1.15)	0.795	1 (8)	NA	NA
PPIs/H2RAs (per %)	5 (4, 7, 28, 29, 32)	1.01 (0.99-1.03)	0.161	1 (8)	NA	NA

[a]Effect size for each variable of interest reflecting unit change.

Abbreviations: H2RAs, histamine H2-receptor antagonists; NA, not applicable; NOS, Newcastle-Ottawa scale; NSAIDs, nonsteroidal anti-inflammatory drugs; PPIs, proton pump inhibitors; SRIs, serotonin reuptake inhibitors.

eTable 16. Meta-Regression of Included Studies: Brain Hemorrhage (Continued)

Covariate	Anticoagulant			Antiplatelet		
	No. of Studies (Ref.)	Odd Ratio (95% CI)[a]	*P* Value	No. of Studies (Ref.)	Odd Ratio (95% CI)[a]	*P* Value
**Risk of Bias Assessment**						
NOS (per 1 point)	10 (2, 4, 13, 17, 21, 23, 27, 28, 29, 30)	0.78 (0.49-1.24)	0.266	3 (2, 21, 27)	0.86 (0.26-2.89)	0.657
**Study Characteristics**						
Study design (cohort vs. case-control)	10 (2, 4, 13, 17, 21, 23, 27, 28, 29, 30)	1.10 (0.55-2.18)	0.765	3 (2, 21, 27)	NA	NA
Sample size (<5,000 vs. ≥5,000)	10 (2, 4, 13, 17, 21, 23, 27, 28, 29, 30)	0.72 (0.35-1.50)	0.345	3 (2, 21, 27)	1.42 (0.66-3.06)	0.190
Study locations (North America vs. non-North America)	10 (2, 4, 13, 17, 21, 23, 27, 28, 29, 30)	1.00 (0.46-2.16)	0.998	3 (2, 21, 27)	1.42 (0.66-3.06)	0.190
**Baseline Study Level**						
Age (mean, per 1 year)	7 (4, 21, 23, 27, 28, 29, 30)	0.98 (0.81-1.17)	0.752	2 (21, 27)	1.05 (0.62-1.79)	0.444
Male sex (per %)	9 (4, 13, 17, 21, 23, 27, 28, 29, 30)	**0.96 (0.93-0.99)**	**0.016**	2 (21, 27)	1.01 (0.92-1.11)	0.444
Diabetes (per %)	7 (4, 17, 21, 23, 27, 28, 29)	1.04 (1.00-1.08)	0.056	2 (21, 27)	0.81 (0.08-7.81)	0.444
Atrial fibrillation (per %)	6 (17, 21, 23, 27, 28, 29)	1.00 (0.98-1.02)	0.898	2 (21, 27)	1.03 (0.73-1.46)	0.444
Chronic heart failure (per %)	6 (4, 17, 21, 28, 29, 30)	1.00 (0.97-1.02)	0.976	1 (21)	NA	NA
History of bleeding (per %)	6 (4, 17, 21, 28, 29, 30)	0.96 (0.89-1.03)	0.200	1 (21)	NA	NA
Patients received SRIs (per %)	9 (2, 4, 17, 21, 23, 27, 28, 29, 30)	0.99 (0.85-1.15)	0.855	3 (2, 21, 27)	0.98 (0.86-1.11)	0.481
NSAIDs (per %)	6 (4, 21, 27, 28, 29, 30)	1.03 (0.99-1.07)	0.091	2 (21, 27)	0.98 (0.83-1.17)	0.444
Corticosteroids (per %)	3 (4, 28, 30)	0.92 (0.57-1.50)	0.557	NA	NA	NA
PPIs/H2RAs (per %)	3 (4, 28, 29)	1.04 (0.92-1.18)	0.304	NA	NA	NA

[a]Effect size for each variable of interest reflecting unit change.

Abbreviations: H2RAs, histamine H2-receptor antagonists; NA, not applicable; NOS, Newcastle-Ottawa scale; NSAIDs, nonsteroidal anti-inflammatory drugs; PPIs, proton pump inhibitors; SRIs, serotonin reuptake inhibitors.

eTable 16. Meta-Regression of Included Studies: GI Bleeding (Continued)

Covariate	Anticoagulant			Antiplatelet		
	No. of Studies (Ref.)	Odd Ratio (95% CI)[a]	*P* Value	No. of Studies (Ref.)	Odd Ratio (95% CI)[a]	*P* Value
**Risk of Bias Assessment**						
NOS (per 1 point)	10 (1, 3, 4, 9, 13, 19, 28, 29, 31, 32)	0.95 (0.86-1.05)	0.282	5 (3, 5, 8, 12, 26)	0.93 (0.21-4.20)	0.887
**Study Characteristics**						
Study design (cohort vs. case-control)	10 (1, 3, 4, 9, 13, 19, 28, 29, 31, 32)	1.08 (0.80-1.47)	0.559	5 (3, 5, 8, 12, 26)	1.46 (0.88-2.44)	0.100
Sample size (<5,000 vs. ≥5,000)	10 (1, 3, 4, 9, 13, 19, 28, 29, 31, 32)	1.07 (0.67-1.72)	0.747	5 (3, 5, 8, 12, 26)	0.73 (0.24-2.23)	0.439
Study locations (North America vs. non-North America)	10 (1, 3, 4, 9, 13, 19, 28, 29, 31, 32)	1.01 (0.72-1.41)	0.961	5 (3, 5, 8, 12, 26)	0.85 (0.32-2.25)	0.641
**Baseline Study Level**						
Age (mean, per 1 year)	6 (1, 4, 28, 29, 31, 32)	0.98 (0.96-1.01)	0.187	4 (5, 8, 12, 26)	0.97 (0.80-1.18)	0.630
Male sex (per %)	9 (3, 4, 9, 13, 19, 28, 29, 31, 32)	0.99 (0.98-1.01)	0.229	5 (3, 5, 8, 12, 26)	1.01 (0.95-1.08)	0.687
Diabetes (per %)	6 (1, 4, 19, 28, 29, 32)	1.02 (1.00-1.05)	0.066	3 (5, 12, 26)	1.01 (0.86-1.17)	0.674
Atrial fibrillation (per %)	4 (19, 28, 29, 32)	1.00 (0.98-1.02)	0.853	NA	NA	NA
Chronic heart failure (per %)	5 (4, 19, 28, 29, 32)	1.01 (0.97-1.05)	0.639	2 (5, 8)	NA	NA
History of bleeding (per %)	6 (1, 4, 9, 28, 29, 32)	1.01 (0.99-1.02)	0.433	3 (5, 8, 12)	1.01 (0.74-1.38)	0.707
Patients received SRIs (per %)	9 (1, 3, 4, 9, 19, 28, 29, 31, 32)	1.01 (1.00-1.03)	0.143	5 (3, 5, 8, 12, 26)	0.98 (0.86-1.11)	0.599
NSAIDs (per %)	7 (1, 3, 4, 9, 28, 29, 32)	1.01 (0.99-1.03)	0.387	5 (3, 5, 8, 12, 26)	1.01 (0.95-1.08)	0.591
Corticosteroids (per %)	4 (1, 4, 19, 28)	1.01 (1.00-1.02)	0.099	4 (5, 8, 12, 26)	1.03 (0.74-1.44)	0.752
PPIs/H2RAs (per %)	8 (1, 3, 4, 9, 19, 28, 29, 32)	1.01 (0.99-1.02)	0.198	4 (3, 5, 8, 12)	1.01 (0.87-1.17)	0.842

[a]Effect size for each variable of interest reflecting unit change.

Abbreviations: GI, gastrointestinal; H2RAs, histamine H2-receptor antagonists; NA, not applicable; NOS, Newcastle-Ottawa scale; NSAIDs, nonsteroidal anti-inflammatory drugs; PPIs, proton pump inhibitors; SRIs, serotonin reuptake inhibitors.

eTable 16. Meta-Regression of Included Studies: Any Bleeding (Continued)

Covariate	Anticoagulant			Antiplatelet		
	No. of Studies (Ref.)	Odd Ratio (95% CI)[a]	*P* Value	No. of Studies (Ref.)	Odd Ratio (95% CI)[a]	*P* Value
**Risk of Bias Assessment**						
NOS (per 1 point)	23 (1, 2, 3, 4, 6, 7, 9, 10, 11, 13, 16, 17, 19, 21, 22, 23, 24, 27, 28, 29, 30, 31, 32)	0.95 (0.86-1.04)	0.251	11 (2, 3, 5, 8, 12, 15, 18, 21, 25, 26, 27)	1.03 (0.94-1.14)	0.490
**Study Characteristics**						
Study design (cohort vs. case-control)	23 (1, 2, 3, 4, 6, 7, 9, 10, 11, 13, 16, 17, 19, 21, 22, 23, 24, 27, 28, 29, 30, 31, 32)	1.15 (0.89-1.49)	0.277	11 (2, 3, 5, 8, 12, 15, 18, 21, 25, 26, 27)	1.17 (0.93-1.46)	0.165
Sample size (<5,000 vs. ≥5,000)	23 (1, 2, 3, 4, 6, 7, 9, 10, 11, 13, 16, 17, 19, 21, 22, 23, 24, 27, 28, 29, 30, 31, 32)	0.89 (0.67-1.17)	0.382	11 (2, 3, 5, 8, 12, 15, 18, 21, 25, 26, 27)	1.13 (0.85-1.49)	0.377
Study locations (North America vs. non-North America)	23 (1, 2, 3, 4, 6, 7, 9, 10, 11, 13, 16, 17, 19, 21, 22, 23, 24, 27, 28, 29, 30, 31, 32)	0.96 (0.73-1.25)	0.737	11 (2, 3, 5, 8, 12, 15, 18, 21, 25, 26, 27)	0.99 (0.80-1.24)	0.935
**Baseline Study Level**						
Age (mean, per 1 year)	15 (1, 4, 6, 7, 10, 21, 22, 23, 24, 27, 28, 29, 30, 31, 32)	0.99 (0.96-1.02)	0.453	8 (5, 8, 12, 18, 21, 25, 26, 27)	1.03 (1.00-1.06)	0.065
Male sex (per %)	20 (3, 4, 6, 7, 9, 10, 11, 13, 17, 19, 21, 22, 23, 24, 27, 28, 29, 30, 31, 32)	0.99 (0.98-1.00)	0.080	9 (3, 5, 8, 12, 18, 21, 25, 26, 27)	1.01 (0.99-1.02)	0.353
Diabetes (per %)	12 (1, 4, 7, 17, 19, 21, 23, 24, 27, 28, 29, 32)	1.01 (0.99-1.03)	0.231	7 (5, 12, 18, 21, 25, 26, 27)	1.00 (0.99-1.01)	0.855
Atrial fibrillation (per %)	12 (6, 7, 11, 17, 19, 21, 23, 24, 27, 28, 29, 32)	1.00 (1.00-1.01)	0.141	2 (21, 27)	1.03 (0.73-1.46)	0.444

[a]Effect size for each variable of interest reflecting unit change.

Abbreviations: H2RAs, histamine H2-receptor antagonists; NA, not applicable; NOS, Newcastle-Ottawa scale; NSAIDs, nonsteroidal anti-inflammatory drugs; PPIs, proton pump inhibitors; SRIs, serotonin reuptake inhibitors.

eTable 16. Meta-Regression of Included Studies: Any Bleeding (Continued)

Covariate	Anticoagulant			Antiplatelet		
	No. of Studies (Ref.)	Odd Ratio (95% CI)[a]	*P* Value	No. of Studies (Ref.)	Odd Ratio (95% CI)[a]	*P* Value
**Baseline Study Level (continued)**						
Chronic heart failure (per %)	9 (4, 17, 19, 21, 24, 28, 29, 30, 32)	1.00 (0.99-1.01)	0.849	4 (5, 8, 21, 25)	1.02 (0.99-1.04)	0.145
History of bleeding (per %)	11 (1, 4, 7, 9, 17, 21, 22, 28, 29, 30, 32)	1.00 (0.99-1.02)	0.541	4 (5, 8, 12, 21)	1.01 (0.94-1.09)	0.725
Patients received SRIs (per %)	23 (1, 2, 3, 4, 6, 7, 9, 10, 11, 16, 17, 19, 21, 22, 23, 24, 27, 28, 29, 30, 31, 32)	1.00 (0.99-1.01)	0.411	10 (2, 3, 5, 8, 12, 15, 21, 25, 26, 27)	0.98 (0.96-1.00)	0.071
NSAIDs (per %)	13 (1, 3, 4, 6, 9, 21, 22, 24, 27, 28, 29, 30, 32)	1.01 (1.00-1.03)	0.072	7 (3, 5, 8, 12, 21, 26, 27)	0.99 (0.97-1.01)	0.300
Corticosteroids (per %)	7 (1, 4, 6, 11, 19, 28, 30)	1.01 (1.00-1.02)	0.141	4 (5, 8, 12, 26)	1.03 (0.86-1.24)	0.664
PPIs/H2RAs (per %)	9 (1, 3, 4, 7, 9, 19, 28, 29, 32)	1.01 (1.00-1.02)	0.150	4 (3, 5, 8, 12)	1.02 (0.96-1.08)	0.433

[a]Effect size for each variable of interest reflecting unit change.

Abbreviations: H2RAs, histamine H2-receptor antagonists; NA, not applicable; NOS, Newcastle-Ottawa scale; NSAIDs, nonsteroidal anti-inflammatory drugs; PPIs, proton pump inhibitors; SRIs, serotonin reuptake inhibitors.

eTable 17. Meta-Analysis of Included Studies with Calibration for Publication Bias[a]

Bleeding Complication	No. of Studies (Reference)	*P* Value for Begg's Test	*P* Value for Egger's Test	Odds Ratio (95% CI)	*P* Value	Heterogeneity			
						Q Statistic	*P* Value	I^2 Index (95% CI)	τ^2
**Major Bleeding**									
Anticoagulation therapy	13 (4, 6, 7, 10, 11, 13, 17, 22, 24, 28, 29, 30, 32)	0.092	0.006	1.35 (1.19-1.53)	<0.001	36.78	0.006	51.1% (4.2-69.9)	0.028
Antiplatelet therapy	2 (8, 18)	0.734	0.466	Not detected	NA	NA	NA	NA	NA
**Brain Hemorrhage**									
Anticoagulation therapy	10 (2, 4, 13, 17, 21, 23, 27, 28, 29, 30)	0.034	0.010	1.11 (0.86-1.44)	0.411	44.62	<0.001	66.4% (36.2-78.9)	0.140
Antiplatelet therapy	3 (2, 21, 27)	0.308	0.048	Not detected	NA	NA	NA	NA	NA
**GI Bleeding**									
Anticoagulation therapy	10 (1, 3, 4, 9, 13, 19, 28, 29, 31, 32)	0.876	0.731	1.32 (1.18-1.47)	<0.001	16.95	0.109	35.1% (0.0-66.1)	0.011
Antiplatelet therapy	5 (3, 5, 8, 12, 26)	0.806	0.078	1.20 (0.98-1.46)	0.073	8.29	0.218	27.6% (0.0-69.0)	0.019
**Any Bleeding**									
Anticoagulation therapy	23 (1, 2, 3, 4, 6, 7, 9, 10, 11, 13, 16, 17, 19, 21, 22, 23, 24, 27, 28, 29, 30, 31, 32)	0.043	0.007	1.28 (1.15-1.43)	<0.001	109.70	<0.001	69.9% (55.8-78.0)	0.053
Antiplatelet therapy	11 (2, 3, 5, 8, 12, 15, 18, 21, 25, 26, 27)	0.827	0.878	1.12 (1.02-1.22)	0.019	27.80	0.033	42.4% (0.0-66.4)	0.010

[a]Calibration for publication bias was carried out, if indicated in trim and fill analysis.

Abbreviations: CI, confidence interval; GI, gastrointestinal; NA, not applicable.

eFigure 1. Funnel Plot of Included Studies in the Meta-Analysis

A. Major Bleeding: Anticoagulant Therapy

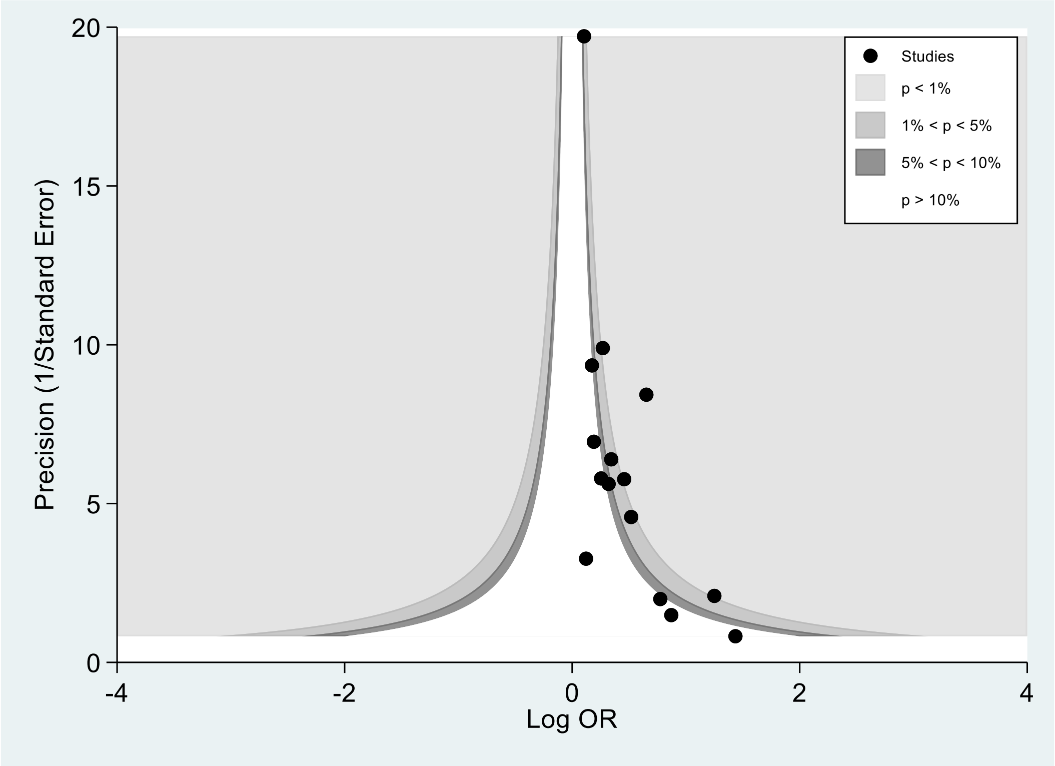

B. Major Bleeding: Antiplatelet Therapy

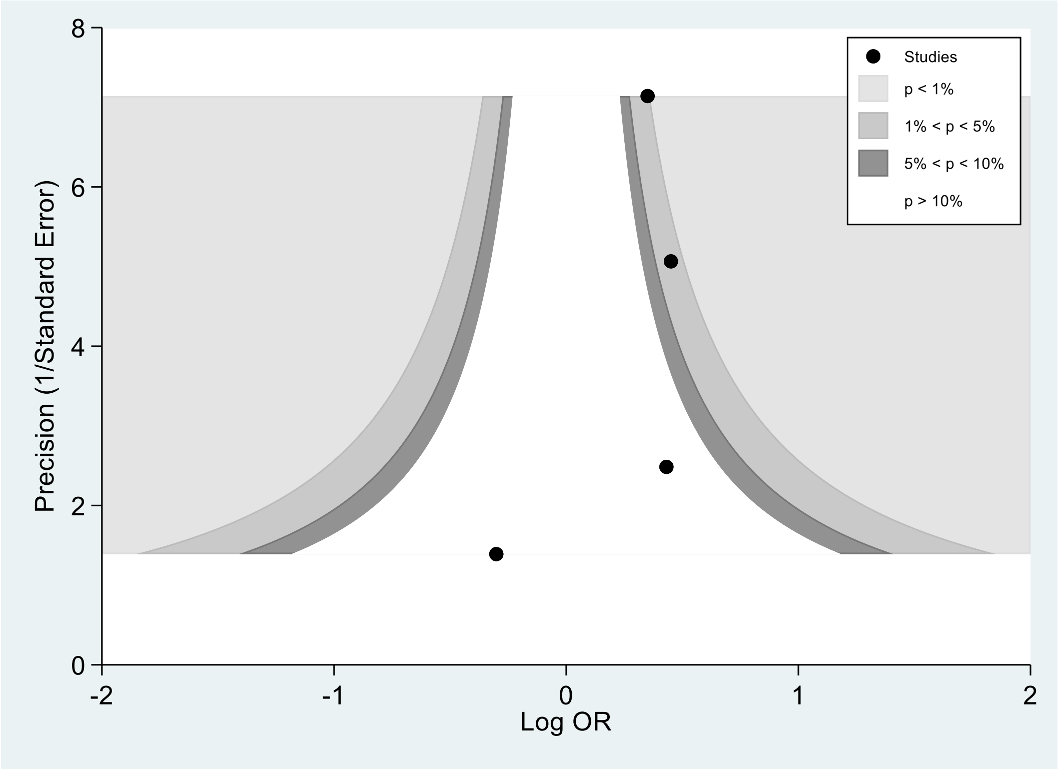

eFigure 1. Funnel Plot of Included Studies in the Meta-Analysis (Continued)

C. Brain Hemorrhage: Anticoagulant Therapy

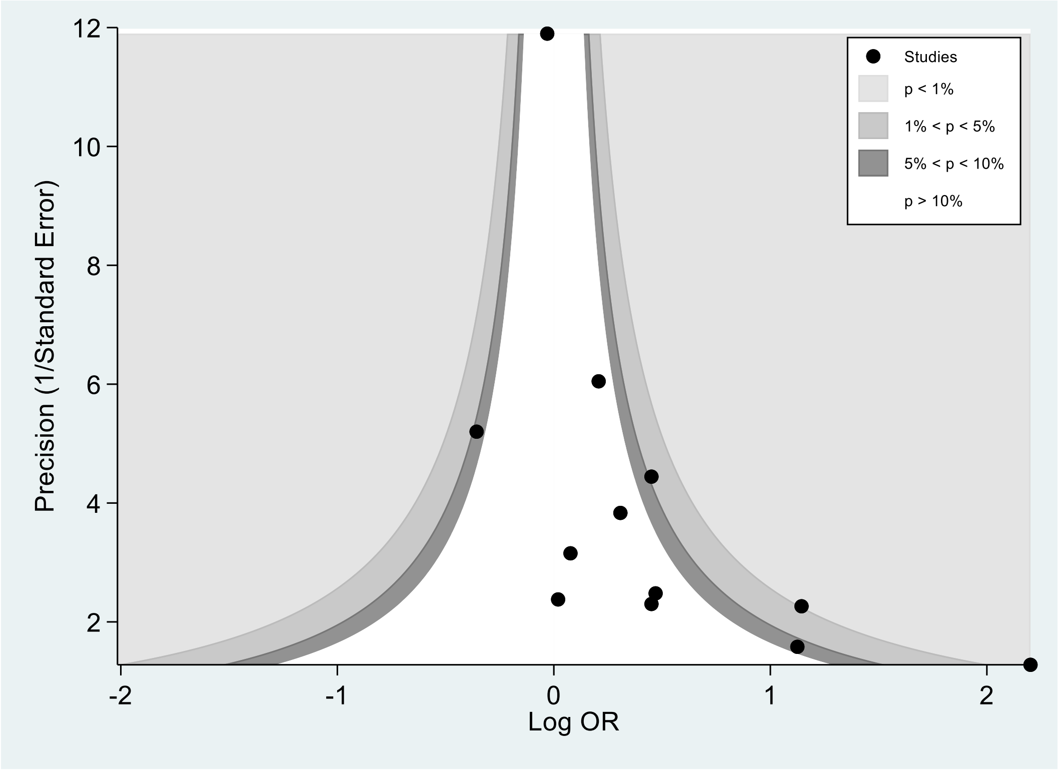

D. Brain Hemorrhage: Antiplatelet Therapy

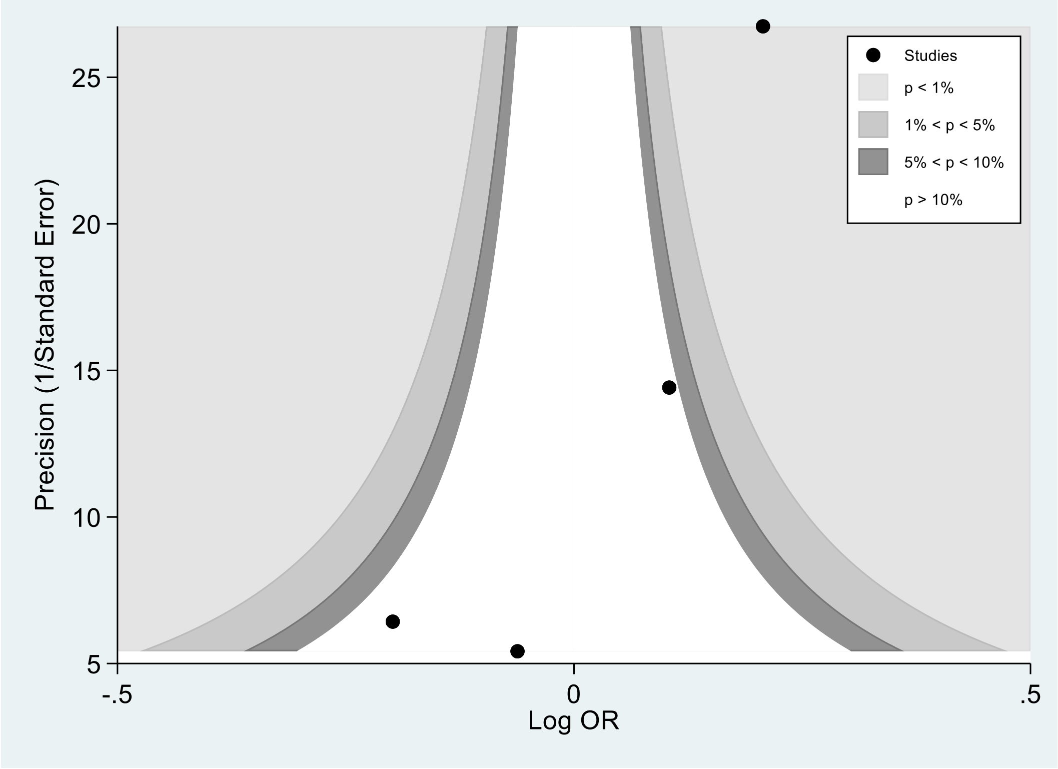

eFigure 1. Funnel Plot of Included Studies in the Meta-Analysis (Continued)

E. Gastrointestinal Bleeding: Anticoagulant Therapy

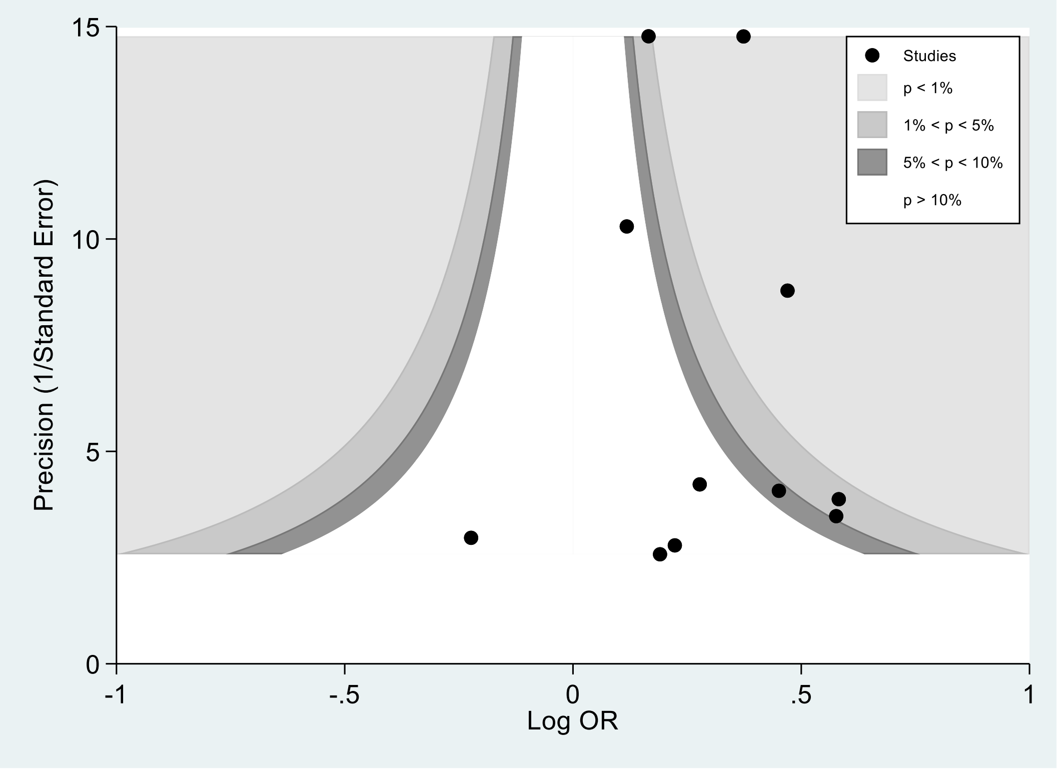

F. Gastrointestinal Bleeding: Antiplatelet Therapy

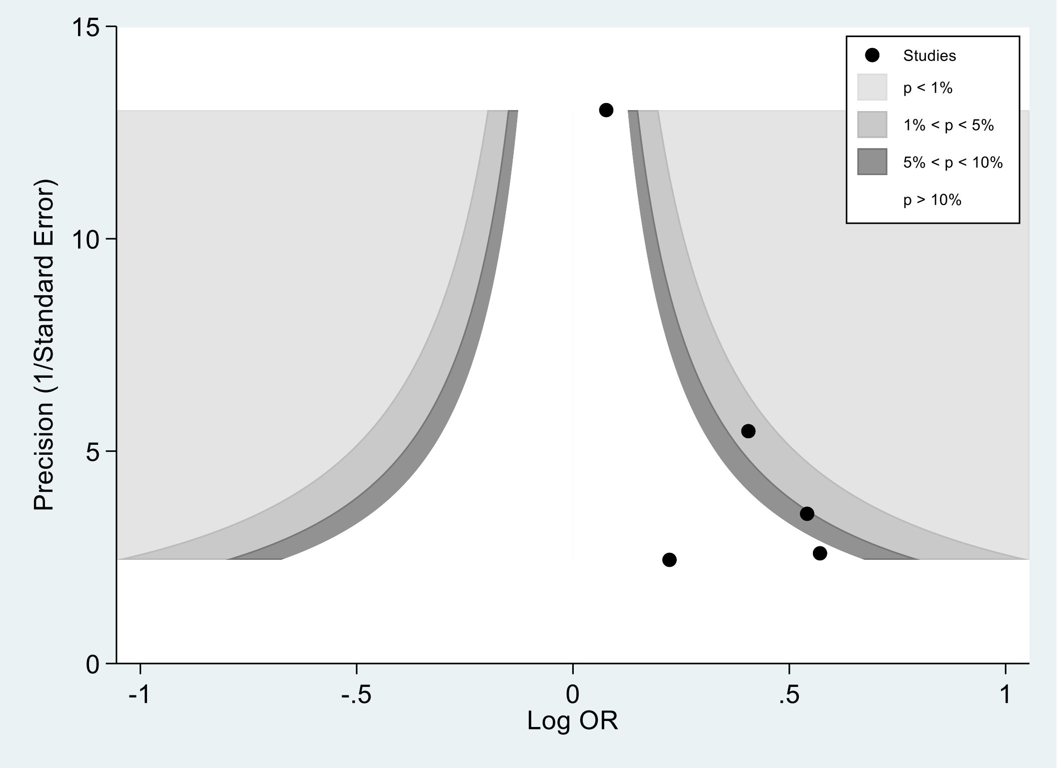

eFigure 1. Funnel Plot of Included Studies in the Meta-Analysis (Continued)

G. Any Bleeding: Anticoagulant Therapy

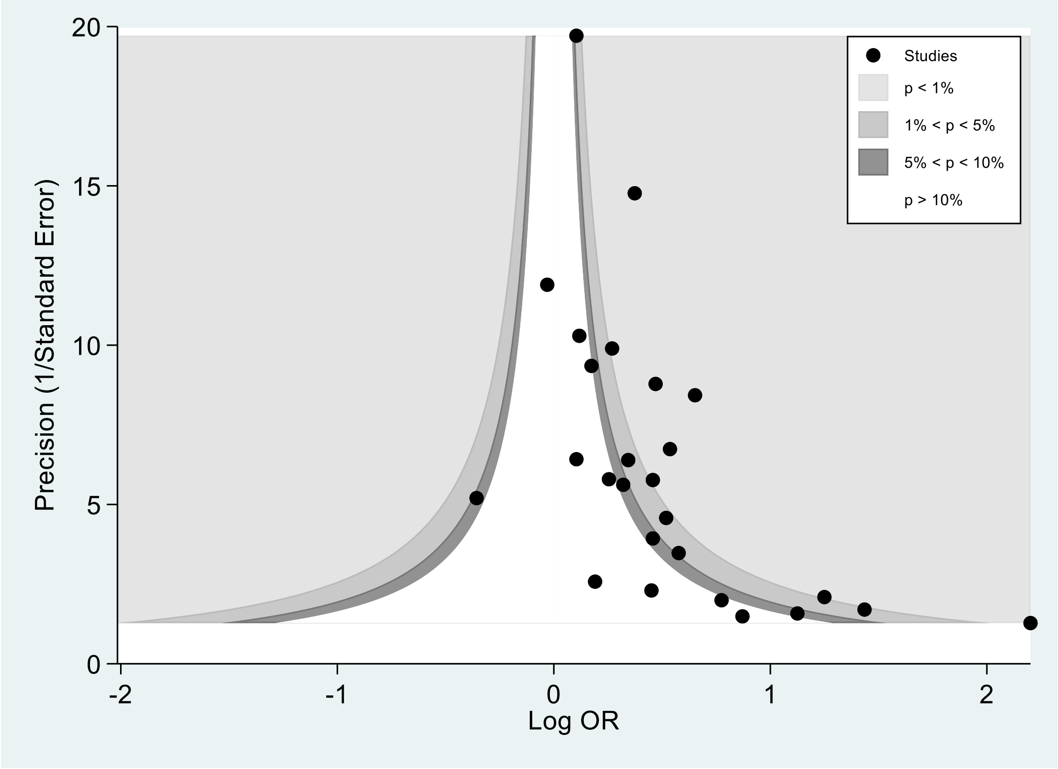

H. Any Bleeding: Antiplatelet Therapy

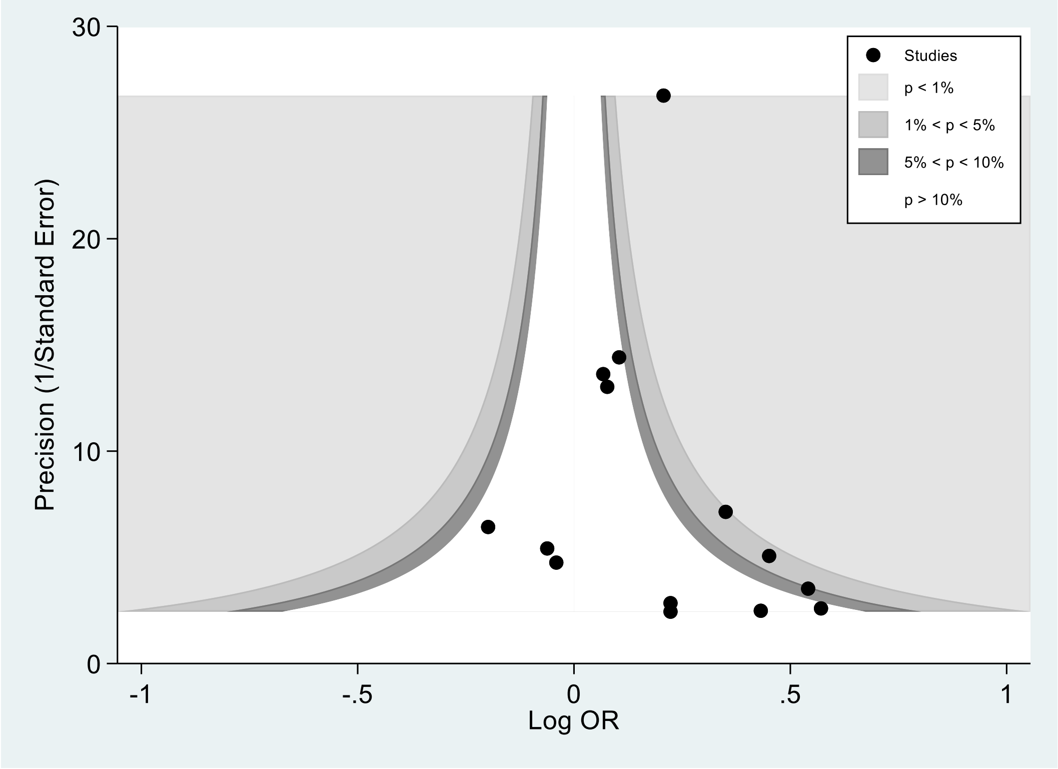

eReferences

1. Kurdyak PA, Juurlink DN, Kopp A, Herrmann N, Mamdani MM. Antidepressants, warfarin, and the risk of hemorrhage. *J Clin Psychopharmacol.* 2005;25(6):561-564.
2. Kharofa J, Sekar P, Haverbusch M, et al. Selective serotonin reuptake inhibitors and risk of hemorrhagic stroke. *Stroke.* 2007;38(11):3049-3051.
3. de Abajo FJ, Garcia-Rodriguez LA. Risk of upper gastrointestinal tract bleeding associated with selective serotonin reuptake inhibitors and venlafaxine therapy: interaction with nonsteroidal anti-inflammatory drugs and effect of acid-suppressing agents. *Arch Gen Psychiatry.* 2008;65(7):795-803.
4. Schalekamp T, Klungel OH, Souverein PC, de Boer A. Increased bleeding risk with concurrent use of selective serotonin reuptake inhibitors and coumarins. *Arch Intern Med.* 2008;168(2):180-185.
5. Dall M, Schaffalitzky de Muckadell OB, Lassen AT, Hansen JM, Hallas J. An association between selective serotonin reuptake inhibitor use and serious upper gastrointestinal bleeding. *Clin Gastroenterol Hepatol.* 2009;7(12):1314-1321.
6. Wallerstedt SM, Gleerup H, Sundstrom A, Stigendal L, Ny L. Risk of clinically relevant bleeding in warfarin-treated patients--influence of SSRI treatment. *Pharmacoepidemiol Drug Saf.* 2009;18(5):412-416.
7. Cochran KA, Cavallari LH, Shapiro NL, Bishop JR. Bleeding incidence with concomitant use of antidepressants and warfarin. *Ther Drug Monit.* 2011;33(4):433-438.
8. Labos C, Dasgupta K, Nedjar H, Turecki G, Rahme E. Risk of bleeding associated with combined use of selective serotonin reuptake inhibitors and antiplatelet therapy following acute myocardial infarction. *CMAJ.* 2011;183(16):1835-1843.
9. Schelleman H, Brensinger CM, Bilker WB, Hennessy S. Antidepressant-warfarin interaction and associated gastrointestinal bleeding risk in a case-control study. *PLoS One.* 2011;6(6):e21447.
10. Vitry AI, Roughead EE, Ramsay EN, et al. Major bleeding risk associated with warfarin and co-medications in the elderly population. *Pharmacoepidemiol Drug Saf.* 2011;20(10):1057-1063.
11. Baillargeon J, Holmes HM, Lin YL, Raji MA, Sharma G, Kuo YF. Concurrent use of warfarin and antibiotics and the risk of bleeding in older adults. *Am J Med.* 2012;125(2):183-189.
12. Lin CC, Hu HY, Luo JC, et al. Risk factors of gastrointestinal bleeding in clopidogrel users: a nationwide population-based study. *Aliment Pharmacol Ther.* 2013;38(9):1119-1128.

13. Mosholder AD, Racoosin JA, Young S, et al. Bleeding events following concurrent use of warfarin and oseltamivir by Medicare beneficiaries. *Ann Pharmacother.* 2013;47(11):1420-1428.
14. Seitz DP, Bell CM, Gill SS, et al. Risk of perioperative blood transfusions and postoperative complications associated with serotonergic antidepressants in older adults undergoing hip fracture surgery. *J Clin Psychopharmacol.* 2013;33(6):790-798.
15. Giang K, Mouwakeh H, Stubbs M, Lacro J. Assessing bleeding associated with combined use of SSRI and dual antiplatelet therapy. *Crit Care Med.* 2014;42(12 Suppl).
16. Nguyen TN, Bird J, Furrh R, Jones C, Gentry C. Retrospective review of bleeding incidence associated with concomitant use of warfarin and selective serotonin reuptake inhibitors (SSRIs) in a veteran population. *Pharmacotherapy.* 2014;34(6):e86.
17. Quinn GR, Singer DE, Chang Y, et al. Effect of selective serotonin reuptake inhibitors on bleeding risk in patients with atrial fibrillation taking warfarin. *Am J Cardiol.* 2014;114(4):583-586.
18. Rashid H, Hu J, Chan J, et al. Bleeding outcomes with selective serotonin reuptake inhibitors in combination with dual antiplatelet therapy following acute coronary syndrome. *Heart Lung Circ.* 2016;25:S33-S34.
19. Lai JH, Vaidya S, Sudat S, Pressman A, Gerson LB. Prevalence and risk factors for gastrointestinal bleeding in novel oral anticoagulant users. *Gastroenterology.* 2017;152 (5 Supplement 1):S475-S476.
20. Laursen SB, Leontiadis GI, Stanley AJ, Hallas J, Schaffalitzky de Muckadell OB. The use of selective serotonin receptor inhibitors (SSRIs) is not associated with increased risk of endoscopy-refractory bleeding, rebleeding or mortality in peptic ulcer bleeding. *Aliment Pharmacol Ther.* 2017;46(3):355-363.
21. Renoux C, Vahey S, Dell'Aniello S, Boivin JF. Association of selective serotonin reuptake inhibitors with the risk for spontaneous intracranial hemorrhage. *JAMA Neurology.* 2017;74(2):173-180.
22. Samuel NG, Seifert CF. Risk of Bleeding in Patients on Full-Dose Enoxaparin With Venous Thromboembolism and Selective Serotonin Reuptake Inhibitors. *Ann Pharmacother.* 2017;51(3):226-231.
23. Scheitz JF, Turc G, Kujala L, et al. Intracerebral Hemorrhage and Outcome After Thrombolysis in Stroke Patients Using Selective Serotonin-Reuptake Inhibitors. *Stroke.* 2017;48(12):3239-3244.
24. Quinn GR, Hellkamp AS, Hankey GJ, et al. Selective Serotonin Reuptake Inhibitors and Bleeding Risk in Anticoagulated Patients With Atrial Fibrillation: An Analysis From the ROCKET AF Trial. *J Am Heart Assoc.* 2018;7(15):e008755.

25. Iasella CJ, Kreider MS, Huang L, Coons JC, Stevenson JM. Effect of Selective Serotonin Reuptake Inhibitors on Cardiovascular Outcomes After Percutaneous Coronary Intervention: A Retrospective Cohort Study. *Clin Drug Investig.* 2019;39(6):543-551.
26. Luo PJ, Lin XH, Lin CC, et al. Risk factors for upper gastrointestinal bleeding among aspirin users: An old issue with new findings from a population-based cohort study. *J Formos Med Assoc.* 2019;118(5):939-944.
27. Gaist D, García Rodríguez LA, Hald SM, et al. Antidepressant drug use and subdural hematoma risk. J Thromb Haemost. 2020;18(2):318-327.
28. Komen JJ, Hjemdahl P, Mantel-Teeuwisse AK, Klungel OH, Wettermark B, Forslund T. Concomitant Anticoagulant and Antidepressant Therapy in Atrial Fibrillation Patients and Risk of Stroke and Bleeding. Clin Pharmacol Ther. 2020;107(1):287-294.
29. Lee MT, Park KY, Kim MS, You SH, Kang YJ, Jung SY. Concomitant Use of NSAIDs or SSRIs with NOACs Requires Monitoring for Bleeding. Yonsei Med J. 2020;61(9):741-749.
30. Marchena PJ, Tzoran I, Brenner B, et al. Psychotropic Drugs and Outcome in Patients Receiving Anticoagulant Therapy for Venous Thromboembolism. Thromb Haemost. 2020;120(4):620-626.
31. Mawardi G, Markman TM, Muslem R, et al. SSRI/SNRI Therapy is Associated With a Higher Risk of Gastrointestinal Bleeding in LVAD Patients. Heart Lung Circ. 2020;29(8):1241-1246.
32. Zhang Y, Souverein PC, Gardarsdottir H, van den Ham HA, Maitland-van der Zee AH, de Boer A. Risk of major bleeding among users of direct oral anticoagulants combined with interacting drugs: A population-based nested case-control study. Br J Clin Pharmacol. 2020;86(6):1150-1164.
33. Wells G, Shea B, O'Connell D, et al. The Newcastle-Ottawa Scale (NOS) for assessing the quality of nonrandomised studies in meta-analyses. 2020; http://www.ohri.ca/programs/clinical_epidemiology/oxford.asp. Accessed December 2, 2020.
34. Sterne JA, Hernán MA, Reeves BC, et al. ROBINS-I: a tool for assessing risk of bias in non-randomised studies of interventions. BMJ. 2016;355:i4919.
35. Balshem H, Helfand M, Schünemann HJ, et al. GRADE guidelines: 3. Rating the quality of evidence. J Clin Epidemiol. 2011;64(4):401-406.
